# Carbon-nanotube reinforcement of DNA-silica nanocomposites yields programmable and cell-instructive biocoatings

**DOI:** 10.1038/s41467-019-13381-1

**Published:** 2019-12-04

**Authors:** Yong Hu, Carmen M. Domínguez, Jens Bauer, Simone Weigel, Alessa Schipperges, Claude Oelschlaeger, Norbert Willenbacher, Stephan Keppler, Martin Bastmeyer, Stefan Heißler, Christof Wöll, Tim Scharnweber, Kersten S. Rabe, Christof M. Niemeyer

**Affiliations:** 1 Karlsruhe Institute of Technology (KIT), Institute for Biological Interfaces (IBG 1), Hermann-von-Helmholtz-Platz 1, 76344 Karlsruhe, Eggenstein-Leopoldshafen Germany; 2 Karlsruhe Institute of Technology (KIT), Institute for Mechanical Process Engineering and Mechanics, Gotthard-Franz-Straße 3, 76131 Karlsruhe, Germany; 3 Karlsruhe Institute of Technology (KIT), Department of Cell- and Neurobiology, Zoological Institute, Haid-und-Neu-Straße 9, 76131 Karlsruhe, Germany; 4 Karlsruhe Institute of Technology (KIT), Institute of Functional Interfaces (IFG), Hermann-von-Helmholtz-Platz 1, 76344 Karlsruhe, Eggenstein-Leopoldshafen Germany

**Keywords:** DNA, Biomaterials, Nanocomposites, Biomedical materials, Self-assembly

## Abstract

Biomedical applications require substrata that allow for the grafting, colonization and control of eukaryotic cells. Currently available materials are often limited by insufficient possibilities for the integration of biological functions and means for tuning the mechanical properties. We report on tailorable nanocomposite materials in which silica nanoparticles are interwoven with carbon nanotubes by DNA polymerization. The modular, well controllable and scalable synthesis yields materials whose composition can be gradually adjusted to produce synergistic, non-linear mechanical stiffness and viscosity properties. The materials were exploited as substrata that outperform conventional culture surfaces in the ability to control cellular adhesion, proliferation and transmigration through the hydrogel matrix. The composite materials also enable the construction of layered cell architectures, the expansion of embryonic stem cells by simplified cultivation methods and the on-demand release of uniformly sized stem cell spheroids.

## Abstract (web summary)

DNA composite materials have potential for biomedical sciences; however, control over the materials can be an issue. Here, the authors report on a carbon-nanotube reinforced DNA-silica gel with controllable mechanical properties to steer the attachment, proliferation, migration and release of cells.

## Introduction

The design of novel, programmable “intelligent” cell culture substrates to control the interaction of eukaryotic cells with technical surfaces is of paramount interest for biomedical applications^1^, such as stem cell therapies^2^ or tissue engineering^3,4^. These applications call for biocompatible materials with tunable physical properties in terms of porosity and elasticity, which can be functionalized with biomolecules, such as proteins, peptides, morphogens, and growth factors, and which can be degraded under mild conditions to release the cells or cell aggregates after cultivation. Synthetic hydrogels are very promising in this respect because their three-dimensional (3D) porous structure yields biocompatibility, high-water content, and tissue-like elastic properties that allow for effective permeation of oxygen and nutrients, which is crucial for cellular colonization^5–8^.

While approaches have been developed for the systematic adjustment of the mechanical properties of organic hydrogels by cross-linking with nanoparticles^9–13^, synthetic hydrogels may suffer from potential drawbacks due to adverse effects of chemical ingredients^14^. Hydrogel materials from DNA offer advantages in this respect, as they can be produced using exclusively non-toxic biochemical reactions^15–21^. These biopolymers can also be programmed very efficiently via their nucleic acid sequence in order to install shape memory persistence, molecular recognition capabilities and stimuli responsiveness, for example, to facilitate their degradation with a mild enzymatic treatment. However, the mechanical properties of DNA hydrogels are difficult to modify. A multitude of DNA-functionalized nanoparticles have been produced e.g. from gold^22^, metalloxides^23,24^, silica^25,26^, or carbon nanotubes (CNT)^27^. DNA-decorated silica NPs (SiNP) have unique properties in terms of biocompatibility and synthetic accessibility^28^. Since preliminary studies indicated the utility of CNT in reinforcing nanofiber networks and suggested good compatibility with DNA materials^29–33^, we hypothesized that the combination of SiNP, CNT and DNA hydrogels should be able to produce mechanically adjustable materials that can be used to control cellular functions.

**Fig. 1:**
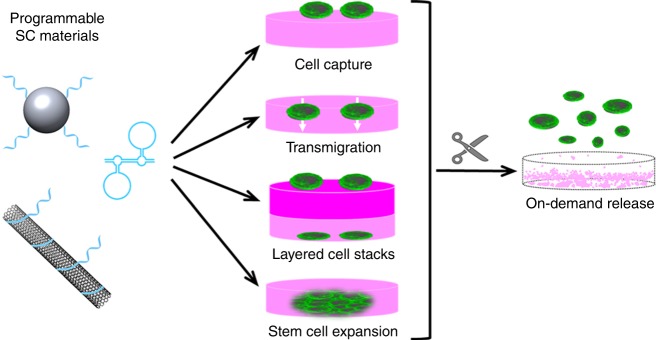
Strategy. The mechanical properties of SiNP/CNT-DNA composites can be adjusted by enzymatic polymerization of DNA-modified nanoparticles. The utility of the resulting “SC materials” for cell-substrate engineering is demonstrated by control of cellular adhesion, proliferation, lateral, and vertical motility, as well as by expansion and release of embryonic stem cells by simplified procedures.

We describe composites in which silica nanoparticles are interwoven with carbon nanotubes by DNA polymerization. The modular, well controllable and scalable synthesis yields materials whose composition can be gradually adjusted to produce synergistic, non-linear mechanical stiffness, and viscosity properties. The materials’ tailorable elastic properties are used to control cellular adhesion, proliferation and transmigration through the hydrogel matrix. We also demonstrate controlled cell release under mild conditions. This allows for the construction of layered cell architectures, the expansion of embryonic stem cells by simplified cultivation methods and the production of uniformly sized stem cell spheroids (Fig. 1).

Since materials libraries can be readily constructed via automated synthesis, we believe that the materials have a high potential for fundamental studies and device applications in biomedical sciences.

## Results

### Polymerization of primer-modified nanoparticles

**Fig. 2:**
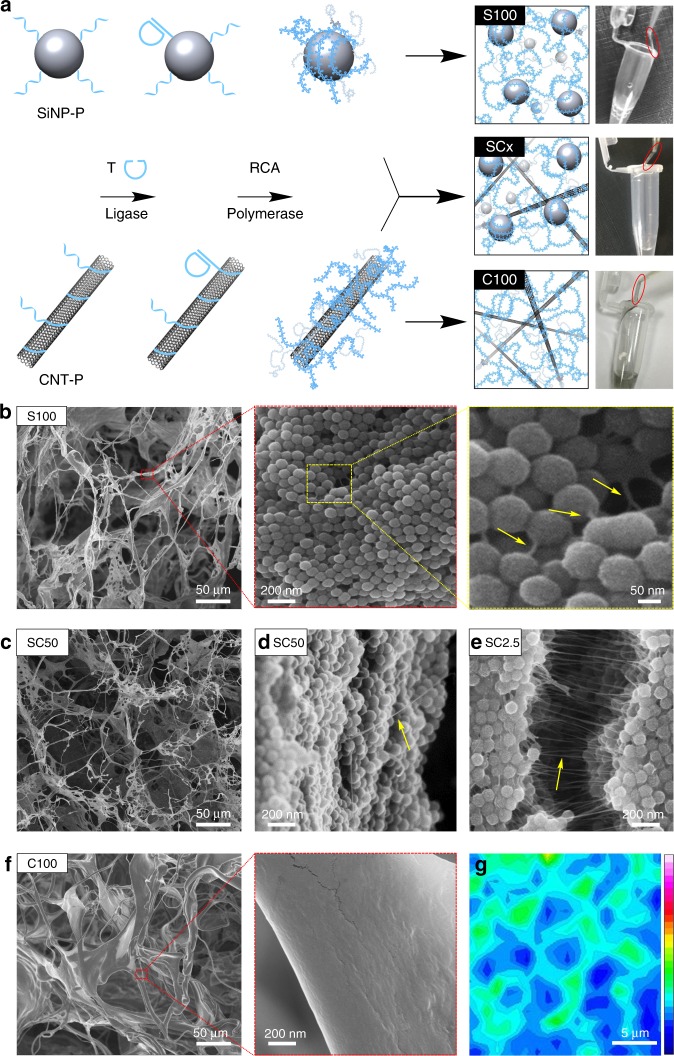
Synthesis of binary and ternary SiNP/CNT-DNA nanocomposite materials. **a** Schematic representation of RCA-based nanocomposite synthesis. DNA oligonucleotide primer-modified SiNP-P and/or CNT-P are used for enzymatic cyclisation of RCA template T. Enzymatic primer extension yields the corresponding binary (S100/C100) or ternary (SCx) DNA nanocomposites. The acronyms of S100/C100/SCx are defined in Table 1. The typical macroscopic viscosity of the materials is shown in representative photographs. **b**–**f** Representative SEM images of composite materials. The arrows point at CNT. **g** Raman map of the intensity of the CNT-G-mode band (integral 1550–1605 cm^−1^) confirming the presence of CNT in C100. The color bar from blue to magenta indicates the gradual increase of signal intensity.

To produce the desired SiNP/CNT-DNA nanocomposites, we employed the rolling-circle amplification (RCA) using single-stranded DNA (ssDNA) modified SiNP and CNT that served as primers for enzymatic extension using a cyclized ssDNA template to crosslink the particles (Fig. 2a). To synthesize the desired SiNP/CNT-DNA nanocomposite materials through RCA, the SiNP and CNT building blocks were functionalised with single-stranded DNA (ssDNA). To this end, zwitterion-stabilized SiNP of 80 nm diameter bearing polyethyleneglycol (PEG) chains in addition to amino-, phosphonate-, and thiol-functional groups were synthesized via hydrolysis of silanes in a microemulsion system, as previously described^26,34^. The SiNP were coupled with aminoalkyl-modified ssDNA primer (aP1) by glutaraldehyde-based cross-linking to obtain primer-modified SiNP-P (Supplementary Fig. 1), which contained about 120 ssDNA molecules grafted to their surface (Supplementary Fig. 2). The same procedure was applied for the preparation of fluorescent dye Cy5 containing core/shell SiNP^26^. Primer-coated CNT (CNT-P in Fig. 2) were prepared from single-walled CNT (1 μm length, 0.83 nm diameter) that were dispersed in an aqueous solution of primer and sonicated on ice, as described elsewhere^35^. This procedure leads to wrapping of the CNT with the ssDNA mediated by π–π stacking interactions. Subsequent purification by centrifugation and ultrafiltration led to pure water-soluble CNT-P displaying a mass ratio of DNA:CNT of about 0.33:1. Atomic force microscopy (AFM) imaging (Supplementary Fig. 3) and Raman spectroscopy (Supplementary Fig. 4) confirmed the presence of monodispersed CNT-P.

To facilitate RCA-based polymerization, the primer-modified nanoparticles were allowed to hybridize with a linear ssDNA oligomer (T, in Fig. 2) that was subjected to ring closure achieved by enzymatic ligation with T4 DNA ligase (Supplementary Figs. 5 and 6) to serve as a template for RCA^36^. The resulting particles were collected by centrifugation and resuspended in RCA reaction mixture containing dNTPs and Phi29 DNA polymerase to facilitate polymerization. The modularity of this synthetic strategy enabled the straightforward generation of a library of differently composed materials that contained only SiNP or CNT (in the following denoted as S100 or C100, respectively) or ternary composites containing both SiNP and CNT, denoted as SCx, where x gives the mass ratio of SiNP-P:CNT-P, as indicated in Table 1. We found that S100 hydrogels containing 4 mg mL^−1^ SiNP after 48 h of polymerization had optimal properties for cell experiments, and a maximum of 320 μg mL^−1^ CNT could be incorporated into pure C100 hydrogels. Higher amounts of CNT led to precipitation from the reaction mixture and hardly reproducible formation of brittle materials. Hence, all ternary composites contained relative fractions of these maximal amounts (Table 1 and Supplementary Table 1).

**Table 1:** Overview on binary and ternary SiNP/CNT-DNA nanocomposite materials.

Entry	Name^a^	[SiNP-P]^b^	[CNT-P]^b^	*G*_0_ (Pa)^c^	*D* (μm^2 ^ s^−1^)^d^	*θ* (^o^)^e^
1	S100	4000	−	3.2 ± 0.4	12.0 ± 0.2	0
2	SC50	4000	80	4.8 ± 0.2	12.4 ± 1.1	0
3	SC25	4000	160	8.5 ± 0.7	12.9 ± 0.2	0
4	SC12.5	4000	320	14.1 ± 1.0	13.2 ± 1.0	0
5	SC6.25	2000	320	10.3 ± 1.0	18.9 ± 2.3	3.6 ± 1.2
6	SC2.5	800	320	3.1 ± 0.2	24.5 ± 3.9	4.0 ± 1.9
7	C100	−	320	2.8 ± 0.2	32.3 ± 4.1	9.7 ± 1.9

^a^Acronyms represent the mass ratio of SiNP-P:CNT-P subjected to RCA polymerization

^b^Given in μg mL^−1^

^c^Elastic plateau modulus determined by multiple particle tracking (MPT) after 48 h RCA reaction time using tracer particles of diameter 0.5 and 1.0 µm for samples 1 and 2–7, respectively.

^d^Molecular diffusion coefficient of FITC-dextran (70 kDa), determined by fluorescence recovery after photobleaching (FRAP) experiment after 48 h RCA reaction time

^e^Static water contact angle (WCA) determined by the sessile drop method after 48 h RCA reaction time and 4 h vacuum drying. All data in (c), (d), and (e) are represented by mean ± standard deviation (S.D.) of triplicate samples

### Characterization of SiNP/CNT-DNA composites

**Fig. 3:**
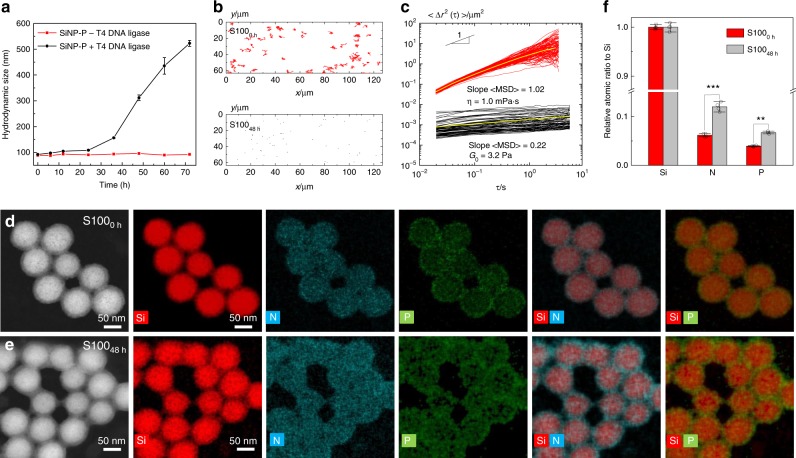
Characterization of SiNP-DNA nanocomposites. **a** Hydrodynamic size of products produced from RCA on SiNP-primer-template (SiNP-P-T) particles in the presence or absence of T4 DNA ligase. All data are represented by mean ± S.D. of triplicate samples. **b** Trajectory maps and **c** MSD values of tracer particles in S100 after 0 and 48 h of RCA. The yellow curves in **c** represent the average MSD. **d**, **e** Representative HAADF-STEM imaging (first column), EDS elemental mapping (second to sixth column) and **f** relative atomic ratios of N and P to Si of S100 after 0 and 48 h of RCA. Data represent mean ± S.D. of the EDS measurements determined from 50 particles in four random areas.

Dynamic light scattering (DLS) was used to monitor the RCA-induced gelation process of S100 (Fig. 3a). It is clearly evident that the hydrodynamic size of SiNP-P increased over time when ligase was added, whereas SiNP-P without ligase treatment displayed negligible change in the hydrodynamic size. The resulting materials revealed the typical viscoelastic properties of hydrogels after reaction times > 12 h (Fig. 2a and Supplementary Figs.  7 and 8).

To quantitatively characterize the mechanical properties of the materials, we used multiple particle tracking microrheology (MPT). Compared to classical rotational rheometry this technique requires a very small sample volume ( < 50 µL), and it is non-destructive, which was the decisive criterion, as the composite materials tore apart even at low rotational frequencies. MPT tracks the Brownian motion of embedded fluorescent tracer particles quantitatively by time-resolved microscopy and the computed trajectories are transformed into mean square displacement (MSD) traces that allow for calculation of the frequency independent storage modulus (*G*_0_). In thermal equilibrium this quantity is directly proportional to the number of chemical and/or physical crosslinks of the entanglement network^37,38^ (Table 1 and Supplementary Fig. 9 and Supplementary Tables 1–5). As indicated by the nearly Gaussian distribution of MSD values at constant lag time *τ*, the composite’s network structure was homogeneous for all investigated systems. It is clearly evident from the variation of the MSD values of tracer particles in Fig. 3b, c that the particles could freely diffuse in solutions of SiNP-P but were restrained by the network of polymerized S100 (see also Supplementary Fig. 9). The corresponding plateau moduli rose with increasing RCA times (Supplementary Table 3). Likewise, the viscoelastic properties of C100 gels depended on the CNT-P concentration **(**Supplementary Table 5). As determined by MPT measurements using differently sized tracer particles, the nanocomposites differ in mesh size (0.2–0.5 µm and 0.5–1.0 µm for S100 and SCx/C100, respectively) (Supplementary Tables 2 and 4). Determination of the molecular diffusion coefficient of FITC-dextran (70 kDa), by fluorescence recovery after photobleaching (FRAP) experiments indicated that the diffusion increases monotonously with increasing proportions of CNT (Table 1 and Supplementary Fig. 10). Of note, about 4–5 times higher diffusion coefficients were determined for a representative small-molecule (FITC-dUTP, ~1 kDa). The data suggest that the mass transport of nutrients in SCx materials is not restricted compared to standard cell culture substrates (see discussion in Supplementary Fig. 10).

The mechanical properties of the various ternary SC materials significantly depended on the mass ratio SiNP-P:CNT-P (Table 1). In fact, *G*_0_ monotonically increased from 4.8 Pa to 14.1 Pa with increasing amount of CNT (SC50, SC25, and SC12.5), i.e., incorporation of CNT increases the nanocomposite’s mechanical stiffness. Likewise, when the concentration of CNT was held constant, increased concentrations of SiNP enhanced the mechanical stiffness of the ternary SC hydrogels from 3.1 to 14.1 Pa for SC2.5 to SC12.5, respectively. Importantly, the *G*_0_ values of several SC materials were substantially higher than those expected from totaling the values of pure S100 (3.2 Pa) and C100 (2.8 Pa). This result clearly demonstrates that the two differently shaped nanoparticles are synergistically determining the elastic properties of the composite materials.

The structural features of the hydrogels were characterized by transmission electron microscopy (TEM, Supplementary Fig. 11), scanning electron microscopy (SEM, Fig. 2b–f and Supplementary Figs. 12–15) and atomic force microscopy (AFM, Supplementary Fig. 16). The materials have an amorphous morphology with a distinctive hierarchical ultrastructure, which clearly reveals the DNA-coated particles that are connected by DNA filaments (Fig. 2b). Detailed analysis by scanning transmission electron microscopy (STEM) coupled with energy-dispersive X-ray spectroscopy (EDS, Fig. 3d–f and Supplementary Fig. 17) confirmed the presence of a dense layer of polymerized DNA that coats and crosslinks the particles in the composite material. The ternary SCx and the binary composite C100 materials showed similar mesoscopic morphology (Fig. 2c, f and Supplementary Figs. 12–14). In several cases, individual CNT could be optically resolved (Fig. 2d, e and Supplementary Figs. 15 and 16) and their presence was also confirmed with Raman microscopy (Fig. 2g).

To further illustrate the modularity of our composite materials design, we demonstrated that fluorescent properties can be readily incorporated in the materials by either using dye-doped SiNP^26^ or, else, by RCA-based incorporation of fluorescent dye-modified deoxynucleotides, such as fluorescein (FITC)-modified dUTP. The resulting materials revealed the expected optical properties (Supplementary Fig. 18) while their morphology remained unchanged (Supplementary Fig. 19). Furthermore, to create responsive materials that can be post-synthetically modified with enzymes^20^, we introduced restriction sites into the nanocomposite’s backbone by encoding stem-loop structures of appropriate sequence in the RCA template (Supplementary Fig. 20). Indeed, resulting DNA hydrogels showed typical features of double-stranded DNA, as determined by thermal melting experiments (Supplementary Fig. 21) and circular dichroism (CD) spectroscopy (Supplementary Fig. 22) and the SiNP/CNT-DNA nanocomposites were efficiently degraded by endonuclease treatment under physiological conditions (Supplementary Fig. 23). While the enzyme-triggered degradation is of great utility to facilitate the controllable on-demand release of adhered or embedded cells, which is required for therapeutic applications and dynamic cell manipulations^39,40^, the nanocomposites showed good stability against non-specific degradation by exonuclease DNase I (Supplementary Fig. 24) and prolonged storage in cell culture media (Supplementary Fig. 25).

### Control of cellular adhesion, proliferation, and migration

**Fig. 4:**
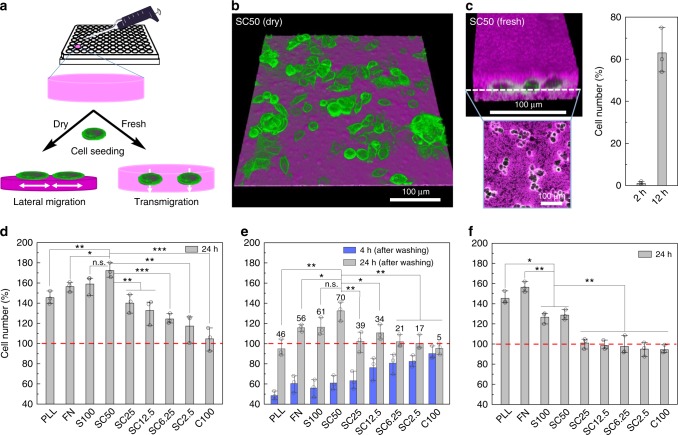
Viability, proliferation, and migration of MCF7_eGFP_ cells adhered on nanocomposite films. **a** Schematics of preparing fully hydrated (“fresh”) or dehydrated (“dried”) nanocomposite films for cell cultivation. **b** Representative fluorescence microscopy image of MCF7_eGFP_ cells (green) after adhesion on dried SC50 (magenta). **c** MCF7_eGFP_ cells transmigrate into fresh SC50, as indicated by the number of cells detected in the central layer of SC50 after 2 h and 12 h, respectively; the insets show a 3D image and the 2D section of the central layer of the cell-populated hydrogel after 12 h. **d**, **e**, **f** Quantification of cells grown for 24 h on dried (**d**, **e**) or fresh (**f**) nanocomposite films. Cell numbers were determined by the CCK-8 assay and normalized to data obtained after adhesion for 4 h (red dashed line). The bars in **e** represent numbers of cells that remained on the substratum after adhesion for 1 h, thorough washing and subsequent cultivation for additional 4 h or 24 h (blue or gray bars, respectively, numbers on top indicate the percentage change). All data are represented by mean ± S.D. of triplicate samples.

It is well established that cell-material interactions are important guidelines for the development of materials for the control and use of cellular systems^41^. For example, the modulation of cell adhesion and proliferation by synthetic materials is crucial for tissue engineering and tissue replacement^42^. To investigate the utility of the nanocomposites for applications in in vitro cell culture, we first established that none of the various materials revealed cytotoxicity on MCF7 breast cancer cells (Supplementary Figs. 26–28). For the in-depth investigation of the composites, we prepared a library of the various materials inside the wells of 96-well glass bottom plates (Fig. 4a and Supplementary Fig. 29). Typically, polymerization of 75 μL reaction mixture for 48 h led to formation of a hydrogel layer of about 350 μm thickness. These freshly prepared materials were either used directly for cell culture experiments (discussed below) or else subjected to drying under vacuum at room temperature for 4 h (Fig. 4). As determined by confocal microscopy imaging, the drying led to formation of thin films of about 7 μm thickness, corresponding to an about 98% loss of volume upon drying. Imaging analysis also showed that neither the fresh (fully hydrated) nor the dried (condensed) state films swell upon immersion in buffer or cell culture medium. In accordance to the results obtained by SEM imaging (Supplementary Fig. 15), AFM measurements (Supplementary Fig. 16) indicated that the films’ surface topography displayed typical surface roughness (*R*a) values of about 20–30 nm, whereas standard tissue culture substrates (PLL, PLL + G, FN, Matrigel, Geltrex) are much smoother (*R*a < 3 nm). Furthermore, determination of the films’ hydrophobicity properties by contact angle measurements (Supplementary Fig. 30) indicated that S100, SC50, SC25, SC12.5 are superhydrophilic (*θ* < 5°, see Table 1) and the hydrophobicity slightly increased with increasing CNT ratio. However, even SC6.25, SC2.5, and C100 (*θ* ≈ 3–9°) are substantially more hydrophilic than PLL or PLL + G (*θ* ≈ 45° or 34°, respectively).

To investigate the interaction of cells with the dried composite films, we used MCF7 cells that constitutively express an eGFP-tagged epidermal growth factor receptor located on their cell membrane (MCF7_eGFP_). Previous studies had shown that these cells are well characterized, they enable direct microscopy observation of the cellular membrane and the overexpression of the eGFP-tagged EGFR has no negative effect on the cell behavior^43^. After seeding onto the hydrogels, cells were cultured for 24 h. Quantitative assessment of cell proliferation with the CCK-8 assay revealed distinctive material-dependent differences in the adhesion, proliferation and motility of the adhered cells (Fig. 4d–f).

The composition of the dried hydrogels had a marked effect on cellular proliferation. S100 and SC50 films induced proliferation rates that were significantly higher than on the standard tissue culture surfaces (PLL, FN). The other composite films also induced proliferation, however, with decreasing effectiveness at increasing amounts of CNT (Fig. 4d). Microscopic inspection revealed that MCF7 cells on these films showed a more pronounced elongated fusiform and flattened morphology than cells adhered on PLL surfaces (Fig. 4b and Supplementary Figs.  31 and 32), presumably due to the more effective formation of cell-substrate contact sites (Supplementary Fig. 33). Studies with rat embryonic fibroblast cells REF52 led to similar results (Supplementary Figs. 34–36), whereas control experiments with unpolymerised SiNP-P showed no comparable cell adhesion (Supplementary Fig. 37). Live-cell imaging suggested that cells on SC50 are more agile in terms of spreading, motility, and proliferation than cells on PLL surfaces (Supplementary Movies 1 and 2). The high attractiveness of dried SC50 was also suggested by experiments, in which cells were allowed to adhere to either PLL or SC50 surfaces (Supplementary Fig. 38) or to migrate from PLL to SC50 surfaces (Supplementary Fig. 39).

To analyze cellular adhesion strength in more detail, cells were allowed to adhere for 1 h on the various surfaces and the samples were then washed thoroughly to remove weakly adhered cells. As the number of remaining cells (blue bars in Fig. 4e) is proportional to adhesion strength, it was evident that cells adhere more strongly with increasing amounts of CNT in the composite materials. Indeed, a closer inspection of the data shows that cellular adhesion within SCx materials increases with increasing CNT content and thus with increasing hydrophobicity (Supplementary Fig. 40). While nano-roughness does not seem to play a decisive role within the series of SCx materials, this property may favor increased adhesion as compared to PLL (blue bars in Fig. 4e). Proliferation activity, however, showed the opposite effect (gray bars in Fig. 4d, e). There the maximum cell growth was observed for SC100 and SC50, while within the SCx series a clear trend is evident that growth decreases with increasing CNT content and thus increasing adhesion strength. PLL and FN surfaces are in the upper middle range.

These observations are in general agreement with previous results indicating that highest motility and proliferation occur at an intermediate adhesion strength, whereas strong adhesion impairs these cellular processes^44,45^. Furthermore, the preference of tumor cells to grow on rough surfaces^26,46,47^ and strongly adhere to CNT coatings^48,49^ has previously been observed. Since it is well established that cell behavior is influenced by a variety of factors in the matrix, such as matrix stiffness, elastic properties, its hydrophobicity and possible points of contact for extracellular protein domains^7,50,51^, we suggest the observed cell behavior as a specific response of the cells to the physical and mechanical properties of the nanocomposites. Of note, the cell growth on the fully hydrated, fresh SCx (Fig. 4f) differs from that on dried SCx materials, as the transmigration ability of the cells must also be taken into account (discussed below).

### Control of cellular transmigration, release, and expansion

Microscopy studies on the interaction of MCF7_eGFP_ cells with the fresh composite materials showed that cells rapidly transmigrate into the matrix of hydrogels S100 and SC50 with an apparent velocity of about 2  μm h^−1^ (Fig. 4c and Supplementary Figs. 41 and 42). In-depth investigation of proliferation showed that growth of cells on S100 and SC50 was slightly lower than on the PLL and FN surfaces. The other composite films even decreased proliferation upon increasing amounts of CNT (Fig. 4f), most likely due to the increased interaction strength that inhibits cell expansion, similar as observed for the dried composites (Fig. 4d, e).

**Fig. 5:**
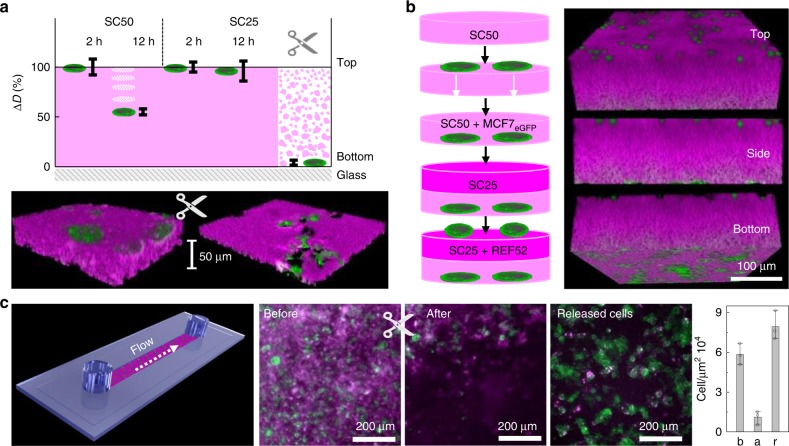
Control of cellular transmigration and release. **a** Vertical transmigration controlled by time, material composition and on-demand matrix degradation. Δ*D* (%) represents the relative distance between cells and the glass substrate. The images show MCF7_eGFP_ on fresh SC25 before and after endonuclease digestion. **b** Scheme (left) and representative fluorescence images (right) of a layered cell stack containing REF52 and MCF7_eGFP_ cells. **c** Flow-assisted capture and release of MCF7_eGFP_ in a microchannel coated with SC25 before and after endonuclease digestion and of the released cells. The bars show the average cell densities of the three stages.

We observed that cells could not transmigrate through but rather dug themselves into the upper layer of materials with higher contents of CNT, such as SC25 (Fig. 5a and Supplementary Figs. 43 and 44). Most likely, this effect is caused by the increased entanglement and mechanical stiffness of SC25 as compared to SC50 (*G*_0_ of 8.5 or 4.8 Pa, respectively, Table 1). Similar results were obtained with REF52 cells that revealed an even greater transmigration velocity of about 4.5 μm h^−1^ on SC50 (Supplementary Fig. 45). To demonstrate that cell transmigration control can be exploited for the engineering of cell architectures, we assembled layered cell stacks by allowing MCF7 cells to pass through an initial film of SC50 and subsequent sealing of this assembly by in situ polymerization of an SC25 layer. Adhesion of REF52 cells then led to the formation of stacked cell layers separated by an ~120 μm thick hydrogel matrix (Fig. 5b).

This example clearly shows how the transmigration of cells can be controlled by adjusting the fraction of CNT in the composites. This approach paves the way to the production of various artificial 3D architectures of cells. Such arrangements are useful as artificial models to study fundamental phenomena like epithelial-to-mesenchymal transition (EMT) processes, long-distance cell-cell communication or as functional constructs for toxicology research^52^. An equally highly topical field in biomedical research is the use of microfluidic systems for cell culture, for example, to carry out perfusion cultures to mimic blood vessels and tissue conditions or to achieve cell adhesion and release under dynamic conditions and to facilitate cell recovery^53^. Owing to their adjustable adhesion properties and their easy degradability, SC materials should be advantageous for such applications.

We thus tested whether SC25 can be used for selective capture and enzyme-triggered release of surface-bound cells. Indeed, treatment of the SC25-bound cells with a restriction enzyme for 2 h led to reduction of the gel’s thickness from 45 to 15 μm (Fig. 5a). The released cells transmigrated into the broken nanocomposite matrix towards the underlying glass surface where they propagated to form small cell populations 10 h after enzymatic release (Fig. 5a and Supplementary Figs.  43 and 44). We then use this controllable cell-material interaction for cell adhesion and release studies under flow conditions to illustrate the utility of the SC materials for the development of improved artificial systems for cell culture^54^. For this purpose, the bottom of a microchannel was coated with SC25 (Fig. 5c). Using a microfluidic system (Supplementary Fig. 46), transfusion of the channel with a suspension of MCF7_eGFP_ cells led to formation of surface-bound cell populations after 2 h. The SC25 coating was then broken by addition of BstEII-HF restriction enzyme (0.5 h) and the collected outflow of the channel was cultured for an additional 24 h under standard conditions in a petri dish. Fluorescence microscopy analysis clearly showed that the cells had not been harmed by the procedure but were capable of adhesion, spreading, and proliferation after release from the channel (Fig. 5c and Supplementary Figs. 47 and 48 and Supplementary Movie 3). These results underline the utility of the nanocomposite materials for biomedical research.

**Fig. 6:**
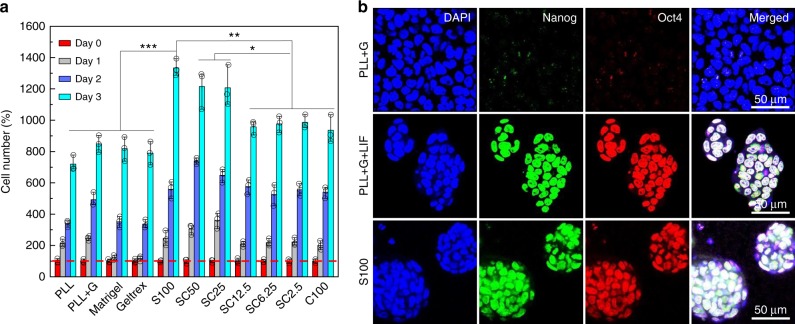
Stem cell cultivation and maintenance of mESC stemness. **a** Quantification of time-dependent mESC growth on various substrata, normalized to data obtained from mESC on PLL for 4 h (red dashed line). All data are represented by mean ± S.D. of triplicate samples. **b** Representative fluorescence images of immunostained mESCs grown for 4 days on gelatine-coated PLL in the absence (upper row) or presence (middle) of LIF or on S100 in the absence of LIF (bottom).

To further substantiate the usefulness of the SC materials, we investigated their suitability for expansion of stem cells and the maintenance of their stemness. These features are considered a critical step towards the development of stem cell-based therapies^55^. In general, the culturing of stem cells on feeder cell layers or the use of complex and quite undefined protein mixtures like matrigel^56^, often in the presence of supplements, such as leukemia inhibitory factor (LIF)^57^, are still the gold standard for maintaining pluripotency of stem cells. However, these protocols are difficult to implement for routine use, since batch-dependent changes in the materials obtained from biological sources can lead to strong fluctuations in quality. For the development of matrices that can be produced under GMP- and GLP-compliant standards, it would therefore be desirable to have standardized protocols based exclusively on clearly defined artificial components. Towards this end, we compared the cultivation of mouse embryonic stem cells (mESCs) on standard tissue culture substrates (PLL, gelatine-coated PLL, Matrigel and Geltrex) in the presence or absence of LIF, with the fresh composite materials in the absence of LIF (Fig. 6 and Supplementary Figs. 49–55).

Quantification of growth rates by microscopic analysis and CCK-8 assays (Fig. 6a and Supplementary Figs. 49 and 50) consistently showed that the mESCs proliferated faster on the composite materials than on the control surfaces, with SC100, SC50, and SC25 showing the best performance. The time-dependent consideration showed that mESC growth proceeds faster on more rigid surfaces in the beginning (propagation rates: SC25 > SC50 > S100 at day 1), whereas the growing 3D colonies expand better in the softer matrices with increased culture times (propagation rates: SC50 > SC25 > S100 or S100 > SC50 > SC25 at days 2 or 3, respectively). Importantly, the spheroid colonies grown on the composites maintained their pluripotency, as indicated by immunostaining of the pluripotency markers Nanog, Oct4, and Sox2, whereas LIF was required for the control surfaces (Fig. 6b and Supplementary Figs. 51–53). These observations are consistent with previous studies on soft substrata for preserving stemness^55,58^. Furthermore, comparative studies on the growth of mESC spheroids under two-dimensional (2D) or 3D culture conditions indicated that growth in S100 led to spheroids that reveal the desired uniform, concentric, compact and raised shape to a much greater extent than those grown on standard substrates (Supplementary Fig. 54).

**Fig. 7:**
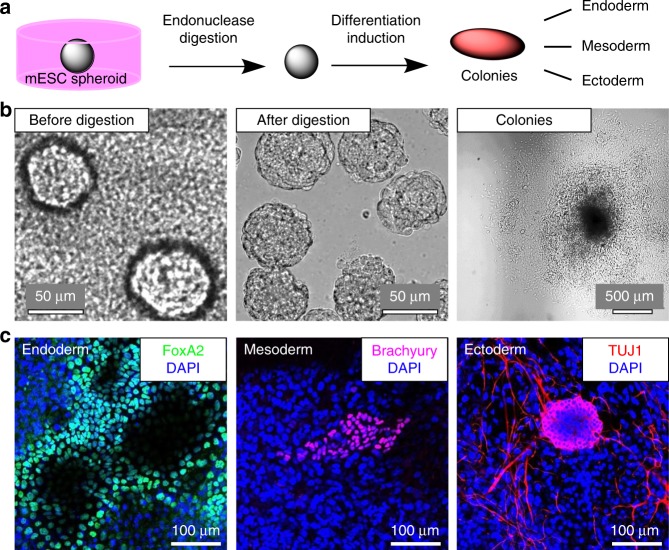
Release and differentiation of mESC spheroids. **a** Schematics of mESC culturing, release of spheroids, and expression of germ layer markers in replated aggregates. **b** Bright-field images of mESC spheroids before and after release from S100 culture materials and formation of differentiating colonies, observed via the outgrowth of cells in the periphery. **c** Representative fluorescence images of immunostained markers for germ layers in the colonies after 14 days.

To further explore their applicability for stem cell research, we used the nanocomposite materials for the growth and isolation of spheroids (Fig. 7). To this end, mESC were seeded directly onto S100 and were allowed to grow for 4 days. The subsequent enzymatic release of these spheroids from S100 led to almost monodisperse cell bodies, which showed the desired shape to a much greater extent than those grown by established protocols based on Matrigel, Geltrex or pNIPAM substrates (Supplementary Figs. 54–56). The mESC spheroids obtained from S100 cultivation and release could be further processed using standard methods for cultivation and differentiation of embryoid bodies (EBs) (Fig. 7b). Cellular migration and outgrowth from the differentiating aggregates occurred in a similar way as observed for EBs prepared by the established hanging drop method^59^ (Supplementary Fig. 57). As determined by immunostaining, the resulting colonies exhibited the expected markers of the differentiated germ layers (Fig. 7c). Overall, the results suggest that the composites can open the door to simpler and more robust stem cell culture techniques. Such methods are urgently needed for basic research on pluripotency and differentiation mechanisms of stem cells, as well as for applications in regenerative medicine and stem cell-based therapies^60^.

## Discussion

In conclusion, we developed a class of nanocomposite DNA materials that can be customized for cell culture applications by means of a series of set screws. Since the materials are readily obtained from DNA-modified nanoparticles through enzymatic biosynthesis, our synthetic approach is generic and applicable to a wide range of nanomaterials, whose composition can be gradually tuned at will. As demonstrated by variation of the SiNP:CNT mass ratio, this can lead to non-linear effects on the materials’ physicochemical properties, such as mechanical stiffness and viscosity, which are fundamental for the establishment, maintenance and control of cellular systems. Our exemplary demonstration that cellular adhesion, propagation and motility can be controlled by adjustment of SiNP:CNT particle composition suggests that incorporation of particles from other materials and/or different size, aspect ratio, or binding valency will lead to an almost unlimited variety of designer materials. Importantly, our synthetic approach is amenable to automated synthesis (Supplementary Fig. 29) as a means to create and screen libraries of materials for unpredictable properties that can arise from distinctive compositions of building blocks. As initial demonstration, we took advantage of this combinatorial approach by establishing substrata for control of cellular adhesion and transmigration through the hydrogel matrix. The composite materials could also be applied to enhance stem cell proliferation with concomitant preservation of their stemness to enable less complex cultivation procedures. Based on these measures, the composites clearly outperform conventional tissue culture substrata.

Another important implication of this work arises from the fact that the materials display not only the features emerging from those of the crosslinked nanoparticles, such as cell attractiveness of SiNP and stiffness of CNT particles harnessed here. However, distinct bioinstructive properties can be conveniently implemented into the materials through rational engineering of the connecting DNA polymer backbone. The here demonstrated incorporation of enzymatic restriction sites enabled the control of vertical transmigration, as well as the on-demand release of captured cells. These initial examples of “functionality by design” suggest that implementation of basic concepts of DNA nanotechnology can open the door to even more sophisticated materials that enable control of cell response.

## Methods

### Materials

Tetraethyl orthosilicate (TEOS), 3-(trihydroxysilyl)propyl methylphosphonate (THPMP, monosodium salt solution), (3-aminopropyl)trimethoxysilane (APTMS), poly(ethylene glycol) methyl ether maleimide (mPEG-mal, molecular weight of ~2000), N1-(3-trimethoxysilylpropyl)diethylenetriamine (DETAPTMS), tris(2-carboxyethyl)phosphine hydrochloride (TCEP, 0.5 M in water), sodium cyanoborohydride, single-walled carbon nanotubes (CNT, 0.83 nm average diameter, 1 μm median length), oligonucleotides, β-mercaptoethanol, poly-l-lysine (molecular weight of 30,000–70,000), gelatine, G418 disulfate salt solution (50 mg mL^−1^ in water), FITC-dextran (70 kDa) and Cell Counting Kit-8 (CCK-8) were purchased from Sigma-Aldrich. Cyclohexane, 1-hexanol, glutaradehyde (50% in water) and Matrigel® were from VWR. Fluorescamine and (3-mercaptopropyl) trimethoxysilane (MPTMS) were purchased from Alfa Aesar. T4 DNA ligase (400,000 U mL^−1^), phi29 DNA polymerase (10,000 U mL^−1^), restriction enzymes (BstEII-HF and SexAI), and deoxynucleotide (dNTP) solution mix (10 mM for each nucleotide) were from New England Biolabs. Sulfo-cyanine 5 NHS-ester was from Lumiprobe. Triton X-100, 4’,6-diamidino-2-phenylindole (DAPI), and glycine were from AppliChem. Trypsin/EDTA solution (0.25%/0.02%) and fetal bovine serum (FBS) were from Biochrom. Eagle’s minimum essential medium (EMEM), Dulbecco’s modified Eagle’s medium (DMEM, high glucose), and l-glutamine were from Gibco Laboratories. Pansera ES FBS was obtained from PAN-Biotech. Fluorescein-12-dUTP solution (FITC-dUTP, 1 mM), penicillin/streptomycin, ethidium bromide (EtBr, 10 mg mL^−1^ in water), SYBR™ Gold Nucleic Acid Gel Stain (10,000 × concentrate in water), SYBR™ Green I Nucleic Acid Gel Stain (10,000 × concentrate in DMSO), Alexa Fluor^®^488 phalloidin, CellTracker™ Green CMFDA (5-chloromethylfluorescein diacetate), Calcein AM, Geltrex™ and Dulbecco’s phosphate-buffered saline (DPBS) for cell experiments were from Thermo Fisher Scientific. Paraformaldehyde (PFA, 16% aqueous solution) was from Polysciences. ESGRO^®^ recombinant mouse lukemia inhibitory factor (LIF) protein (1 × 10^6^ U mL^−1^) was from Merck. All chemicals were used as received without further purification.

### UV/Vis and fluorescence spectroscopy

Absorption spectra were recorded using an Agilent Cary 100 UV–Vis spectrophotometer or a BioTek Synergy H1 microplate reader at room temperature. The latter was also used to record fluorescence spectra. For optical density measurements (Supplementary Fig. 8b), 50 μL of RCA reaction mixture was placed in wells of glass bottom 96-well plates (MoBiTec GmbH), incubated at 30 °C and the optical density was measured every hour using a BioTek Synergy H1 microplate reader.

### Transmission electron microscopy (TEM) analysis

The size and morphology of SiNP were investigated using a FEI Titan³ 80–300 electron microscope (FEI Company) at an accelerating voltage of 200 kV and the resulting images were analyzed with ImageJ software (http://www.rsb.info.nih.gov/ij/download.html)^61^. Furthermore, STEM and EDS analysis were performed using the same instrument at the same voltage, but equipped with a high-angle annular dark-field (HAADF) detector (Fischione Company) and a high solid-angle silicon drift detector system (FEI Company). The samples were prepared by placing a drop of the sample solution onto a 200-mesh carbon-coated Cu grid (Plano GmbH), which was then air-dried at room temperature.

### Dynamic light scattering (DLS) measurements

The kinetic hydrodynamic size of SiNP during RCA reaction was studied by DLS at 30 °C using a Malvern Zetasizer Nano ZSP equipped with a standard 633 nm laser.

### Fluorescence imaging analysis

Fluorescence micrographs of the materials and cell lines were recorded either by an Axiovert 200 M, an Axio Observer, or an LSM 880 (Carl Zeiss).

### Scanning electron microscopy (SEM) analysis

After lyophilization or vacuum drying, the SiNP/CNT-DNA nanocomposite materials were coated with 4 nm of platinum using ion beam deposition and their morphology was characterized using a QUANTA 650-FEG scanning electron microscope (FEI Company) with an accelerating voltage of 5–10 kV.

### Raman analysis

Raman analysis was performed using a Senterra Raman microscope (Bruker Optics) equipped with a 532 nm laser operated at 5 mW output power. An Olympus MPLAN 100 × objective, N.A. 0.9 was used for visualization of the samples, focusing of the excitation beam, and collimation of the backscattered light as well. The beam spot diameter on the sample was 5 µm. The measurement time was 15 s with three coadditions (3 × 5 s) for each spot. Samples were prepared by transferring a piece of SiNP/CNT-DNA nanocomposite (~ 5 μL) onto a flat gold-coated Si wafer and air-drying before measurements.

### Multiple particle tracking (MPT) method

The viscoelasticity of hydrogels was investigated via Multiple Particle Tracking (MPT) according to previous work^38^. Briefly, MPT experiments were performed using an inverted fluorescence microscope (Axio Observer D1, Zeiss), equipped with a Fluar 100 × , N.A. 1.3, oil-immersion lens combined with a 1 × optovar magnification changer. The Brownian motion of the tracer particles, green fluorescent polystyrene microspheres of 0.2, 0.5, and 1.0 μm diameter (Bangs Laboratories), was tracked. Prior to the measurement, the tracer particles were trapped inside hydrogels by adding tracer particles to the initial RCA reaction mixture. Images of these fluorescent beads were recorded with a sCMOS camera Zyla X (Andor Technology: 21.8 mm diagonal sCMOS Sensor size, 2160 × 2160 square pixels) and the displacements of the particle centers were monitored in an 127 × 127 μm field of view at a rate of 50 fps. Movies of the fluctuating microspheres were analyzed by a custom MPT routine incorporated into the software Image Processing System (Visiometrics iPS) and a self-written Matlab program^62^ based on the widely used Crocker and Grier tracking algorithm^63^.

### Fluorescence recovery after photobleaching (FRAP)

To determine the diffusion coefficients of solutes inside the SiNP/CNT-DNA composite materials, FRAP measurements were performed by laser scanning confocal microscopy (LSM 880, Zeiss). To this end, the composite materials were immersed in a solution containing 1 kDa FTIC-dUTP (40 μg mL^−1^ in PBS, 12 h) or 70 kDa FITC-dextran (500 μg mL^−1^ in PBS, 12 h) to ensure homogeneous soaking. After determining the location of interest, time-series of images with a resolution of 512 × 512 pixel were recorded using a highly attenuated laser beam (1% transmission). The interval time between two consecutive images was 2 s for FITC-dextran in composite materials and conventional gels (i.e., Matrigel, Geltrex) and 0.5 s for FITC-dextran in PBS and RCA mixture and FTIC-dUTP in all samples, respectively. After the acquisition of five prebleach images, a 50 μm diameter spot was bleached at maximum laser intensity (100% transmission). Immediately after bleaching, a stack of 225 images was acquired at low laser intensity (1% transmission) to monitor the recovery of fluorescence inside the bleached area. Data processing was achieved by Zeiss software, as detailed in Supplementary Fig. 10.

### Atomic force microscopy (AFM) analysis

The morphology of oligonucleotide P2-functionalized CNT (CNT-P) was observed by AFM. Briefly, the sample was diluted up to 25-fold in TE-Mg^2+^ (20 mM Tris base, 1 mM EDTA, 12.5 mM MgCl_2_, pH 7.4). Five microliters of the resulting solution was deposited onto a freshly cleaved mica surface (Plano GmbH) and adsorbed for 3 min at room temperature. After addition of 10 µL TAE-Mg^2+^ (40 mM Tris, 20 mM acetic acid, 2 mM EDTA, 12.5 mM Mg acetate, pH 8.0), the sample was scanned with sharpened pyramidal tips (SNL-10 tips, radius 2 nm, spring constant 0.35 N m^−1^, Bruker) using a MultiModeTM 8 microscope (Bruker) equipped with a Nanoscope V controller in Tapping Mode.

To analyze the morphology and roughness of SiNP/CNT-DNA nanocomposite surfaces, the fresh materials were rinsed with distilled water, transferred to the petri dish and dried as described above. The dried samples were immersed in water and imaged with pyramidal tips (ScanAsyst Fluid tips, radius 20 nm, spring constant 0.7 N m^−1^, Bruker) using a NanoWizard 3 atomic force microscope (JPK) under a force-curve based imaging mode (QI^TM^). Images were acquired with 256 × 256 pixel resolution and the roughness (Ra) of the surfaces was extracted using the JPK data processing software (version spm-6.0.74). PLL, PLL + G, Matrigel, and Geltrex control surfaces were characterized in the same way.

### Static contact angle measurements

Water contact angle (WCA) values were determined by the sessile drop method. Briefly, 1 µL of double distilled water was placed on the PLL, PLL + G, or SiNP/CNT-DNA nanocomposite surfaces at room temperature. Subsequent to incubation for 30 s, a photographic image of the drop was recorded with a Stingray F-033 camera. Image analysis was carried out with drop shape analysis (DSA) software (DSA Version 1.90.0.14).

### Electrophoresis

Samples were loaded onto a 6% native polyacrylamide gel (1 × TAE-Mg^2+^), run using a voltage of 120 V for 45 min and subsequently stained with SYBR Gold (Supplementary Fig. 5a). DNA samples analyzed with a 2.5% agarose gel (1 × TAE-Mg^2+^) were run with a voltage of 120 V for 45 min and subsequently stained with SYBR Gold (Supplementary Fig. 5b) or stained with EtBr (Supplementary Figs. 6 and 20a).

### Melting curve analysis

The purified S100 was stained with the SYBY Green I (1x TAE) for 1 h and its melting curve was subsequently measured in the real-time PCR thermocycler (Corbett research). Fluorescence signals during the melting of the S100 were monitored in the green spectral channel using 0.5 °C steps with a hold of 5 s at each step from 50 °C to 99 °C.

### Circular dichroism (CD) spectroscopy

CD spectra of the DNA hydrogel backbone were recorded on a Jasco-815 circular dichroism (CD) spectro-polarimeter. Prior to measurement, the samples were placed into rectangular quartz glass cuvettes (Hellma GmbH & Co. KG), with 0.1 cm optical path length, maintained at 25 °C or 90 °C in a water thermostat. The instrument was flushed with N2 gas before recording spectra in order to remove O_2_ from the lamp housing and the sample compartment so as to prevent ozone formation and to minimize damage to the optical system.

### Synthesis of multifunctional silica nanoparticles

Multifunctional SiNP (SiNP-1) with amino, thiol and phosphonate groups and an average particle size of 80 nm were synthesized according to previous work (Supplementary Fig. 1)^34^. Typically, cyclohexane (38 mL), 1-hexanol (9 mL), and triton X-100 (9 mL) were mixed vigorously in a 250 mL round-bottom glass bottle. Double distilled water (2 mL) was added to the mixture to produce stable reverse micelles. After mixing for 10 min, TEOS (500 μL) was added to the mixture followed by addition ammonia solution (28–30%, 500 μL). This mixture was stirred at room temperature for 24 h. Subsequently, additional TEOS (250 μL) was added to the mixture, and after stirring for 30 min, THPMP (200 μL) and DETAPTMS (50 μL) were added to modify the surface of the nanoparticles with negatively charged phosphonate and amino groups. The mixture was allowed to react for 24 h and subsequently MPTMS (30 μL) was added to modify the nanoparticle’s surface with thiol groups. The mixture was stirred at room temperature for an additional 3 h. The micelles were broken with acetone, and the resulting nanoparticles were centrifuged and washed at least five times with absolute ethanol, and finally dispersed in PBS buffer (23 mM KH_2_PO_4_, 77 mM K_2_HPO_4_, 50 mM NaCl, pH 7.4) to a final concentration of 10 mg mL^−1^.

The preparation of Cy5-doped SiNP-1 was carried out according to reported procedures^26^. In brief, 1.3 μmol sulfo-Cy5-NHS was dissolved in 1 mL of anhydrous DMSO and APTMS was added at a molar ratio of 10:1 APTMS:dye. The mixture was allowed to react at room temperature for 12 h. Subsequently, the crude reaction mixture (200 μL) was transferred into a 250 mL round-bottom glass bottle containing stable reverse micelles prepared as described above. Subsequently, Cy5-doped SiNP (Cy5@SiNP-1) formed in the dark after further addition of TEOS, THPMP, DETAPTMS, and MPTMS in the presence of ammonia solution according using the same procedure described above for preparation of SiNP-1.

### Immobilization of PEG and ssDNA on silica nanoparticles

To install PEG groups on the surface of SiNP-1, TCEP (0.5 M solution, 8.0 μL) was added to 1 mL PBS solution of SiNP-1 (10 mg mL^−1^) to reduce any disulfide bonds. Subsequently, a DMSO solution of mPEG-mal (50 mg mL^−1^, 10 μL) was added to the mixture. After incubation at room temperature overnight, the modified nanoparticles were purified by centrifugation and re-dispersion with PBS buffer for 3–5 times. The resulting nanoparticles are denoted as SiNP-2.

Next, amino-modified P1 primer (aP1) was covalently immobilized on the particle surface via glutaraldehyde coupling. Typically, SiNP-2 (10 mg mL^−1^, 1.0 mL) in PBS buffer were mixed with glutaraldehyde (50% in water, 250 μL), and the mixture was stirred at room temperature for 1 h. The resulting nanoparticles were washed three times with PBS buffer, re-dispersed in PBS buffer (1.0 mL) and mixed with aP1 (100 μM, 50 μL). The mixture was incubated at room temperature for 12 h. Subsequently, glycine (0.4 M, 1.0 mL) was added to block any unreacted aldehyde groups, followed by addition of sodium cyanoborohydride (60 mM, 400 μL) to reduce Schiff’ bases into stable secondary amines. The P1 primer-modified SiNP are denoted as SiNP-P. As a control, the SiNP-M1 were also synthesized using the same protocol.

### Ligation of RCA template on SiNP-P

The linear ssDNA (T) phosphorylated at the 5ʹ end was circularized through hybridization with P1 attached on the surface of SiNP-P using T4 DNA ligase. To this end, linear ssDNA (T, 10 µM, 30 µL) and 10 × T4 DNA ligation buffer (500 mM Tris-HCl, 100 mM MgCl_2_, 10 mM ATP, 100 mM dithiothreitol (DTT), 7.5 µL) were added to 60 μL SiNP-P suspension (10 mg mL^−1^), and the mixture was incubated at 25 °C for 3 h. After addition of 2.5 µL T4 DNA ligase (400,000 U mL^−1^), the mixture was further incubated for more than 3 h at 25 °C to ligate the nicked ends of the template, leading to the formation of particle-primer-template (SiNP-P-T) complexes.

### RCA polymerization of SiNP-P-T

The RCA reaction mixture contained dNTPs (10 mM, 10 µL), 1 × BSA (1 mg mL^−1^, 5 µL), 10 × phi29 DNA polymerase buffer (500 mM Tris-HCl, 100 mM MgCl_2_, 100 mM (NH_4_)_2_SO_4_, 40 mM DTT, pH 7.5, 5 µL) and phi29 DNA polymerase (10,000 U mL^−1^, 5 µL). The polymerization was initiated via the addition of 50 µL of the SiNP-P-T particles. After incubation at 30 °C for various reaction times, the SiNP-DNA hydrogels were purified by carefully replacing the reaction mixture with DPBS for 5–7 times and the SiNP-DNA hydrogels were collected and stored at 4 °C before use. With a final SiNP-P concentration of 4 mg mL^−1^, the SiNP-DNA hydrogels (S100) generated by RCA for x h were denoted as S100_xh_, as indicated in Supplementary Table 3.

### DNA oligonucleotide-assisted solubilization of CNT

Four-hundred twenty-eight microliters of an aqueous dispersion containing 1.2 mg single-walled carbon nanotubes (CNT) were mixed with 344 µL aqueous solution of ssDNA oligonucleotide (P2, 100 µM) and 428 µL aqueous solution of NaCl (0.28 µM), followed by ultrasonication on ice for 90 min at a power of approx. 10 W using a Ultrasonic Cleaner (VWR). The resulting products were centrifuged at 16,000 × *g* and 4 °C for 90 min to remove CNT aggregates. The free DNA was removed by ultrafiltration at 4000 × g and 4 °C for 10 min using an ultrafiltration unit Vivaspin 6 with a molecular weight cut-off (MWCO) of 50 kD (Sartorius Stedim Biotech), and the P2 modified CNT (CNT-P) were re-dispersed from the filtration membrane using distilled water. The purification process was repeated several times until no free DNA could be detected in the flow through. The concentration of CNT in CNT-P was determined by absorbance at 664 nm using a calibration curve obtained from standards of known concentrations of sodium dodecyl sulfate (SDS)-dispersed CNT. For control purposes, CNT-M2 was synthesized using the same protocol.

### Ligation of RCA template on CNT-P for RCA polymerization

The hybridization and ligation of the RCA template (T) on CNT-P was performed as described above for SiNP-P. Typically, 30 µL of linear ssDNA (T, 10 µM) and 60 µL of CNT-P (variable concentrations according to Supplementary Table 5) were used. The resulting particles CNT-P-T were used for RCA polymerization. RCA reaction mixture was prepared as described above, and the reaction was initiated by addition of 50 µL of the CNT-P-T (variable concentrations according to Supplementary Table 5). After incubation for 48 h, the aqueous phase was carefully removed from the CNT-DNA hydrogels (Cx) and the materials were washed 5–7 times with DPBS.

### Synthesis of ternary composite materials SCx

Mixtures containing SiNP-P and CNT-P in variable amounts (Supplementary Table 1) were subjected to ligation with RCA template (T) and subsequent RCA polymerization, similar as described above. After incubation for 48 h, SiNP/CNT-DNA composite hydrogel (SCx) were carefully washed and used for cell culture either fresh or after drying in vacuum at room temperature for 4 h.

### Surface coating procedures

Poly-l-lysine (PLL, MW of 30,000–70,000) was dissolved in 0.15 M borate buffer (pH = 8.3) with a concentration of 0.5 mg mL^−1^ and sterilized by filtration. Cover glasses (MoBiTec GmbH) were immersed in the PLL solution for 12 h and subsequently rinsed with water for three times. For gelatin coating, PLL-coated glasses were immersed into gelatin solution (100 μg mL^−1^, in water) for 12 h and subsequently rinsed with water twice. Fibronectin (FN)-coated cover glass was prepared by covering the glass surface with FN solution (10 μg mL^−1^, in water) at 37 °C. After at least 30 min, the excess FN solution was removed. Matrigel (Corning) or Geltrex (Thermo Fisher Scientific) coating of glass surfaces was achieved by polymerization of diluted gel solutions, according to manufacturer’s instruction. To this end, the as-obtained gel solution was diluted with cold PBS for ten times on ice. Fifty microliters of this solution were placed on the cover glass and allowed to polymerize for 2 h at 37 °C. The coated surface was washed with water twice. Surfaces coated with pNIPAM were prepared according to previous work^64^. All coated surfaces were air-dried for 2 h at room temperature prior to use.

### Cell culture

Human MCF7_eGFP_ breast cancer cells stably transfected to express the EGF receptor fused to the enhanced green fluorescent protein (eGFP-EGFR) were obtained as a gift from the Max-Planck Institute for Molecular Physiology (Dortmund). The cells were cultured in 25 cm^2^ tissue culture flask (Corning Inc.) with MCF7_eGFP_ medium, comprises EMEM, with addition of 1% penicillin/streptomycin, 10% FBS and 0.6% G418 disulfate salt solution at 37 °C in a 5% CO_2_ environment. The cells were washed twice with DPBS (-/-) (without calcium and magnesium) and trypsinated by addition of 500 µL 0.25% Trypsin solution in PBS-EDTA (PBS with 0.02% EDTA) for 3 min. The trypsin activity was blocked by addition of 4.5 mL of fresh MCF7_eGFP_ medium. The cell concentration of the resulting suspension was determined by hemocytometer analysis. Native MCF7 cells (ATCC) were also cultivated in EMEM with 1% penicillin/streptomycin, 10% FBS in the absence of G418 disulfate salt. Rat fibroblast cell line REF52 (University of Heidelberg) was cultured in DMEM supplemented with 1% penicillin/streptomycin, 10% FBS, and 2 mM l-glutamine. The mouse embryonic stem cells (mESCs), EB5 (129/Ola), (RIKEN BRC) were cultured in DMEM supplemented with 1% penicillin/streptomycin, 15% Pansera ES (Pan-Biotech), 2 mM l-glutamine, 0.1 mM β-mercaptoethanol, and 2000 U mL^−1^ LIF under standard culture conditions (37 °C and 5% CO_2_). The cells were passaged every 2 to 3 days.

### Cytotoxicity assay

CCK-8 cell viability assay was used to evaluate the cytotoxicity of the various composite materials according to manufacturer’s instructions. In brief, MCF7 cells or REF52 cells in 200 μL medium at a density of 8 × 10^3^ cells per well were seeded into a 96-well plate. After incubation at 37 °C and 5% CO_2_ for 12 h, the adherent cells were incubated with 200 μL fresh medium containing the various composite materials or PBS as control. After another 24 h incubation, 20 μL CCK-8 was added into each well, and the cells were then incubated for an additional 4 h at 37 ^o^C and 5% CO_2_. Subsequently, the absorbance at 450 nm was recorded with a BioTek Synergy microplate reader. Cell viability was calculated according to equation:

1 $${\mathrm{Cell}}\;{\mathrm{viability}} = \frac{{{\mathrm{A}}1 - {\mathrm{B}}}}{{{\mathrm{A}}0 - {\mathrm{B}}}}$$

where A0 and A1 represent the OD values of CCK-8 in medium containing cells after treatment with PBS or nanocomposites, respectively. B represents the OD value of CCK-8 in medium. Average number and standard deviation (S.D.) were determined from triplicates of each sample.

### Cell adhesion, spreading, and proliferation on materials

Materials were prepared as described in 75 μL RCA reaction mixture with different SiNP-P/CNT-P concentration ratios directly inside the wells of a glass bottom 96-well plate (MoBiTec GmbH). The materials were washed with distilled water and condensed by drying in vacuum (Eppendorf Concentrator plus, 20 mbar) at room temperature for 4 h. Subsequently, 6 × 10^3^ MCF7_eGFP_, MCF7 or REF52 cells per well in 200 μL medium were seeded on top of the hydrogels. After cultivation for 24 h, the cell proliferation was analyzed using the CCK-8 assay. To compare the adhesion strength of cells on the different composite surfaces, MCF7 cells were allowed to adhere for 1 h and then washed three times with DPBS. The number of the remaining cells was quantified with the CCK-8 assay. Furthermore, live-cell imaging of MCF7_eGFP_ cells was performed using confocal fluorescence microscopy for up to 24 h post seeding on standard tissue culture (PLL) and SC50, respectively (Supplementary Movies 1 and 2). The time interval between two frames was set at 36 min.

### Competitive adhesion assay

To compare the attractiveness of SC50 and PLL surfaces, a sample of SC50 was deposited and dried on a petri dish with PLL surface such that the hydrogel patch partially covered the petri dish surface. Subsequently, 1 mL medium containing 1.2 × 10^4^ MCF7_eGFP_ cells was transferred into petri dish to fully cover the entire surface of the petri dish. After another 24 h of cultivation, MCF7_eGFP_ cells were visualized by fluorescence microscope.

### Lateral cell-migration assay

A petri dish with a patch of SC50 was prepared as described above. Twenty microliters of medium containing 1.2 × 10^4^ MCF7_eGFP_ cells were transferred into the petri dish as a droplet in a distance of ~0.8 cm apart from the hydrogel. After 4 h incubation, the suspension cells were carefully removed by replacing the remaining droplet with 1 mL fresh medium. The adherent cells were cultured for 14 days and imaged at variable time points to monitor proliferation and lateral migration.

### Cell transmigration inside the SCx materials

The various nanocomposite materials were synthesized in a 25 μL RCA reaction inside the wells of a glass bottom microplate as described above. After careful washing of the resulting hydrogels with DPBS (3–5 times) and medium (3–5 times), 6 × 10^3^ MCF7_eGFP_ cells per well in 200 μL medium were seeded on top of the hydrogels and allowed to settle for 2 h. The cell behavior was monitored by confocal fluorescence microscopy using 3D *z*-stack and time lapse analyses. After cultivation of the cells for 24 h, the cell proliferation was analyzed with the CCK-8 assay and confocal fluorescence microscopy.

### Layered cell stacks

To illustrate the feasibility of nanocomposite-based fabrication of stacked cell layers, MCF7_eGFP_ and REF52 cells were chosen for the lower and upper cell layer, respectively. To initially prepare the lower layer, 35 μL SC50 reaction mixture were pipetted into a glass bottomed 96-well plate, allowed to polymerize for 48 h at 30 ^o^C, and washed by exchanging the reaction buffer with MCF7_eGFP_ medium. Subsequently, 6 × 10^3^ MCF7_eGFP_ cells were seeded onto the SC50 and cultured for 12 h. Then, the medium was replaced with fresh 10% glycerol/200 nM G-actin-contained MCF7_eGFP_ medium and the MCF7_eGFP_ cells-laden SC50 was covered with 35 μL of SC25 reaction mixture (without DTT in the reaction buffer). After polymerization at 30 ^o^C and 5% CO_2_ environment for another 12 h, the second layer of SC25 was washed by exchanging the polymerization mixture with REF52 medium. Then, 6 × 10^3^ REF52 cells pre-stained with CellTracker^TM^ Green (25 μM, 30 min) were seeded onto the top of SC25 layer, cultured for 12 h and subjected to microscopy analysis.

### Controlled cell release from SC25

SC25 was synthesized in a 25 μL RCA reaction inside the wells of a glass bottom microplate, as described above. In all, 6 × 10^3^ MCF7_eGFP_ cells per well in 200 μL medium were seeded on top of the hydrogels and allowed to adhere to the hydrogel surface for 12 h. Following, medium containing reaction buffer (50 mM potassium acetate, 20 mM tris-acetate, 10 mM magnesium acetate, 100 μg mL^−1^ BSA, pH 7.4) and BstEII-HF in a final concentration of 2 U mL^−1^ was added and the cells were monitored by confocal fluorescence microscopy.

### Cell release from SC25-coated microfluidic channels

Microfluidic channels (μ-Slide I Luer) were purchased from Ibidi. The channel length, width, and height are 50 mm, 5 mm, and 400 μm, respectively. To prepare a thin SC25 film inside the microfluidic channel, 100 μL RCA reaction mixture was pipetted into the channel and then the inlet and outlet of the channel were tightly sealed. Following to incubation at 30 ^o^C for 48 h, SC25-coated microchannel was carefully washed with distilled water and evaporated overnight in a sterile environment under normal pressure at room temperature. For fluidic cell experiments, the chip was connected with tubing to a peristaltic pump (IPC, Ismatec) and flushed with sterile DPBS for 30 min at a flow rate of 800 μL min^−1^. Subsequently, a MCF7_eGFP_ cell suspension (5 × 10^5^ cells mL^−1^ cell culture medium) was transfused through the microfluidic channel with a flow rate of 57.4 μL min^−1^ for 5 min. The flow was stopped for 2 h to allow the cells to settle and adhere to the SC25-coated surface. Then, medium without cells was transfused through the channel at a flow rate of 57.4 μL min^−1^ for 2.5 h. This process was monitored by fluorescence microscopy capturing images of the MCF7_eGFP_ cells every 10 min. Following, the channel was transfused with the above specified medium containing BstEII-HF for 30 min. The enzyme solution was replaced by fresh cell culture medium and the flow was continued for another 2.5 h. About 9 mL outflow volume were collected and the cells in the collected outflow were precipitated via centrifugation, resuspended in 200 μL fresh medium, and cultured in a 96-well plate with a cover glass bottom. After 24 h, the cells were examined by fluorescence microscopy.

### Proliferation of mESCs

To monitor the mESC proliferation on fresh composite materials, 5 × 10^3^ cells were seeded on the freshly prepared SCx (S100, SC50, SC25, SC12.5, SC6.25, SC2.5, and C100, respectively) materials. The diameters of the colonies were constantly measured for up to 3 days (Supplementary Fig. 50). Alternatively, the relative cell numbers on each of the SCx were quantified by CCK-8 assay (Fig. 6a), as described above. All experiments were carried out in triplicate. For control, glass substrate coated with PLL, gelatin (PLL-G), Matrigel, and Geltrex were used. To investigate mESC growth in Matrigel or Geltrex, 2D and 3D cell cultivation of mESCs was performed. For 2D Matrigel or Geltrex cultures, 40 μL aliquots of cold Matrigel or Geltrex were diluted tenfold with DPBS and immediately spread into wells of a glass bottom 96-well plate, where the gelation was completed after 2 h at 37 ^o^C. Subsequently, 6000 mESCs were seeded on top of Matrigel- or Geltrex-coated surfaces. For 3D Matrigel or Geltrex culture, 40 μL aliquots of cold undiluted Matrigel or Geltrex were mixed with 6000 mESCs in 5 μL cell culture medium on ice. The resulting cold mixtures were immediately spread into microplate wells where gelation was completed for 30 min at 37 ^o^C.

### Immunofluorescence staining of mESCs on composite materials

To investigate the mESCs pluripotency, 5 × 10^3^ cells were seeded on the fresh SCx materials and allowed to grow for 4 days. For immunofluorescence staining of pluripotency markers, the cells were fixed with 4% PFA in DPBS for 30 min. After permeabilization with a triton X-100 solution (1%, in DPBS) for 1 h, cells were incubated with the primary antibodies (pAb rabbit anti-Nanog, Catalog # ab80892, diluted 1:200, Abcam; mAb IgG mouse anti-Oct4, Catalog # sc5279, diluted 1:200, Santa Cruz Biotechnology; pAb rabbit anti-Sox2, Catalog # ab97959, diluted 1:200, Abcam) for 12 h at 4 °C. Samples were washed with DPBS and subsequently incubated with the secondary antibodies (goat anti-mouse IgG Cy3, Catalog # 115–165–146, diluted 1:200, Dianova; goat anti-rabbit IgG Alexa Fluor^®^488, Catalog # A11070, diluted 1:200, Invitrogen) in 1% BSA in DPBS for 4 h at room temperature. The cell nuclei were counterstained with DAPI. For control, the cells grown on PLL and gelatine-covered PLL were used.

### Immunofluorescence staining of germ layers

After seeding of mESC cells on S100 and cultivation for 4 days in the absence of LIF, the formed spheroids were released via treatment with restriction endonuclease BstEII-HF for 2 h at 37 °C. The released spheroids were allowed to grow in a U shaped plate for another 3 days. The EBs were then cultivated for 2 weeks on FN-coated coverslips, fixed, and permeabilized. The expression of germ layers in the embryoid body (EB) was verified by immunostaining of FoxA2, Brachyury and β-Tubulin III of mESC EB. Immunostaining was achieved with pAb IgG goat anti-FoxA2 (Catalog # AF2400, diluted 1:200)/Brachyury (Catalog # AF2085, diluted 1:200) (R&D Systems) and pAb IgG rabbit anti-β Tubulin III (Catalog # T2200, diluted 1:200, Sigma) using secondary antibodies (Donkey anti-goat IgG Alexa Fluor^®^488 (Catalog # A11055, diluted 1:200)/Alexa Fluor^®^647 (Catalog # A32849, diluted 1:200) and Donkey anti-rabbit IgG Alexa Fluor^®^568 (Catalog # A10042, diluted 1:200), Thermo Fisher Scientific).

### Statistical analysis

All data are represented by mean ± standard deviation (S.D.). Statistical analysis was performed using variance tests (two-way analysis of variance (ANOVA) and one-way ANOVA). Data sets were compared using two tailed, unpaired *t*-test. A value of 0.05 was set as the significance level; the data were marked as (*) *p* < 0.05, (**) *p* < 0.01, and (***) *p* < 0.001. The *p*-value above 0.05 was considered as non-significant (n.s.).

### Reporting summary

Further information on experimental design is available in the Nature Research Reporting Summary linked to this paper.

## Supplementary information

Supplementary Information

Description of Additional Supplementary Files

Supplementary Movie 1

Supplementary Movie 2

Supplementary Movie 3

Reporting summary

## Data Availability

Data supporting the findings of this study are available within the paper and its Supplementary Information files. All other relevant data are available from authors upon reasonable request.
