# Supplementary material for: Carbon-nanotube reinforcement of DNA-silica nanocomposites yields programmable and cell-instructive biocoatings
Source: Nat Commun. 2019 Dec 4;10:5522. doi: 10.1038/s41467-019-13381-1 (PMC6892801; doi:10.1038/s41467-019-13381-1)
Supplement: Supplementary file 1 — Supplementary Information [file 41467_2019_13381_MOESM1_ESM.pdf]

Carbon-Nanotube Reinforcement of DNA-Silica Nanocomposites Yields Programmable and Cell-Instructive Biocoatings

Hu et al.

Supplementary Information

Carbon-Nanotube Reinforcement of DNA-Silica Nanocomposites Yields Programmable and Cell-Instructive Biocoatings

Yong Hu,[a] Carmen M. Domínguez,[a] Jens Bauer,[a] Simone Weigel,[a] Alessa Schipperges,[a] Claude Oelschlaeger,[b] Norbert Willenbacher,[b] Stephan Keppler,[c] Martin Bastmeyer,[c,d] Stefan Heißler,[d] Christof Wöll,[d] Tim Scharnweber,[a] Kersten S. Rabe,[a] and Christof M. Niemeyer[a]*

---

[a] Karlsruhe Institute of Technology (KIT), Institute for Biological Interfaces (IBG 1), Hermann-von-Helmholtz-Platz 1, 76344, Eggenstein-Leopoldshafen, Germany

[b] Karlsruhe Institute of Technology (KIT), Institute for Mechanical Process Engineering and Mechanics, Gotthard-Franz-Straße 3, 76131 Karlsruhe, Germany

[c] Karlsruhe Institute of Technology (KIT), Department of Cell- and Neurobiology, Zoological Institute, Haid-und-Neu-Straße 9, 76131 Karlsruhe, Germany

[d] Karlsruhe Institute of Technology (KIT), Institute of Functional Interfaces (IFG), Hermann-von-Helmholtz-Platz 1, 76344, Eggenstein-Leopoldshafen, Germany

* To whom correspondence should be addressed. E-mail addresses: niemeyer@kit.edu (C. M. Niemeyer)

Contents

This document contains supplementary information and details on the synthesis of multifunctional SiNP, SiNP-P, SiNP-P-T, CNT-P, CNT-P-T and nanocomposite DNA hydrogels, characterization techniques including UV/vis and fluorescence spectroscopy, circular dichroism (CD) spectroscopy, transmission electron microscopy (TEM), scanning transmission electron microscopy (STEM), energy dispersive X-ray spectroscopy (EDS), scanning electron microscopy (SEM), Raman analysis, multiple particle tracking (MPT) microrheology, atomic force microscopy (AFM), dynamic light scattering (DLS) measurements, fluorescence microscopy, fluorescence recovery after photobleaching (FRAP) analysis, static contact angle analysis, melting curve analysis, electrophoresis, and cell experiments including cell culture, cytotoxicity, cell proliferation, cell transmigration, competitive cell adhesion, lateral cell migration, controlled cell release in static and microfluidic conditions as well as stem cell expansion and differentiation

Supplementary Figures

Supplementary Figure 1: Synthesis of oligonucleotide-modified SiNP

Supplementary Figure 2: Supernatant depletion and hybridization assays

Supplementary Figure 3: AFM analysis of CNT-P

Supplementary Figure 4: Raman analysis of CNT-DNA nanocomposite materials

Supplementary Figure 5: Electrophoretic analysis of P1 and T hybridization

Supplementary Figure 6: Electrophoretic analysis of T hybridization with CNT-P

Supplementary Figure 7: Photographs of SiNP-DNA nanocomposite materials

Supplementary Figure 8: Fluorescence imaging and optical absorbance analysis of the polymerisation process of SiNP-DNA nanocomposite materials.

Supplementary Figure 9: MPT analysis of SiNP-DNA nanocomposite materials

Supplementary Figure 10: FRAP analysis of the diffusion coefficients of FITC-dUTP and FITC-dextran

Supplementary Figure 11: TEM analysis of SiNP-DNA nanocomposite materials

Supplementary Figure 12: SEM analysis of SiNP-DNA nanocomposite materials prepared by RCA polymerisation of SiNP-P for variable times

Supplementary Figure 13: SEM analysis of CNT-DNA nanocomposite materials prepared by RCA polymerisation of variable concentrations of CNT-P

Supplementary Figure 14: SEM analysis of SiNP/CNT-DNA nanocomposite materials prepared by RCA polymerisation of variable concentrations and amounts of SiNP and CNT

Supplementary Figure 15: High resolution SEM analysis of dried nanocomposite materials.

Supplementary Figure 16: AFM analysis of dried SiNP/CNT-DNA nanocomposite materials.

Supplementary Figure 17: Elemental analysis of SiNP-DNA nanocomposite materials

Supplementary Figure 18: Incorporation of fluorescence into SiNP-DNA nanocomposite materials

Supplementary Figure 19: SEM analysis of the ultrastructure of fluorescent SiNP-DNA nanocomposite materials

Supplementary Figure 20: Electrophoretic analysis of endonuclease digestion of SiNP-DNA nanocomposite

Supplementary Figure 21: Melting curve of SiNP-DNA nanocomposite materials

Supplementary Figure 22: The engineered DNA hydrogel backbone shows the typical properties of double-stranded DNA.

Supplementary Figure 23: Specific endonuclease degradation of S100 bearing a DNA backbone with enzymatic restriction sites

Supplementary Figure 24: Stability of SiNP-DNA nanocomposite material S100 against non-specific digestion by DNase I

Supplementary Figure 25: Long-term stability of nanocomposite material S100 in media

Supplementary Figure 26: Cytocompatibility of SiNP/CNT-DNA nanocomposite materials

Supplementary Figure 27: Live cell staining of MCF7 cells grown on fresh binary and ternary SiNP/CNT-DNA nanocomposite materials

Supplementary Figure 28: Live cell staining of MCF7 cells grown on dried binary and ternary SiNP/CNT-DNA nanocomposite materials

Supplementary Figure 29: Production of libraries of SiNP/CNT-DNA nanocomposite materials. Fluorescence image of an array of Cy5-labeled

binary and ternary SiNP/CNT-DNA nanocomposite materials

Supplementary Figure 30: Static water contact angle (WCA) analysis of dried SiNP/CNT-DNA nanocomposite surfaces

Supplementary Figure 31: Bright field imaging analysis of MCF7 cells adhered on dried SiNP/CNT-DNA nanocomposite materials

Supplementary Figure 32: Fluorescence microscopy analysis of $MCF7_{eGFP}$ cells adhered on SC50

Supplementary Figure 33: Phalloidin staining of actin cytoskeleton and immunostaining of Paxillin and pPAX of MCF7 cells grown on dried SC50

Supplementary Figure 34: Bright field imaging analysis of REF52 cells adhered on dried binary and ternary SiNP/CNT-DNA nanocomposite materials

Supplementary Figure 35: Live cell staining of REF52 cells grown on dried SiNP/CNT-DNA nanocomposite materials

Supplementary Figure 36: Proliferation rate of REF52 cells grown on dried binary and ternary SiNP/CNT-DNA nanocomposite materials

Supplementary Figure 37: Fluorescence microscopy analysis of $MCF7_{eGFP}$ cells cultivated on dried SiNP-P

Supplementary Figure 38: Competitive adhesion of $MCF7_{eGFP}$ cells to either PLL or dried SC50

Supplementary Figure 39: Directional lateral migration of $MCF7_{eGFP}$ cells

Supplementary Figure 40: Relationship between CNT fraction, water contact angle, surface roughness and cell adhesion.

Supplementary Figure 41: Vertical transmigration of $MCF7_{eGFP}$ into fresh SC50

Supplementary Figure 42: Distribution of $MCF7_{eGFP}$ cells transmigrated into fresh SC50

Supplementary Figure 43: Trapping and release of $MCF7_{eGFP}$ cells using fresh SC25

Supplementary Figure 44: Quantification of $MCF7_{eGFP}$ cells located on the glass bottom underneath the SC25 layer, determined before and after endonuclease digestion

Supplementary Figure 45: REF52 cells transmigrate into fresh SC50 but cannot migrate through SC25

Supplementary Figure 46: Microfluidic setup for cell experiments

Supplementary Figure 47: Cultivation of $MCF7_{eGFP}$ cells released from SC25-coated microfluidic channel

Supplementary Figure 48: Live cell staining of MCF7 cells released from an SC25-coated microfluidic channel

Supplementary Figure 49: Quantification of time-dependent mESC growth on various substrata

Supplementary Figure 50: Size distribution of mESCs on the various composite and control materials

Supplementary Figure 51: Immunostaining of Nanog, Sox2 and Oct4 of mESCs grown on Matrigel and Geltrex

Supplementary Figure 52: Immunostaining of Nanog and Oct4 of mESCs grown on SiNP/CNT-DNA nanocomposite materials

Supplementary Figure 53: Immunostaining of Sox2 and Oct4 of mESCs grown on SiNP/CNT-DNA nanocomposite materials

Supplementary Figure 54: Bright field imaging analysis of mESCs grown on PLL, PLL+G, Matrigel, Geltrex and S100

Supplementary Figure 55: Bright field imaging analysis of mESC colonies before and after release from culture materials

Supplementary Figure 56: Release of mESCs from pNIPAM surfaces

Supplementary Figure 57: Bright field images of mESC spheroid (embryoid body) extracted from S100

Supplementary Tables

Supplementary Table 1: MPT analysis of binary and ternary SiNP/CNT-DNA nanocomposite materials

Supplementary Table 2: MPT analysis of SiNP-DNA nanocomposite materials using tracer particles of different size

Supplementary Table 3: MPT analysis of SiNP-DNA nanocomposite materials prepared by RCA polymerisation of SiNP for variable times

Supplementary Table 4: MPT analysis of CNT-DNA nanocomposite materials using tracer particles of different size

Supplementary Table 5: MPT analysis of CNT-DNA nanocomposite materials prepared by RCA polymerisation of CNT at different concentrations

Supplementary Table 6: List of DNA sequences

References

Supplementary Figures

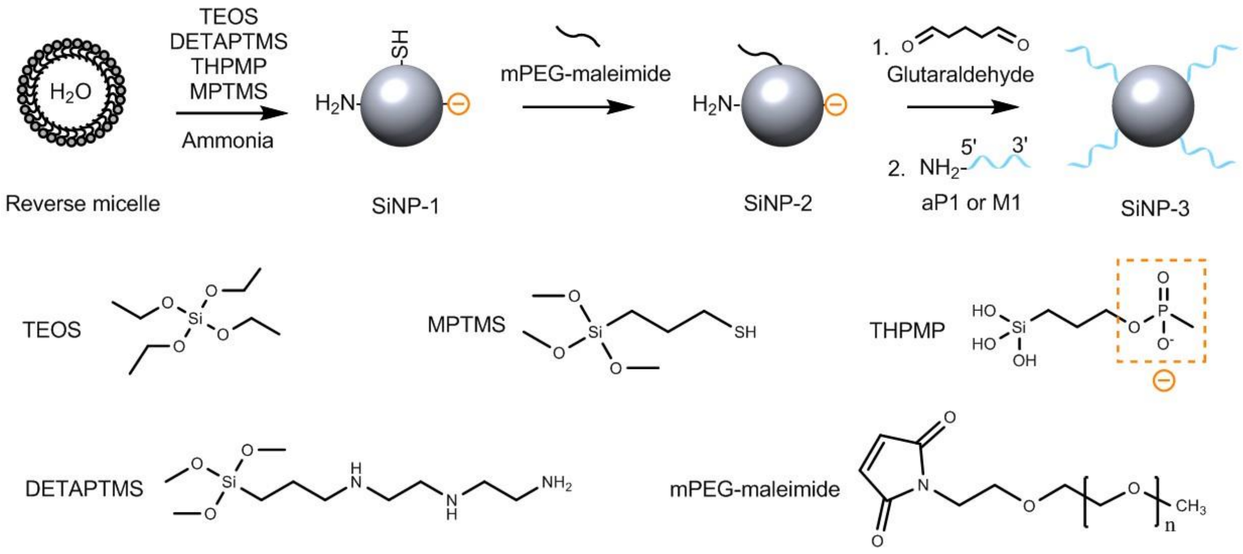

Supplementary Figure 1. Schematic illustration of the synthesis of oligonucleotide-modified SiNP. Synthesis of multifunctional oligonucleotide-modified SiNP containing primer strand P1 for RCA (SiNP-P) or M1 strand as a control for hybridization assays shown in Supplementary Figure 2 (SiNP-M1).

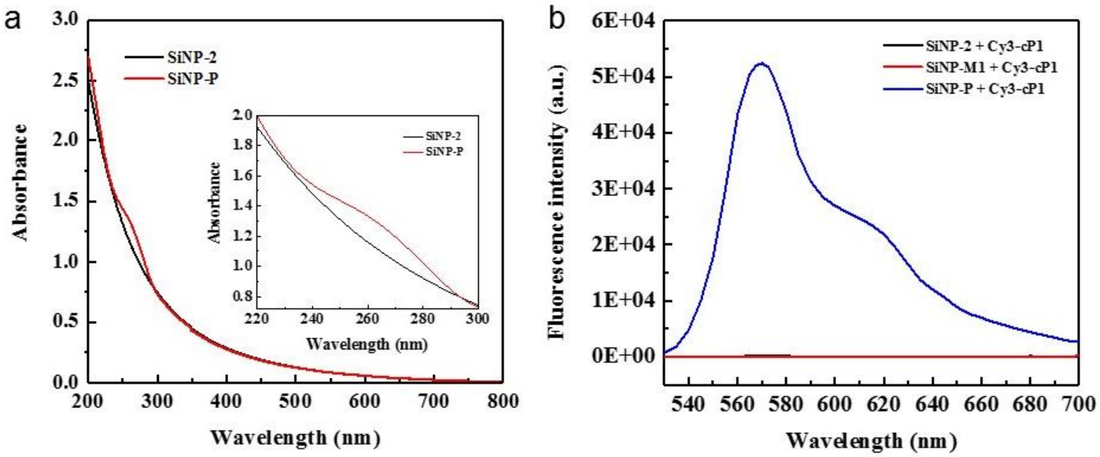

Supplementary Figure 2. Supernatant depletion and hybridization assays. **a**, UV-vis spectra of SiNP-2 before and after (SiNP-P) chemical modification of amine-P1 (aP1). The inset shows the enlarged spectra from 220-300 nm. Note the prominent absorption peak at 260 nm, indicating the presence of P1 strands on SiNP-P. The amount of P1 strands was quantified with supernatant depletion assays to be about 120 DNA strands per particle, which is similar to results from earlier studies[1]. **b**, Fluorescence spectra of SiNP-2 (black), SiNP-M1 (red), and SiNP-P (blue) obtained after hybridization with Cy3-labeled probe strand Cy3-cP1, complementary to RCA primer P1. As expected, only SiNP-P can bind the Cy3-labeled cP1. Quantitative analysis using calibration curves indicated that about 100 cP1 molecules were hybridized per particle.

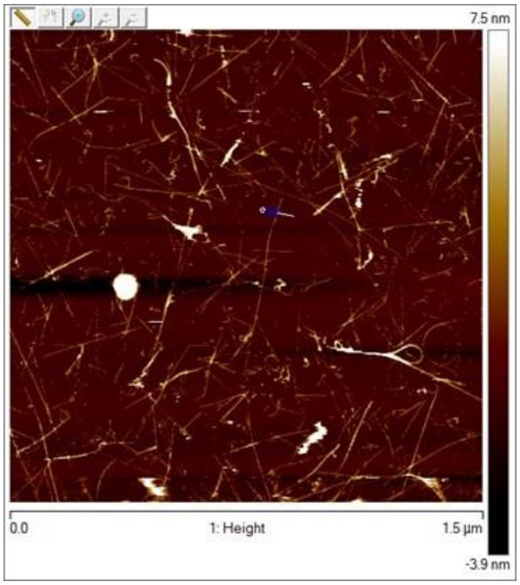

Supplementary Figure 3. AFM analysis of CNT-P. AFM image of P2 modified CNT. Note that the CNT are well dispersed and only few CNT bundles are present in the sample.

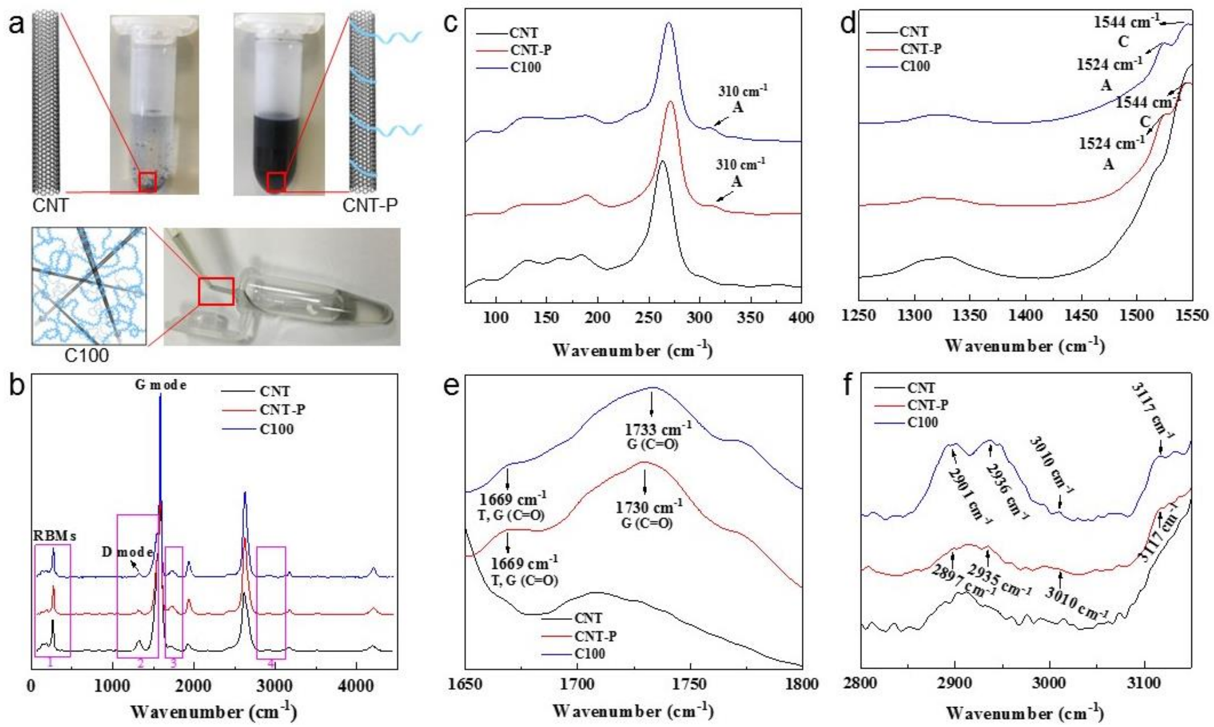

Supplementary Figure 4. Raman analysis of CNT-DNA nanocomposite materials. Photographs (**a**) and Raman spectra (**b**) of CNT, CNT-P and C100. All spectra are normalized to their G-band intensities. (**c**), (**d**), (**e**), and (**f**) are extrapolated from ranges 1, 2, 3, and 4, respectively. The bands in (**f**) are all contributed by C-H bonds of DNA nucleobases, A, T, C, and G (adenine, thymine, cytosine, and guanine, respectively). Note that the characteristic modes (namely radial breathing mode (RBM) around 270 cm^{-1}, D mode around 1320 cm^{-1} and 2600 cm^{-1}, and G mode around 1590 cm^{-1}) of single-walled CNT were detected[2]. The RBM corresponds to radial expansion and contraction of the nanotube where all the carbon atoms are moving in phase (acoustic phonons) in the radial direction[3] and its wavenumber (cm^{-1}) depends on the diameter of the nanotube given by the equation[4, 5].

$$\nu_{RBM} = [223/d] + 10 \qquad (1)$$

where ν_{RBM} is the RBM frequency and d is the corresponding diameter (in nanometers).

By using Supplementary Equation (1), the "theoretical diameters" of CNT present in CNT-P (RBM frequency at 271.0 cm^{-1}) and C100 (RBM frequency at 270.0 cm^{-1}) were calculated to be 0.854 and 0.858 nm, respectively (**b**). These diameters are smaller than the diameter of pure CNT (0.881 nm calculated at 263 cm^{-1}). The relative RBM band intensity of CNT (I_{RBM}/I_G) decreased from 0.172 (CNT) to 0.157 (CNT-P) and 0.165 (C100), respectively. These changes are due to the restriction of carbon atoms in the radial direction by DNA wrapping. The existence of DNA on CNT surface is also indicated in the enlarged spectra in **c-f**, where the characteristic bands were assigned to the corresponding bonds in DNA[6].

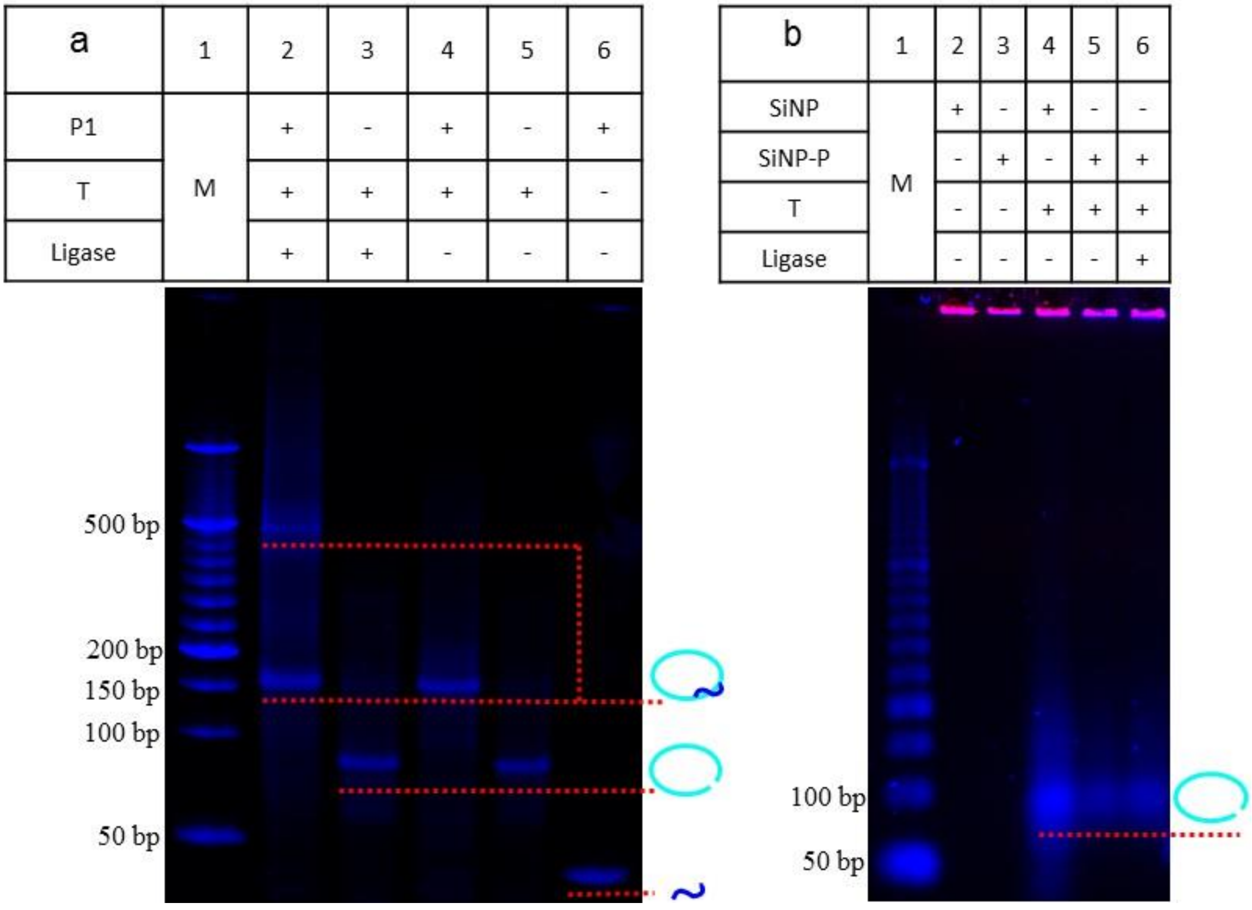

Supplementary Figure 5. Electrophoretic analysis of P1 and T hybridization. **a**, Native polyacrylamide gel (6%) electrophoresis (PAGE) analyzing the hybridization of P1 (blue) and T (cyan). After incubation of a mixture of T and P1 for 3 h at 25°C (lane 4), the appearance of a band with reduced electrophoretic mobility in comparison with T (lane 5) and P1 (lane 6), confirms the formation of the primer-template (P-T) complex with high efficiency. Addition of T4 DNA ligase and incubation for 3 h at 25 °C (lane 2) results in a band with even lower mobility, suggesting the formation of P-T oligomers which can occur in solution as a competing reaction of the cyclization. **b**, Agarose (2.5%) electrophoresis analyzing the hybridization of Cy5@SiNP-P (magenta bands) and T (blue). After incubation of a mixture of Cy5@SiNP-P and T, the amount of free T (Lane 4) is significantly decreased (lanes 5, 6) due to immobilization on SiNP by hybridization of T and P1. All electrophoreses were carried out in 1× TAE-Mg^{2+} at 120 V for 45 min. The gels were stained with SYBR Gold.

	1	2	3	4	5
CNT-P	+	-	-	+	+
CNT-M2	-	-	+	-	-
T	-	+	+	+	+
Ligase	-	-	-	-	+

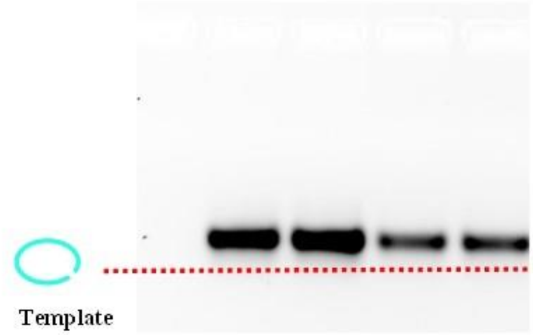

Supplementary Figure 6. Electrophoretic analysis of T hybridization with CNT-P. The intensity of the band representing ssDNA template T (lane 2) decreases due to hybridization with CNT-bound primer P (lanes 4, 5), whereas CNT containing non-complementary oligonucleotide M2 do not lead to affect the band intensity of T (lane 3). Note that DNA-modified CNT-P and CNT-M2 are not visible because CNT were retained in the gel pockets and the fluorescence of EtBr was quenched by CNT, as observed earlier[7]. This is an agarose (2.5%) electrophoresis in 1× TAE-Mg^{2+} at 120 V for 45 min. The gel was stained with EtBr.

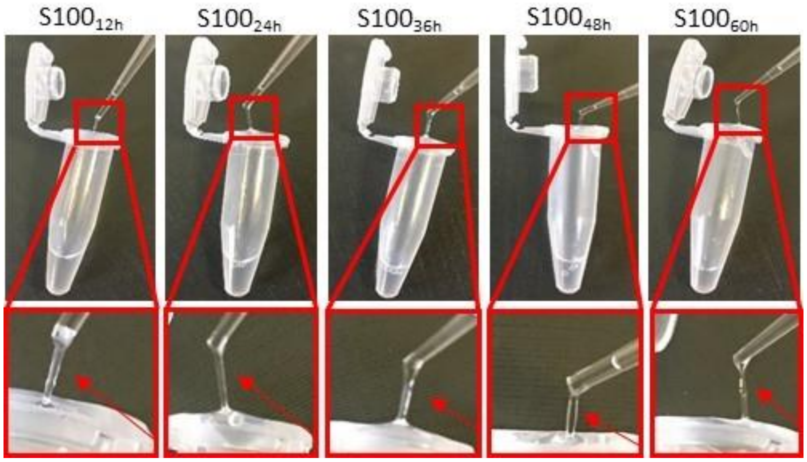

Supplementary Figure 7. Photographs of SiNP-DNA nanocomposite materials. Photographs of S100 obtained after variable times of RCA reaction at 30 ºC. The red arrows point at elastic filaments of the hydrogels that can be stretched like a rubber band.

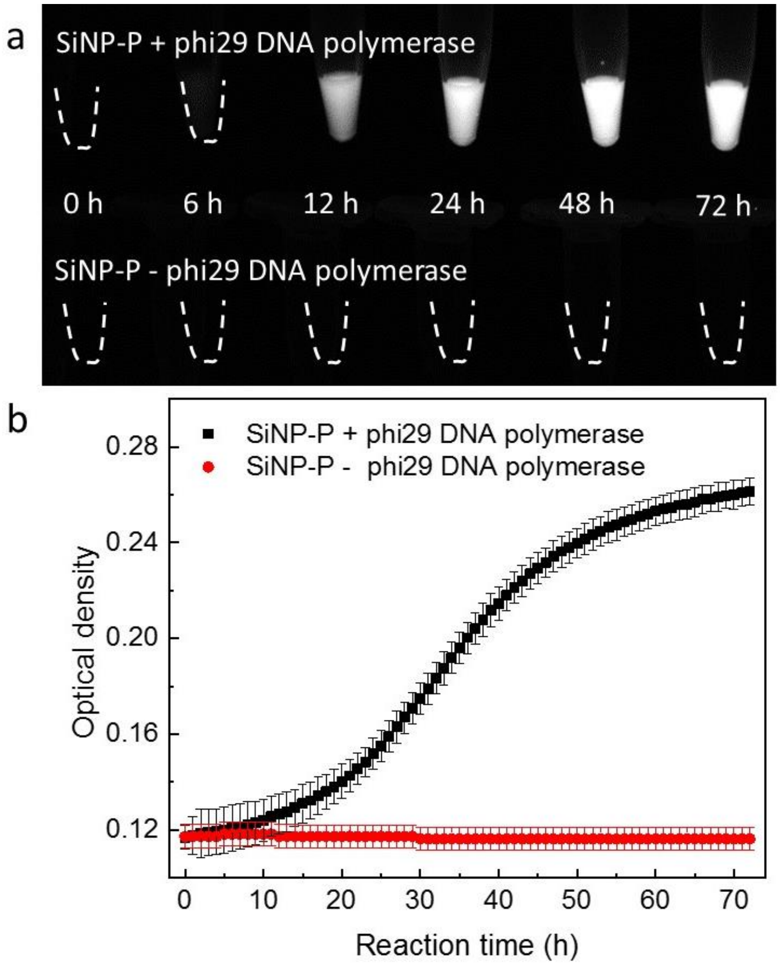

Supplementary Figure 8. Fluorescence imaging and optical absorbance analysis of the polymerisation process of SiNP-DNA nanocomposite materials. **a**, Fluorescence images of polymerised reaction mixtures, stained at the given times with SYBR Green I (1× TAE-Mg^{2+}). (**b**) Time course of optical density determined at 600 nm of the RCA mixture in the presence or absence of phi29 DNA polymerase. All curves are plotted by mean ± S.D. of triplicate samples. Note that turbidity of the polymerisation mixtures increases with increasing reaction times.

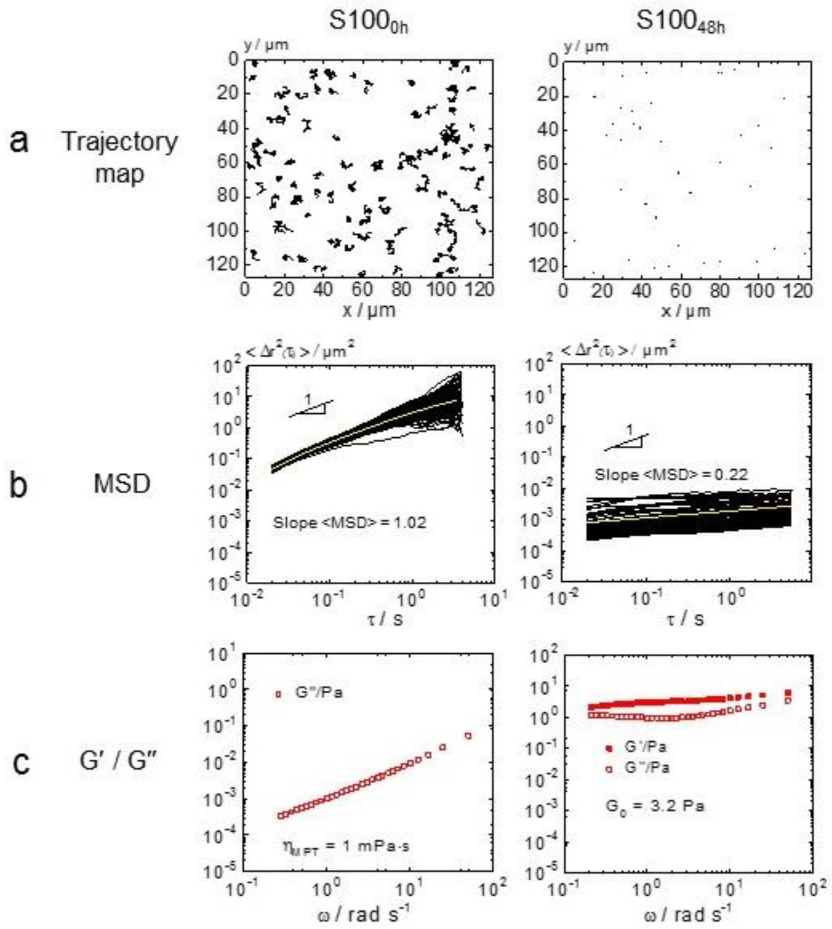

Supplementary Figure 9. MPT analysis of SiNP-DNA nanocomposite materials. Trajectory maps (**a**) and MSD values (**b**) of tracer particles with a diameter of 0.5 µm incorporated in the polymerisation mixtures of SiNP-P that were subjected to RCA for 0 h (left column) and 48 h (right column). The corresponding plots of shear-moduli G' (closed symbols) and G'' (open symbols) as a function of frequency are shown in (**c**). The yellow curve in (**b**) represents the average MSD. Note that the slope <MSD> of tracer particles in S100$_{0h}$ was calculated to be 1.02, indicating that particles' motion is purely diffusive and that the microenvironment surrounding the particles responds like a viscous liquid. The viscosity η_{MPT} of the purely viscous fluid has been determined from MSD tracer particles varying linearly with time using the equation:

$$\Delta r^2 = 4D\tau \qquad (2)$$

in combination with the Stokes-Einstein equation:

$$D = \frac{k_B T}{6\pi a \eta_{MPT}} \qquad (3)$$

where D is the diffusion coefficient, a is the tracer particle radius, k_B is the Boltzmann constant and T the absolute temperature.

The plateau modulus G_0 of viscoelastic materials has been determined using the equation:

$$G_0 = \frac{2k_B T}{3\pi a \Delta r^2} \qquad (4)$$

where Δr^2 is the average mean square displacement value in the plateau region where Δr^2 is independent of time.

Accordingly, the η_{MPT} of $S100_{0h}$ was calculated to be 1 mPa·s, similar to that of water, further demonstrating the viscous property of $S100_{0h}$. The elasticity of $S100_{48h}$ is indicated by both the higher slope <MSD> = 0.22 of tracer particles and the corresponding G' values that are constantly higher than G'' in the frequency range of 0.2 rad s^{-1} - 50 rad s^{-1}.

Supplementary Table 1. MPT analysis of binary and ternary SiNP/CNT-DNA nanocomposite materials. Synthesis of ternary SCx nanocomposites *via* RCA using SiNP and CNT at different mass ratios in the presence of tracer particles with the diameter of 0.5 µm for S100 or 1.0 µm for the rest materials. The given slope data obtained from MPT analyses indicate that the mechanical stiffness of SCx materials increases with increasing amounts of CNT at the same amount of SiNP/with the increasing of SiNP at the same amount of CNT. Note that both 0.2 µm and 0.5 µm tracer particles freely diffuse inside the materials, whereas 1.0 µm tracer are restrained by SCx materials, indicating the mesh size of SCx is in the range of 0.5 - 1.0 µm.

Entry	Name[a]	[SiNP-P][b]	[CNT-P][b]	Slope <MSD>[c]
1	S100	4000	-	0.22 ± 0.02
2	SC50	4000	80	0.20 ± 0.02
3	SC25	4000	160	0.15 ± 0.02
4	SC12.5	4000	320	0.11 ± 0.01
5	SC6.25	2000	320	0.14 ± 0.01
6	SC2.5	800	320	0.24 ± 0.02
7	C100	-	320	0.24 ± 0.02

[a)] Acronyms represent the mass ratio of SiNP-P:CNT-P

[b)] Given in µg mL^{-1}

[c)] Determined after 48 h RCA reaction time.

Supplementary Table 2. MPT analysis of SiNP-DNA nanocomposite materials using tracer particles of different size. Synthesis of S100$_{48h}$ *via* RCA reaction in the presence of tracer particles with different diameters. Note that the slope <MSD> of tracer particles with a diameter of 0.2 µm in S100$_{48h}$ is close to 1, indicating that 0.2 µm tracer particles freely diffuse inside the S100$_{48h}$. In contrast, 0.5 µm tracer particles are restrained inside the S100$_{48h}$ gel. Therefore, the mesh size of S100$_{48h}$ was estimated to be in the range of 0.2 - 0.5 µm. Also note that 1.0 µm tracer particles were not suitable to the S100$_{48h}$ gel because these particles aggregated in the reaction mixture. Therefore, 0.5 µm tracer particles were used for time course studies of the viscoelasticity of S100 (Supplementary Table 3).

Entry	Diameter of tracer particle	Slope <MSD>	η_{MPT} (mPa·s)	G_0 (Pa)
1	0.2 µm	0.96 ± 0.10	1.5 ± 0.2	N.A.[a]
2	0.5 µm	0.22 ± 0.02	N.A.[a]	3.2 ± 0.4
3	1.0 µm	N.A.[a]	N.A.[a]	N.A.[a]

[a)] N.A.: not applicable.

Supplementary Table 3. MPT analysis of SiNP-DNA nanocomposite materials prepared by RCA polymerisation of SiNP for variable times. Note that the RCA products obtained after 0 h and 6 h do not show viscoelastic properties of typical hydrogels, because the slope <MSD> ≈ 1 indicates a purely viscous medium.

Entry	S100$_{xh}$ [a]	Reaction time (h)	Slope <MSD>	η_{MPT} (mPa·s)	G_0 (Pa)
1	S100$_{0h}$	0	1.02 ± 0.01	1.0 ± 0.1	N.A.[b]
2	S100$_{6h}$	6	0.99 ± 0.10	1.2 ± 0.1	N.A.
3	S100$_{12h}$	12	0.40 ± 0.04	N.A.	0.9 ± 0.1
4	S100$_{24h}$	24	0.30 ± 0.03	N.A.	1.7 ± 0.2
5	S100$_{36h}$	36	0.25 ± 0.03	N.A.	3.0 ± 0.3
6	S100$_{48h}$	48	0.22 ± 0.02	N.A.	3.2 ± 0.4
7	S100$_{60h}$	60	0.15 ± 0.02	N.A.	3.4 ± 0.3

[a)] S100$_{xh}$: S100 gels obtained after x h of RCA reaction time.

Supplementary Table 4. MPT analysis of CNT-DNA nanocomposite materials using tracer particles of different size. Preparation of C100 materials by RCA for 48 h in the presence of tracer particles with different diameters. Note that 0.2 µm and 0.5 µm tracer particles freely diffuse inside C100, whereas 1.0 µm tracer particles were restrained. Therefore, the mesh size of C100 was estimated to be in the range of 0.5 µm to 1.0 µm, thus being larger than that of S100. Presumably this change is a consequence of the geometric difference between column-like CNT (diameter = 0.83 nm, length = 1.0 µm) and spherical SiNP (diameter = 80 nm). 1.0 µm tracer particles were chosen for the analysis of the viscoelasticity of C100 materials containing variable CNT concentrations (Supplementary Table 5).

Entry	Diameter of tracer particle	Slope <MSD>	η_{MPT} (mPa·s)	G_0 (Pa)
1	0.2 µm	1.01 ± 0.10	1.0 ± 0.0	N.A.
2	0.5 µm	0.97 ± 0.1	11.35 ± 0.1	N.A.
3	1.0 µm	0.24 ± 0.02	N.A.	2.8 ± 0.2

Supplementary Table 5. MPT analysis of CNT-DNA nanocomposite materials prepared by RCA polymerisation of CNT at different concentrations. Characterization of C100 materials obtained by RCA for 48 h at different concentrations of CNT. 1.0 µm diameter tracer particles were used. Note that the slopes <MSD> decrease and the G_0 values increase with increasing CNT concentrations, indicating that the elastic properties of the materials are reinforced with increasing amounts of CNT. However, the hydrogel was very soft when the concentration of CNT was too low (*e.g.*, 0-160 µg mL^{-1}). On the other hand, a higher amount of CNT in the reaction mixture led to agglomeration and the formation of brittle materials with no apparent elasticity when the CNT concentration was too high (*e.g.*, 640 µg mL^{-1}). In the latter case, the calculation of slope <MSD> and G_0 was not possible.

Entry	Cx[a]	[CNT-P][c]	Slope <MSD>	G_0 (Pa)
1	C0[b]	0	0.62 ± 0.06	0.1 ± 0.0
2	C25	80	0.54 ± 0.05	0.2 ± 0.0
3	C50	160	0.36 ± 0.04	0.7 ± 0.1
4	C100	320	0.24 ± 0.02	2.8 ± 0.2
5	C200	640	N.A.	N.A.

a) The value x denotes the mass fraction of CNT in the RCA mixture with respect to optimal SiNP concentration, similar as in Table 1, main text

b) The sample C0 is a pure DNA hydrogel generated by RCA using an equal amount of primer as present in the C100 mixture

c) Given in µg mL^{-1}.

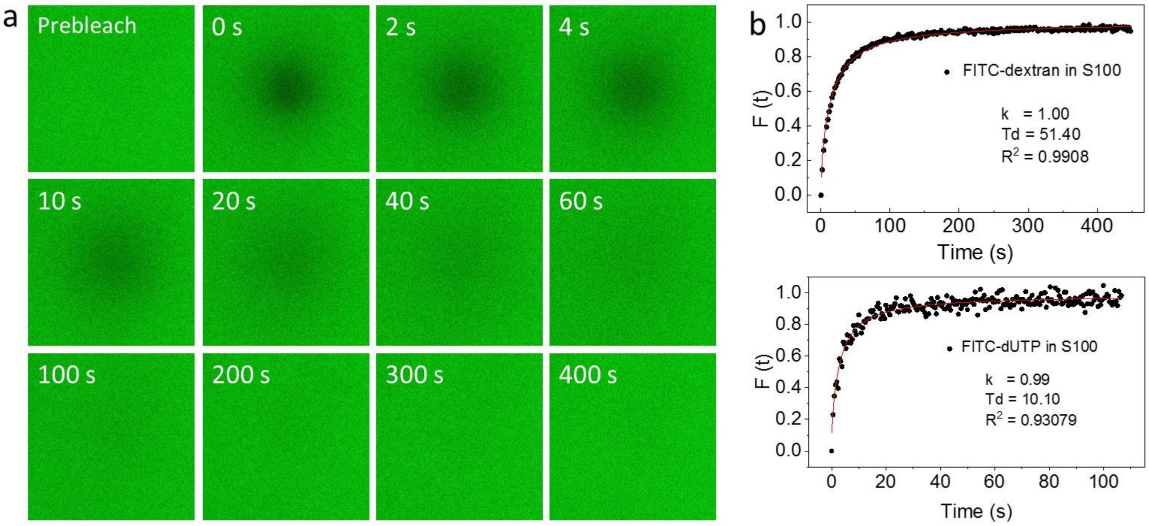

c

Materials	PBS	RCA mixture	S100	SC50	SC25	SC12.5	SC6.25	SC2.5	C100	Matrigel	Geltrex
D^a ($\mu m^2\ s^{-1}$)	54.4 ± 2.3	51.9 ± 2.3	12.0 ± 0.2	12.4 ± 1.1	12.9 ± 0.2	13.2 ± 1.0	18.9 ± 2.3	24.5 ± 3.9	32.3 ± 4.1	27.7 ± 8.1	21.1 ± 0.2
D^b ($\mu m^2\ s^{-1}$)	146.0 ± 8.7	135.2 ± 9.4	62.0 ± 2.5	61.6 ± 2.8	62.9 ± 2.4	73.8 ± 3.9	95.7 ± 5.1	103.6 ± 7.1	127.8 ± 7.7	110.2 ± 6.8	112.6 ± 5.6

Supplementary Figure 10. FRAP analysis of the diffusion coefficients of FITC-dUTP and FITC-dextran. **a**, Representative images obtained from FRAP experiment of S100 stained with FITC-dextran (70 kDa). **b**, Representative fluorescence recovery curves of FITC-dextran (top) and FITC-dUTP (bottom) extracted from the microscopy image stacks. The experimental data (•) and fitted curve (red solid line) were obtained from least-square fits using Supplementary Equation (7) to yield the mobile fraction *k* and the diffusion coefficient *D* (**c**). D^a and D^b represent the diffusion coefficients of FITC-dextran and FITC-dUTP, respectively.

Discussion

The diffusion coefficients of large (D^a, represented by FITC-dextran) and small molecules (D^b, represented by FITC-dUTP) in the SCx are about 2-fold lower (S100-SC12.5) or approximately equal (SC6.25-C100) than those observed in conventional gels (i.e. Geltrex and Matrigel). It is well accepted that a sufficient nutrient supply of the cells in artificially produced tissues is of crucial importance for an optimal development and survival of these tissues[8]. Given the improved adhesion and growth behaviour observed in this study, the possibly slightly reduced mass transport in the SCx materials does not seem to have a negative effect on the nutrient supply of the cells. It could therefore be that under limited diffusion conditions the paracrine substances (growth factors) released by the cells enclosed in the SCx are more highly concentrated, thus creating a more attractive extracellular environment, as has been discussed with other hydrogels[9].

Methodology

To extract the experimental recovery curve from the image stack, the mean fluorescence intensities inside the bleached region, $I_{frap}(t)$, and inside a reference region, $I_{ref}(t)$, were calculated for each time point t using the Zeiss software Zen (black edition). In the next step, $I_{frap}(t)$ was

normalized to the prebleach intensity, $I_{\text{frap}}(\text{pre})$, and corrected for any bleaching effects that might have occurred during image acquisition:

$$f(t) = \frac{I_{\text{ref}}(\text{pre})}{I_{\text{ref}}(t)} \cdot \frac{I_{\text{frap}}(t)}{I_{\text{frap}}(pre)} \qquad (5)$$

Here, $f(t)$ is the normalized fluorescence intensity inside the bleached region, and Iref (pre) is the fluorescence intensity inside the reference region before bleaching. In the following step, $f(t)$ was further normalized to the full scale using:

$$F(t) = \frac{f(t) - f(0)}{f(\text{pre}) - f(0)} \qquad (6)$$

where $f(0)$ is the normalized fluorescence intensity immediately after bleaching, and f(pre) the normalized fluorescence intensity before bleaching. Finally, the characteristic diffusion time T_D and the mobile fraction k were determined by a least-square fit of the following expression to the experimental recovery curve:

$$F(t) = k \cdot e^{-\frac{T_D}{2t}} \left[I_0\left(\frac{T_D}{2t}\right) + I_1\left(\frac{T_D}{2t}\right) \right] \qquad (7)$$

where I_0 and I_1 are the modified Bessel functions of the first kind of zero and first order. This FRAP model has been previously described by Soumpasis[10] and Brandl[11]. The diffusion coefficient D was then calculated by:

$$D = \frac{w^2}{T_D} \qquad (8)$$

where w is the radius of the bleached spot (25 μm in all experiments).

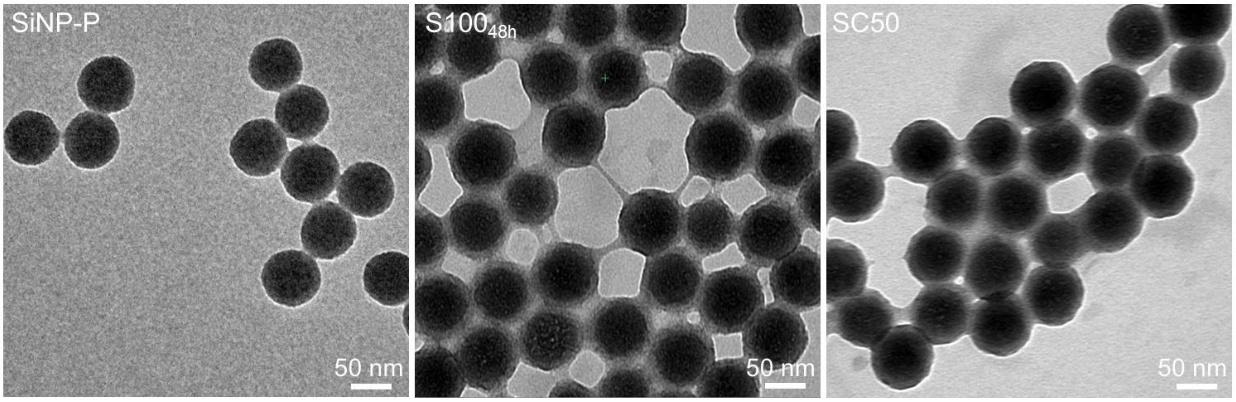

Supplementary Figure 11. TEM analysis of SiNP/CNT-DNA nanocomposite materials. Representative TEM images of SiNP-P, $S100_{48h}$, and SC50. Note that, the DNA linkers between SiNP are clearly visible after 48 h of RCA polymerization, whereas CNT in the SC50 cannot be visualized due to their small diameter. Better visualization of CNT in the nanocomposite materials could be achieved by SEM analysis, as shown in Figure 2c, d and Supplementary Figure 15.

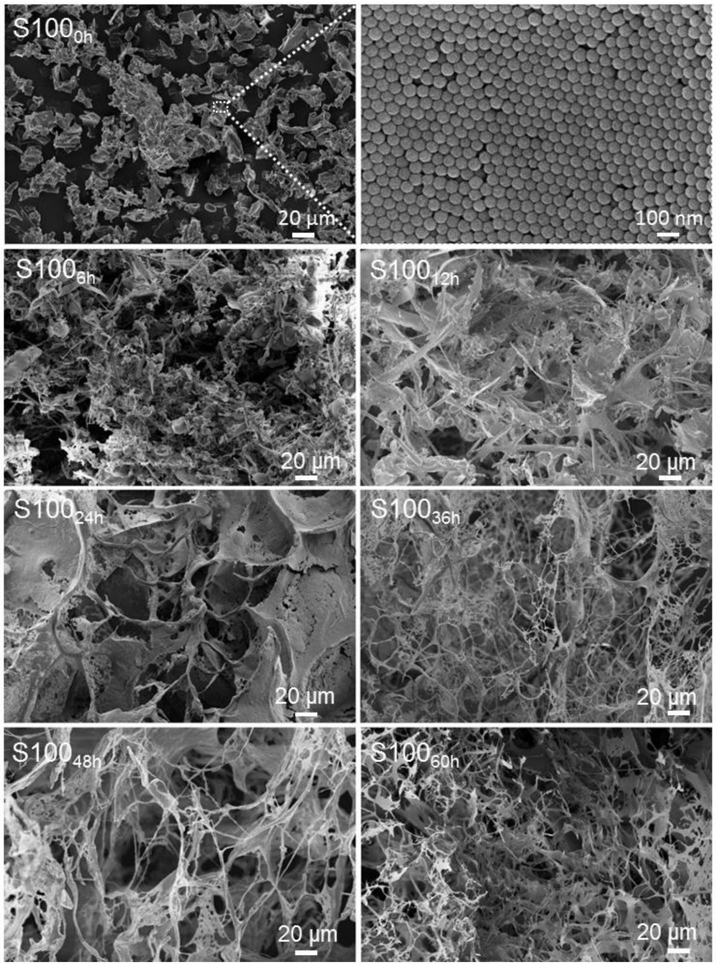

Supplementary Figure 12. SEM analysis of the hierarchical ultrastructure of SiNP-DNA nanocomposite materials prepared by RCA polymerisation of SiNP-P for variable times. Representative SEM images of S100 samples drawn at variable time points during RCA polymerisation. Note that the sample drawn at t = 0 (S100$_{0h}$) only shows SiNP particles that aggregate into superlattices in the course of dehydration during SEM specimen preparation.

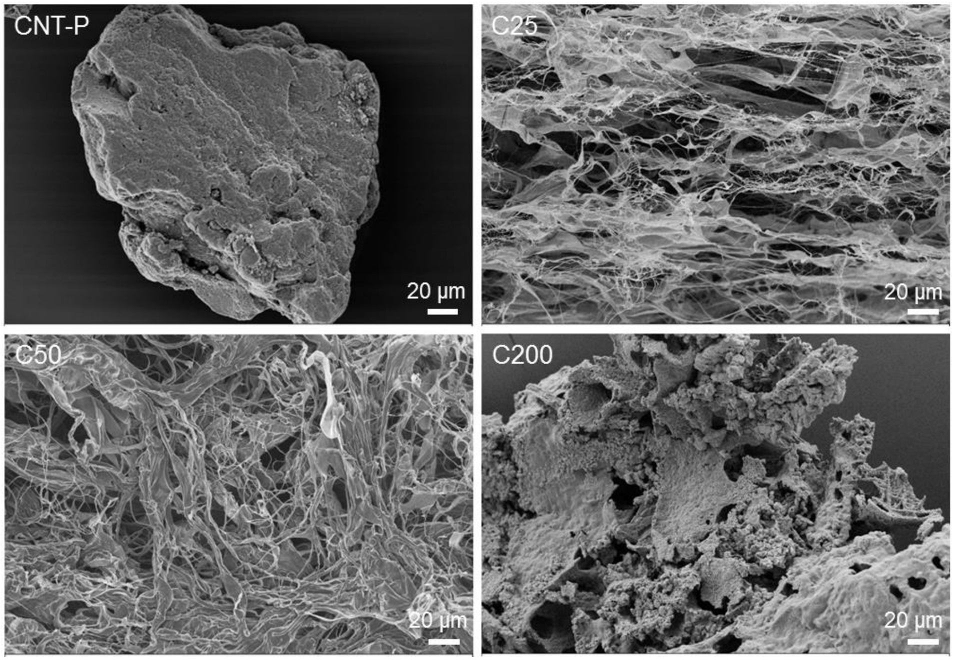

Supplementary Figure 13. SEM analysis of CNT-DNA nanocomposite materials prepared by RCA polymerisation of variable concentrations of CNT-P. Representative SEM images of CNT-P and CNT-DNA composites with variable amounts of CNT (C25, C50, and C200, as indicated in Supplementary Table 5).

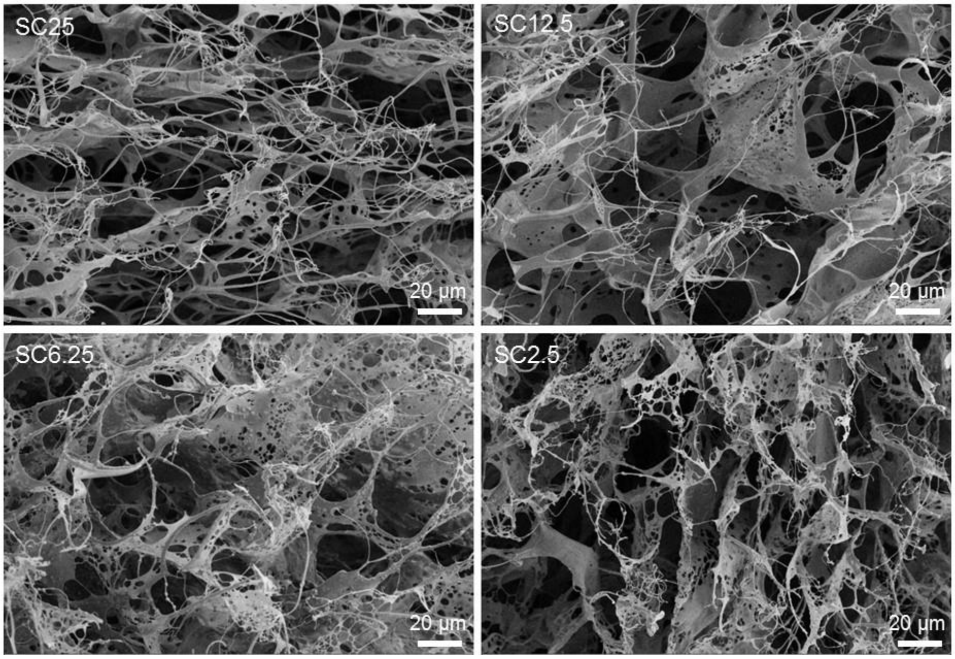

Supplementary Figure 14. SEM analysis of SiNP/CNT-DNA nanocomposite materials prepared by RCA polymerisation of variable concentrations and amounts of SiNP and CNT. Representative SEM images of SC25, SC12.5, SC6.25, and SC2.5.

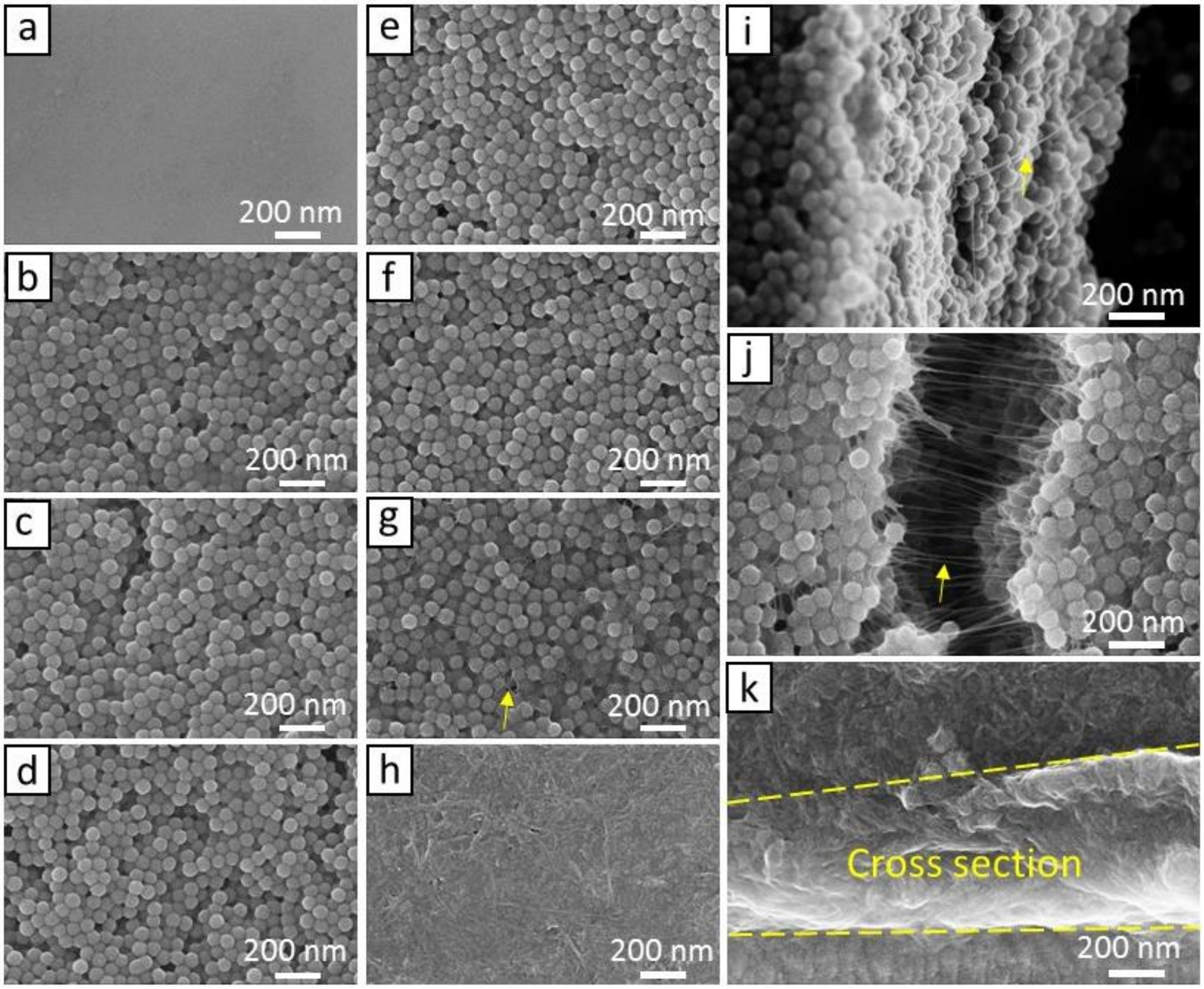

Supplementary Figure 15. High resolution SEM analysis of dried nanocomposite materials. Representative SEM images of (**a**) PLL surface, (**b**) S100, (**c**, **i**) SC50, (**d**) SC25, (**e**) SC12.5, (**f**) SC6.25, (**g**, **j**) SC2.5, and (**h**, **k**) C100, respectively. The arrows point at CNT. Note that the SiNP/CNT-DNA nanocomposite materials show the nanoroughness expected from the incorporated SiNP whereas the PLL surface has no apparent nanoscale features. Also note that the incorporated CNT are visible in magnified images of cracked regions of C100 (**k**), SC2.5 (**j**), and even SC50 (**i**) containing lowest amounts of CNT.

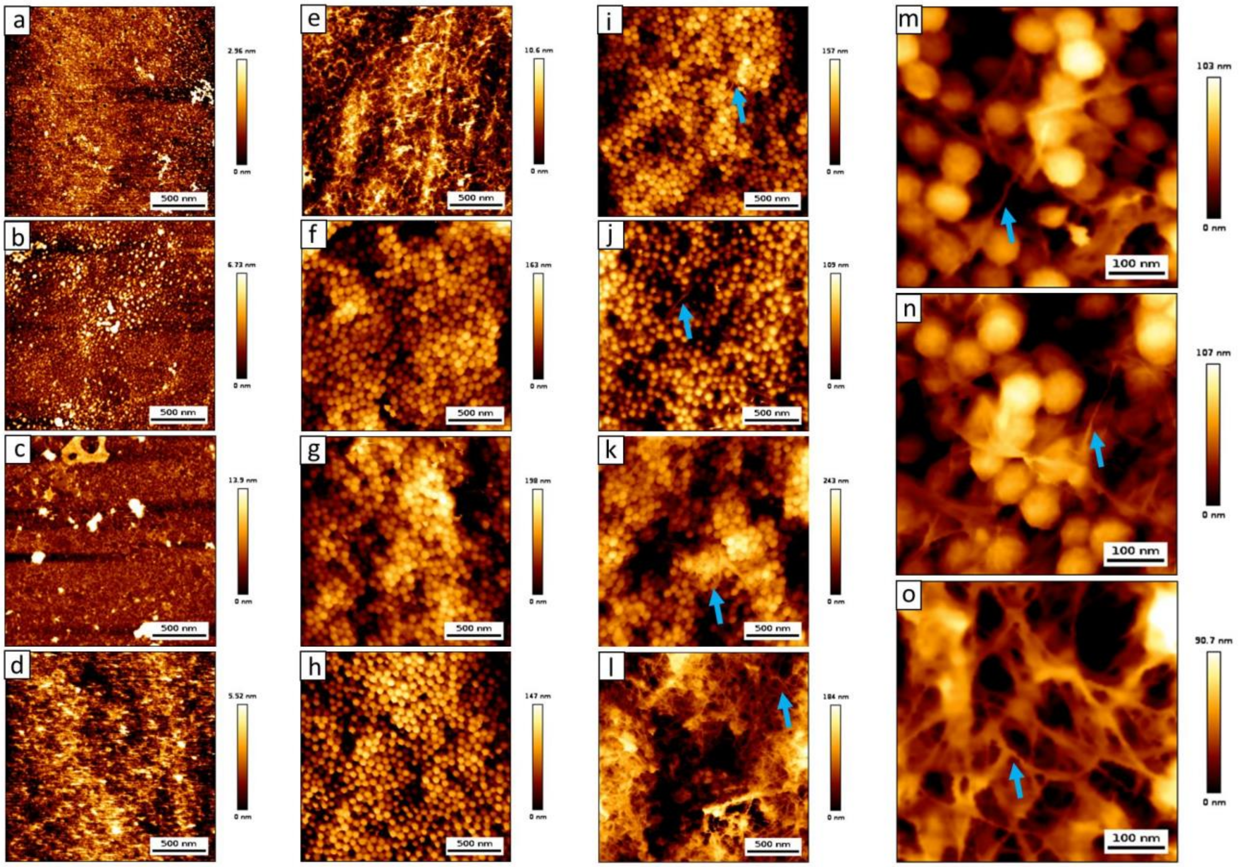

p

Surface	PLL	PLL +G	FN	Matrigel	Geltrex	S100	SC50	SC25	SC12.5	SC6.25	SC2.5	C100
Ra (nm)	0.5 ± 0.0	1.1 ± 0.5	2.4 ± 0.9	1.6 ± 0.6	1.4 ± 0.4	29.7 ± 7.2	33.2 ± 9.0	26.5 ± 3.7	26.5 ± 3.5	23.3 ± 5.6	38.2 ± 5.6	27.8 ± 8.1

Supplementary Figure 16. AFM analysis of dried SiNP/CNT-DNA nanocomposite materials. Representative AFM images of (**a**) PLL, (**b**) PLL+G, (**c**) FN, (**d**) Matrigel, (**e**) Geltrex, (**f**) S100, (**g**) SC50, (**h**) SC25, (**i**, **m**) SC12.5, (**j**) SC6.25, (**k**, **n**) SC2.5, and (**l**, **o**) C100, respectively. The arrows point at CNT features, which are often observable when the SiNP/CNT mass ratio ≤ 12.5. **l**, Quantification of surface roughness (R_a) of the nanocomposite materials. All data are represented by mean ± S.D. of quadruplicate samples. Note that all SiNP/CNT-DNA nanocomposites reveal similar nanoroughness values, whereas the roughness of PLL, PLL+G, FN, Matrigel or Geltrex is negligible ($Ra < 3.0$ nm).

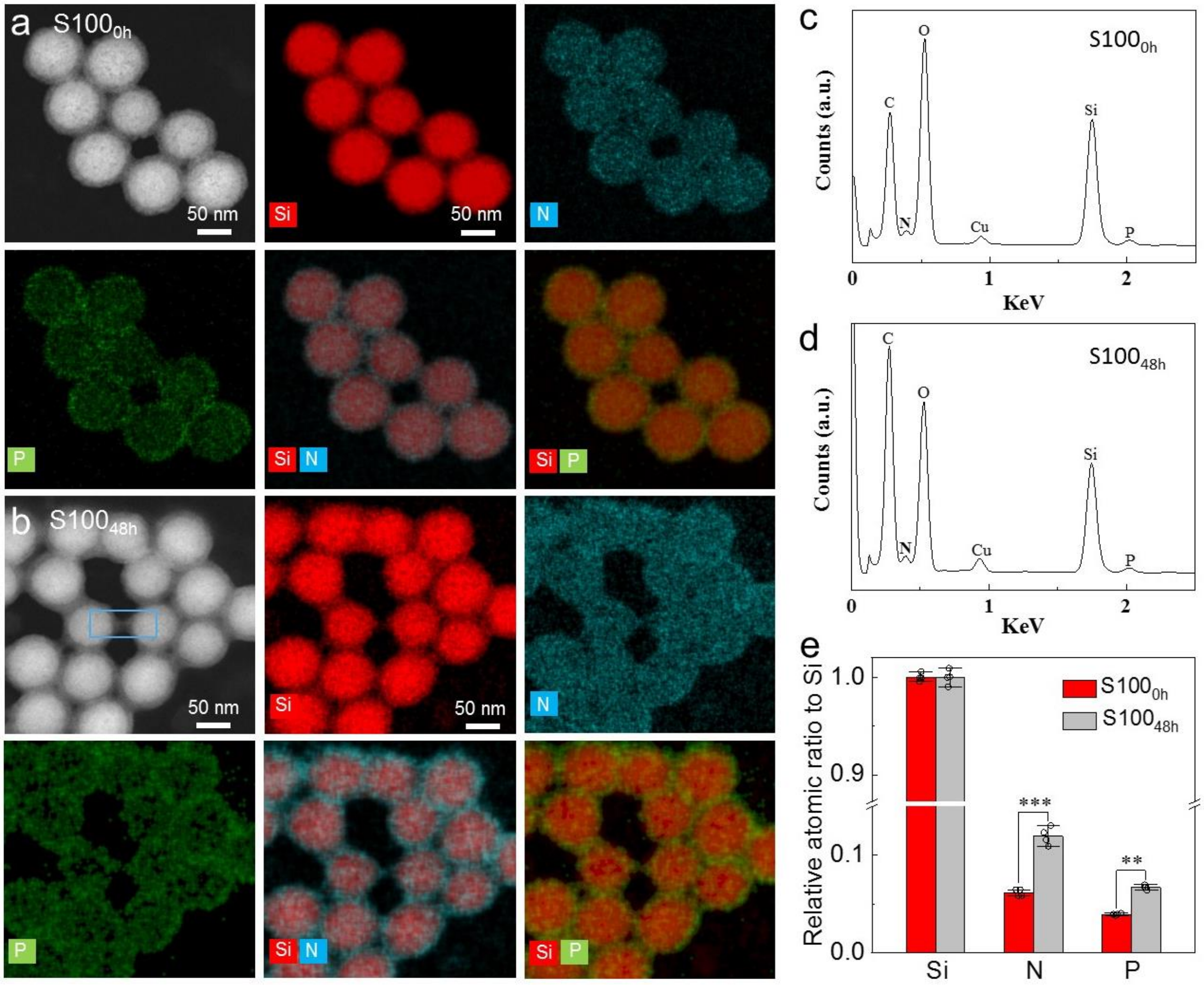

Supplementary Figure 17. Elemental analysis of SiNP-DNA nanocomposite materials. **a**, **b**, Representative HAADF-STEM (TEM operating in the high-angle annular dark-field with scanning) combined with EDS elemental mapping of SiNP-P and S100 images, as shown in Figure 3d, e. Note that the comparison of the structures and compositions of SiNP and S100 illustrates the stereoscopic structure of the samples observed in the SEM images. Comparison of the distribution of nitrogen and phosphorus with silicon nicely illustrates that S100 has much thicker nitrogen and phosphorus shells than SiNP-P. Moreover, only nitrogen and phosphorus are detected in the area of the putative DNA linkage (quadrangle in **b**) in between two particles in S100. **c**, **d**, Corresponding EDS spectra, and (**e**) relative atomic ratios of N and P to Si. Data represent mean ± S.D. of the EDS measurements determined from fifty particles in four random areas. The presence of carbon, nitrogen, oxygen, silicon, and phosphorus in the non-polymerised SiNP-P is due to the multifunctional ligand shell (see Supplementary Figure 1) and the appended ssDNA primer (**c**). The relative average atomic ratios of nitrogen and phosphorus to silicon in S100 is substantially higher than for SiNP-P due to the extension of DNA by RCA reaction (**e**).

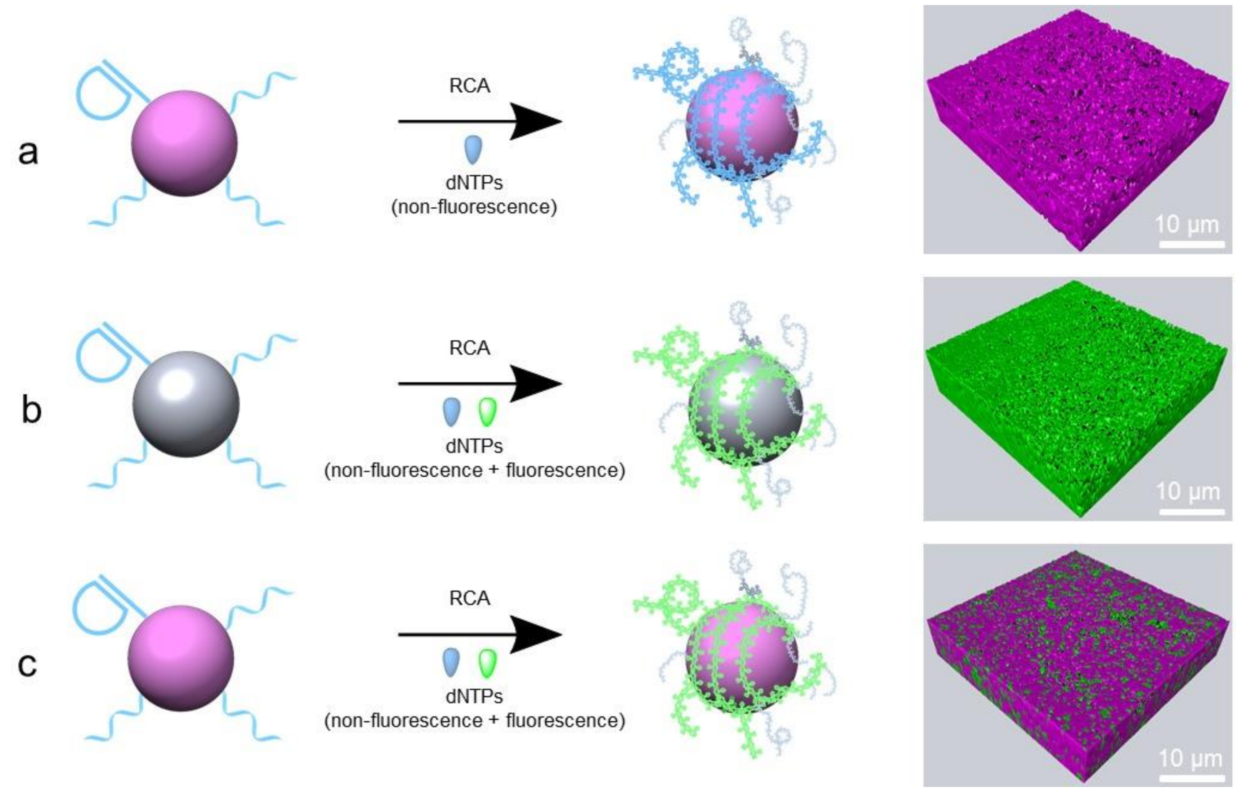

Supplementary Figure 18. Incorporation of fluorescence into SiNP-DNA nanocomposite materials. Incorporation of fluorescent properties into S100 *via* (**a**) the use of Cy5 fluorescently-labeled SiNP, (**b**) enzymatic introduction of chemically modified deoxynucleotides (FITC-dUTP), and (**c**) by using both strategies. The resulting hydrogels were analyzed by confocal fluorescence microscopy. The shown images are 3D reconstructions from z-stack analyses (Cy5 magenta, FITC green).

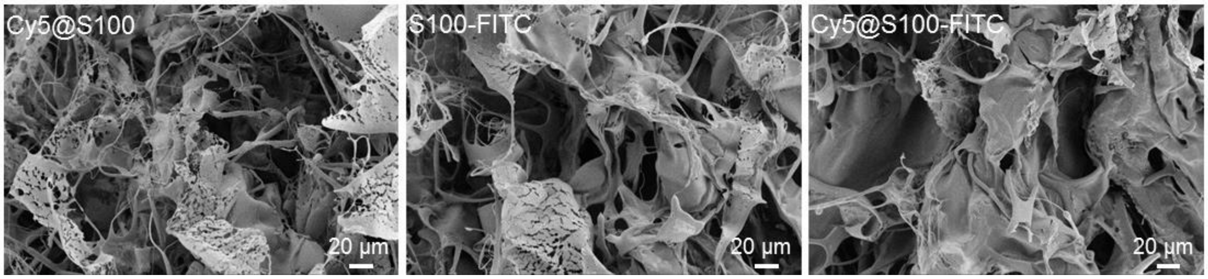

Supplementary Figure 19. SEM analysis of the ultrastructure of fluorescent SiNP-DNA nanocomposite materials. Representative SEM images of Cy5@S100, S100-FITC, and Cy5@S100-FITC, synthesized as described above in Supplementary Figure 18.

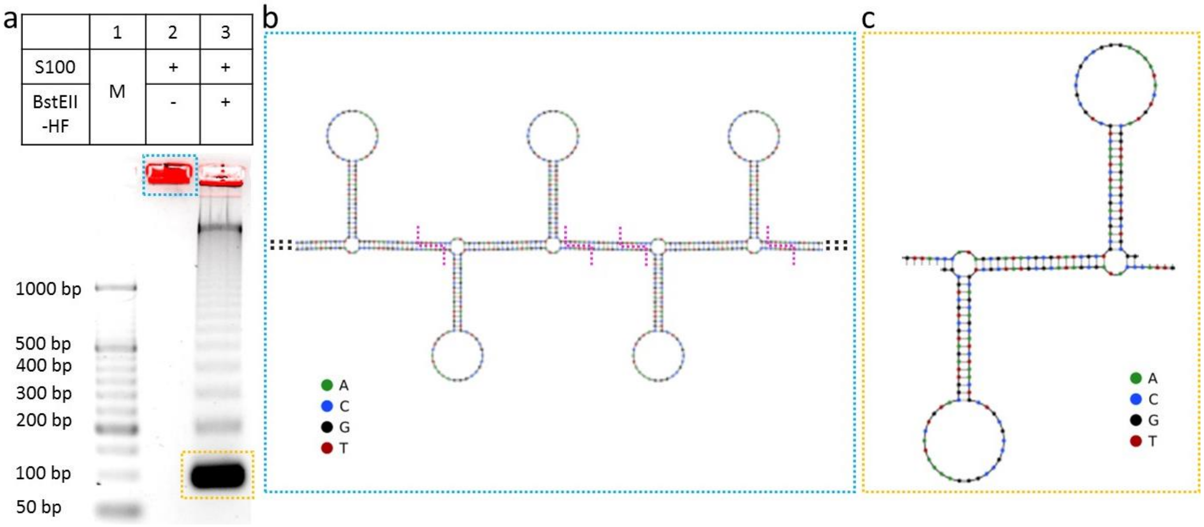

Supplementary Figure 20. Electrophoretic analysis of endonuclease digestion of SiNP-DNA nanocomposite. **a**, Agarose (2.5%) electrophoresis of the products obtained from degradation of S100 with BstEII-HF (15 U mL^{-1}) for 1 h. Note that cleavage of DNA motifs (depicted in **b**) is expected to yield degradation products with an electrophoretic mobility of about 90 bp (predicated fragments in (**c**) contain 174 nucleotides. The bands in lane 3 indicate the expected fragments as monomers and higher oligomers. The specificity of endonuclease digestion was also verified in Supplementary Figure 23. Electrophoreses were carried out in 1× TAE-Mg^{2+} at 120 V for 45 min; the gel was stained with EtBr. The Cy5 fluorescence signals (red) in the gel pocket stem from SiNP. Lane M: DNA marker.

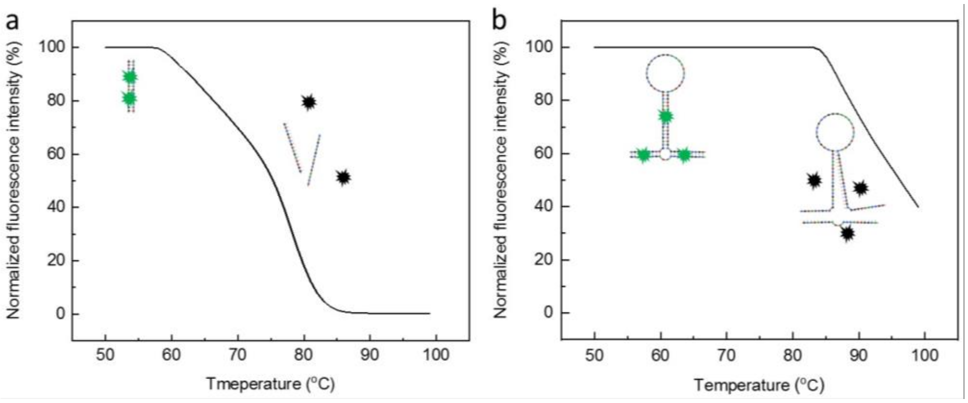

Supplementary Figure 21. Melting curve of SiNP-DNA nanocomposite materials. Melting curve of (**a**) dsDNA (M3/M4, see Supplementary Table 6) and (**b**) S100 stained with the intercalating dye SYBR Green I, which binds with high preference to base-paired regions of the double-stranded DNA backbone. The decrease in fluorescence indicates melting of double-stranded regions inside composites' DNA backbone. Note that, the melting temperature of M3/M4 with 58% GC was estimated to be around 75°C. The higher melting temperature of S100 (56% GC in T-1) ≥ 90 °C suggests an improved thermal stability of the self-assembled DNA strands in the composites.

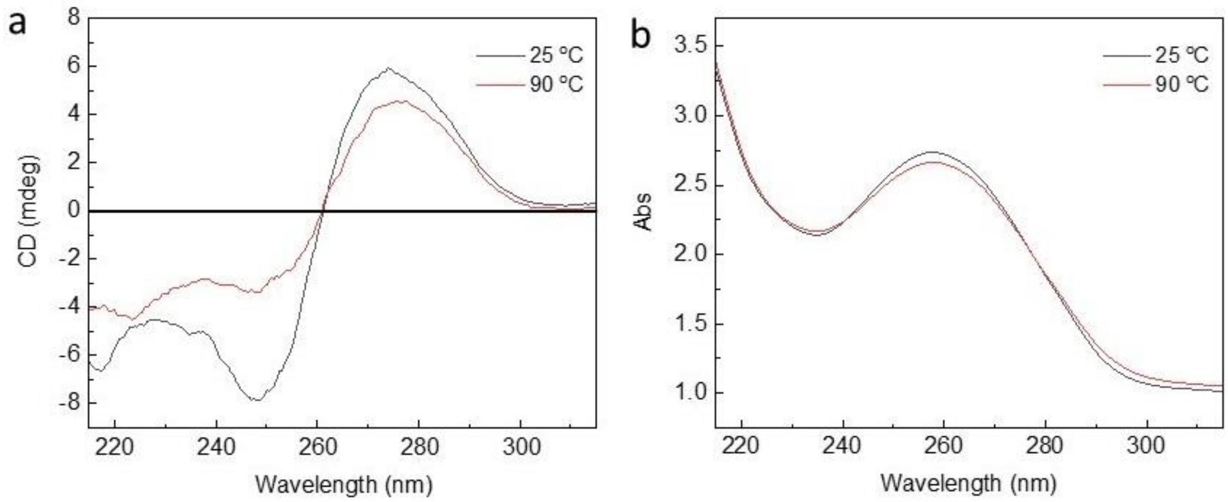

Supplementary Figure 22. The engineered DNA hydrogel backbone shows the typical properties of double-stranded DNA. **a,** Circular dichroism (CD) and (**b**) absorbance spectra of the DNA hydrogel, recorded at 25 °C (black) and 90 °C (red), respectively. Note that the CD spectra of DNA hydrogel backbone shows a positive CD band at a wavelength of around 275 nm, crossing over near 258 nm and a negative band around 246 nm, which is similar to the observation of canonical B-form DNA double helical structure[12]. The changes in ellipticity in CD spectra at high temperature (90 °C) are due to the fact that dsDNA partially dissociates to form ssDNA[13], in agreement with the results of melting experiments (Supplementary Figure 21).

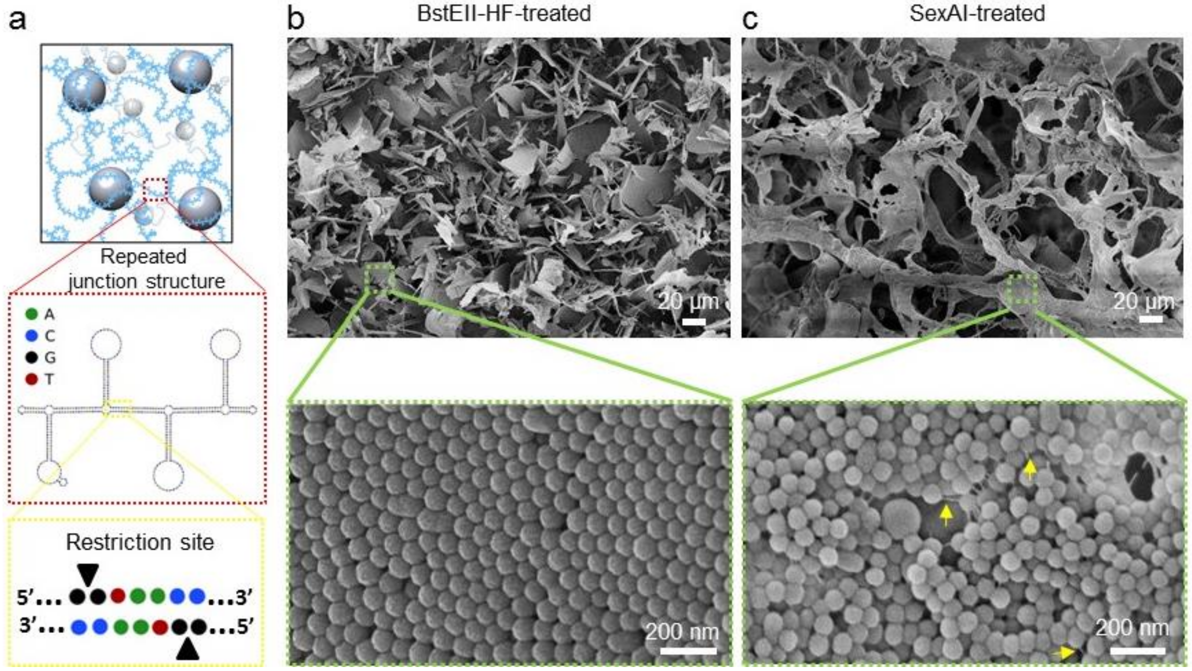

Supplementary Figure 23. Specific endonuclease degradation of S100 bearing a DNA backbone with enzymatic restriction sites. **a,** Predicted secondary structures of RCA products obtained from circular template T, bearing stem-loop structures and double stranded regions that contain BstEII restriction sites (5'-GAATTC-3') in a repeated junction structure. SEM images of S100 treated

for 2 h (37 °C) with restriction enzymes BstEII-HF (**b**) or, as a control, with SexAI (**c**). The yellow arrows point at representative DNA linkers between particles. Note that enzymatic digestion leads to particle assemblies (**b**) similar as obtained from unpolymerised SiNP (Supplementary Figure 12), whereas treatment with the non-corresponding restriction endonuclease SexAI does not change the gel's morphology (**c**). Note that the specific endonuclease digestion was also verified in Supplementary Figure 20**.**

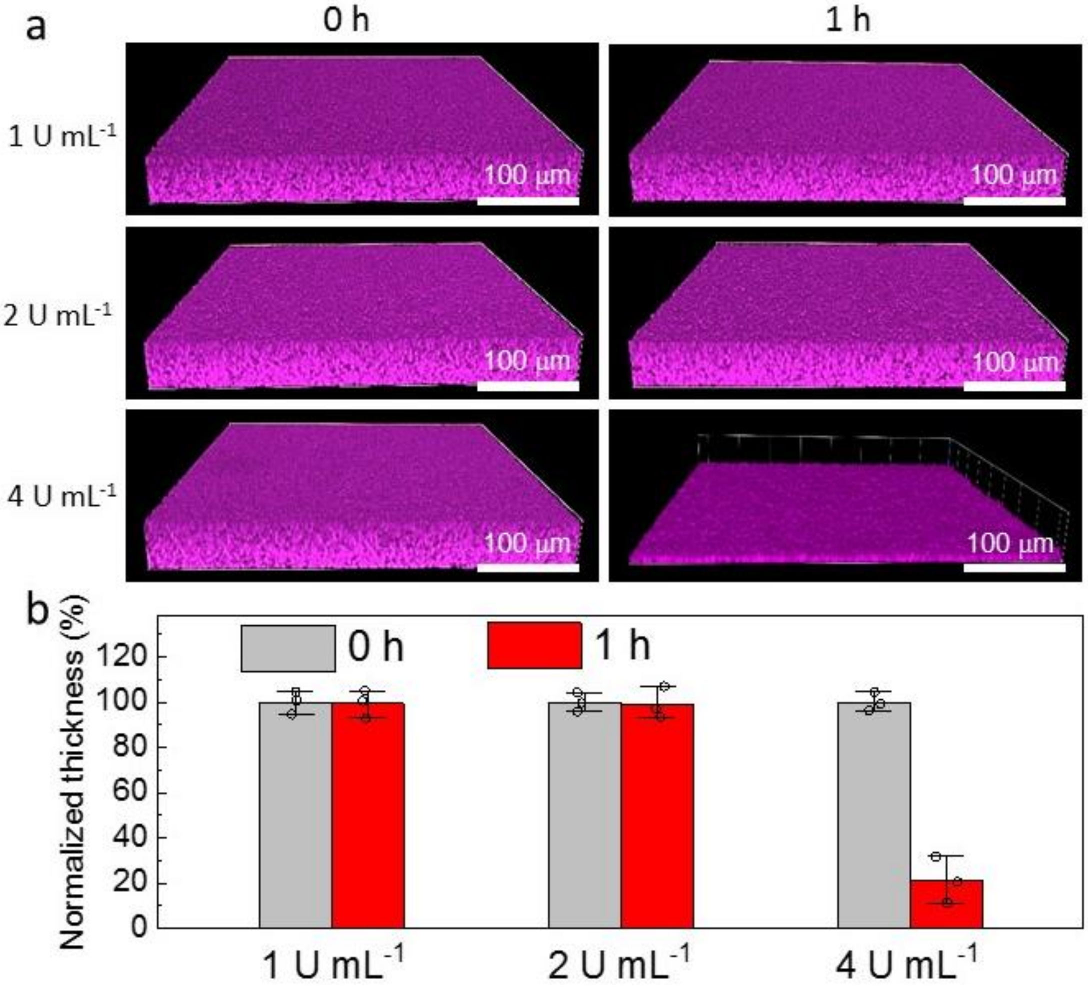

Supplementary Figure 24. Stability of SiNP-DNA nanocomposite material S100 against non-specific digestion by DNase I. S100, prepared in glass bottom 96-well plates, was rinsed with distilled water and immersed in solutions containing variable amounts of DNase I (1-4 U mL^{-1}) in 1x DNase I reaction buffer (10 mM Tris-HCl, 2.5 mM $MgCl_2$, 0.5 mM $CaCl_2$, pH 7.6). Analysis was conducted by confocal microscopy imaging (**a**). Average layer thickness is represented as bars in (**b**). Note that the SiNP-DNA nanocomposite shows good stability against non-specific degradation by DNase I at concentrations as high as 2 U mL^{-1}, as previously described for pure DNA hydrogels[14].

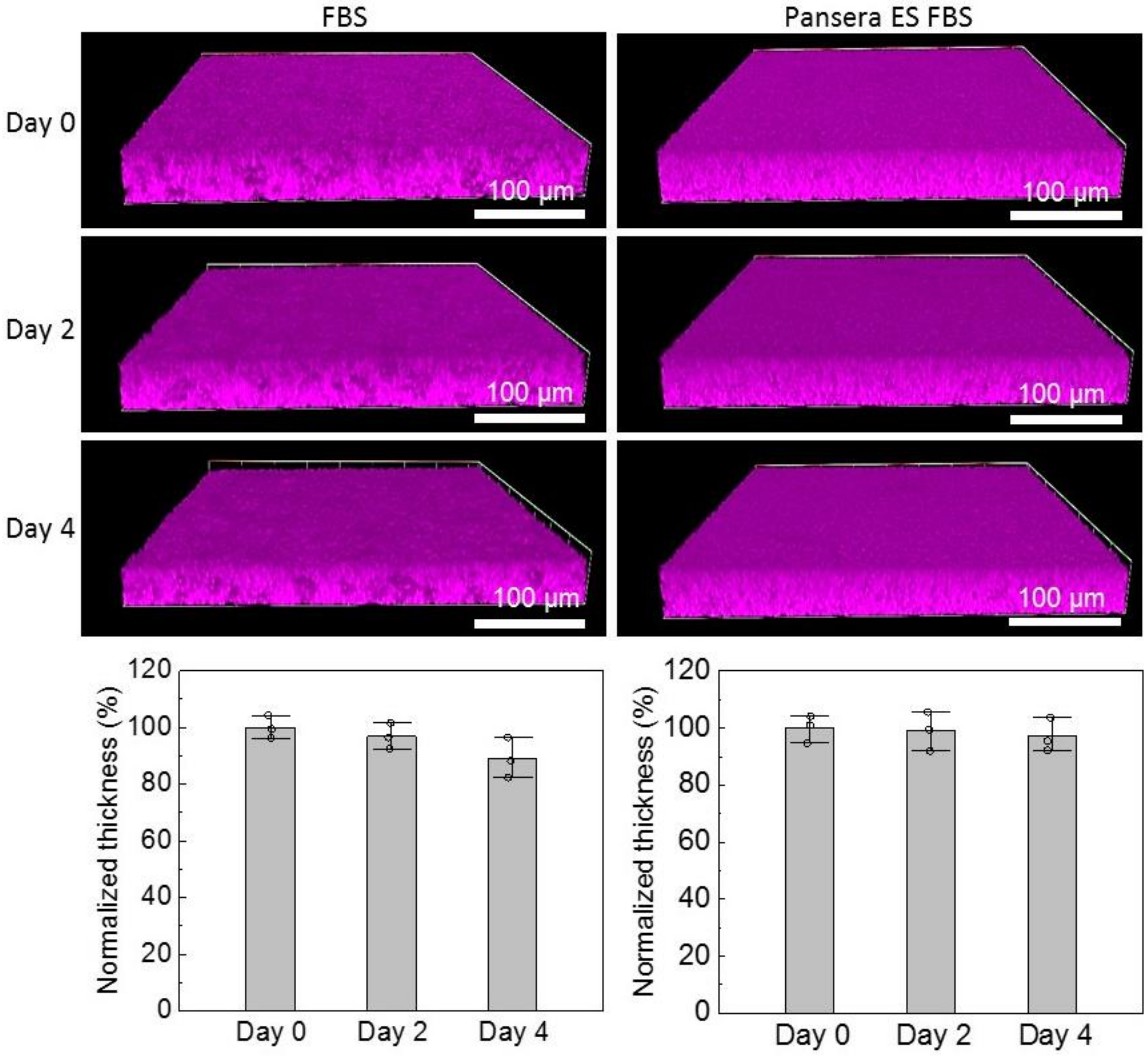

Supplementary Figure 25. Long-term stability of nanocomposite material S100 in media. S100 was prepared in glass bottom 96-well plates, rinsed by distilled water, immersed in 10% FBS medium or Pansera ES FBS medium and analysed by confocal microscopy at the given time points. Note that the SiNP-DNA nanocomposite layers lost about 10% in thickness after 4 days exposure to FBS, indicating a good stability in FBS. An even better stability was observed against Pansera ES FBS medium which led to only 3% reduction in thickness.

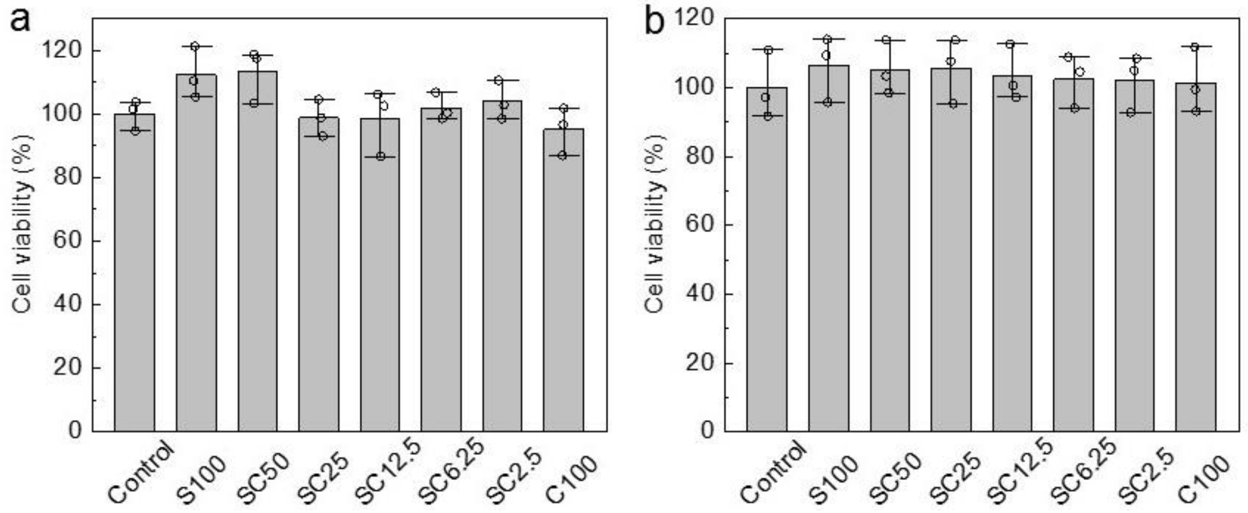

Supplementary Figure 26. Cytocompatibility of SiNP/CNT-DNA nanocomposite materials. CCK-8 viability assay after incubation of (**a**) MCF7 cells and (**b**) REF52 cells for 24 h with 75 μL of the various nanocomposite materials or, for control, with DPBS buffer only.

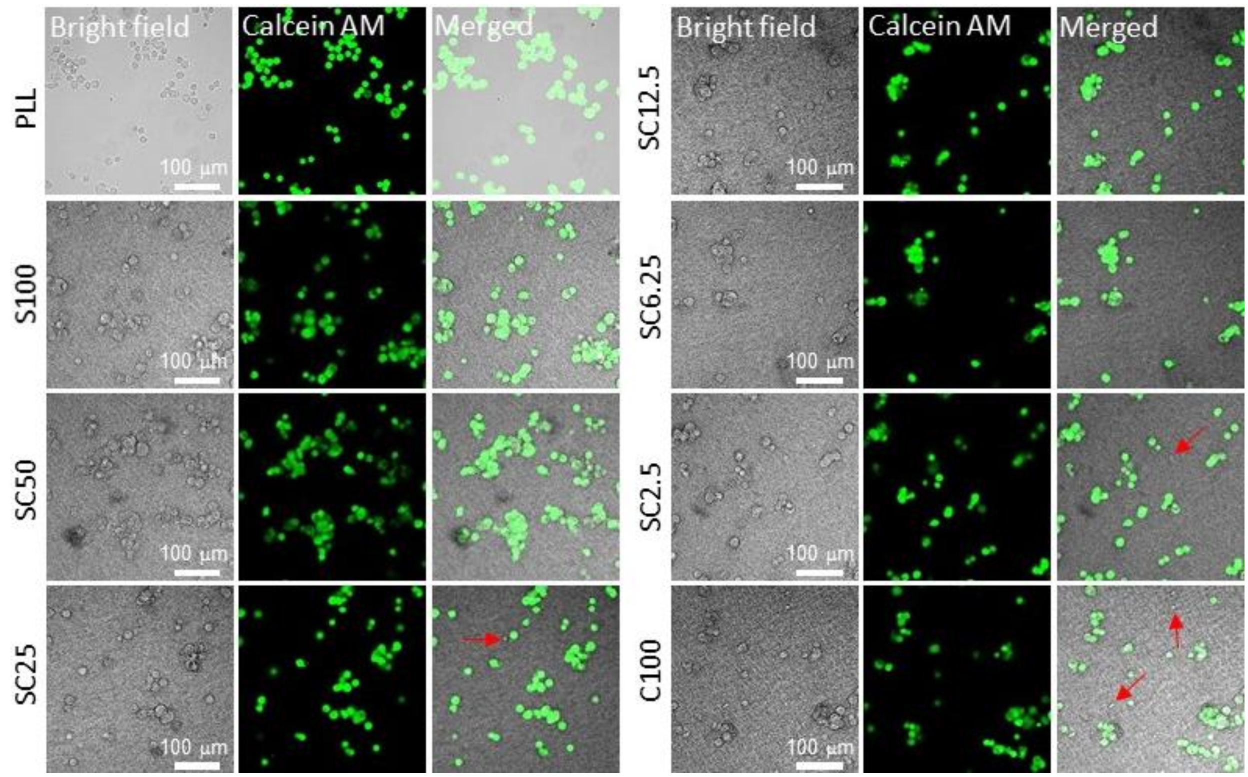

Supplementary Figure 27. Live cell staining of MCF7 cells grown on fresh SiNP/CNT-DNA nanocomposite materials. After cultivation of cells on the materials for 24 h, MCF7 cells were stained with Calcein AM. The arrow points at some dead cells that are indicated by the lack of Calcein AM staining. Note that, only very few dead cells were found on the nanocomposite materials.

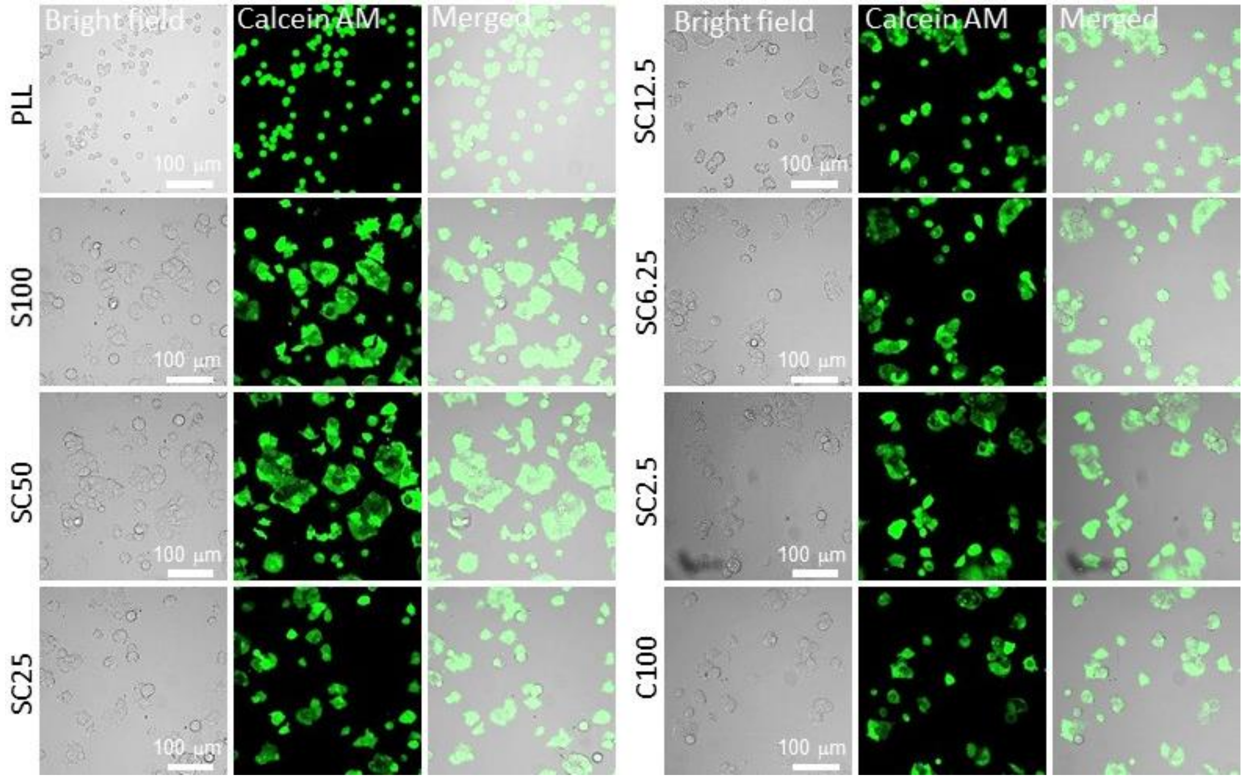

Supplementary Figure 28. Live cell staining of MCF7 cells grown on dried SiNP/CNT-DNA nanocomposite materials. After cultivation of cells on the dried materials for 24 h, MCF7 cells were stained with Calcein AM and then analyzed by confocal microscopy (LSM 880). Note that no dead cells were found on the nanocomposite materials.

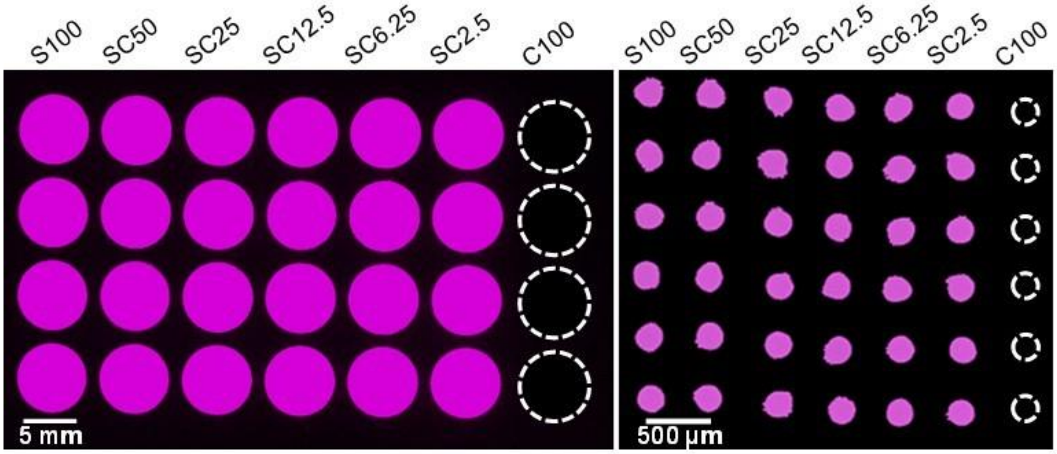

Supplementary Figure 29. Production of libraries of SiNP/CNT-DNA nanocomposite materials. Fluorescence image of an array of Cy5-labeled binary and ternary SiNP/CNT-DNA nanocomposite materials. Samples of the various materials were prepared directly in the wells of a 96 well glass bottom plate by adding 75 µL of the reaction mixtures (left) or on chemically-activated glass slides *via* ink-jet spotting (right) to facilitate RCA synthesis. For the latter, 0.6 nL per spot of each reaction mixture were deposited onto PBAG (Poly(Bisphenol A-*co*-epichlorohydrin)-activated glass slides with a piezo-driven spotter (NP 1.2, GESIM, Dresden), surface modification and spotting procedures were adopted from the previous report[15].

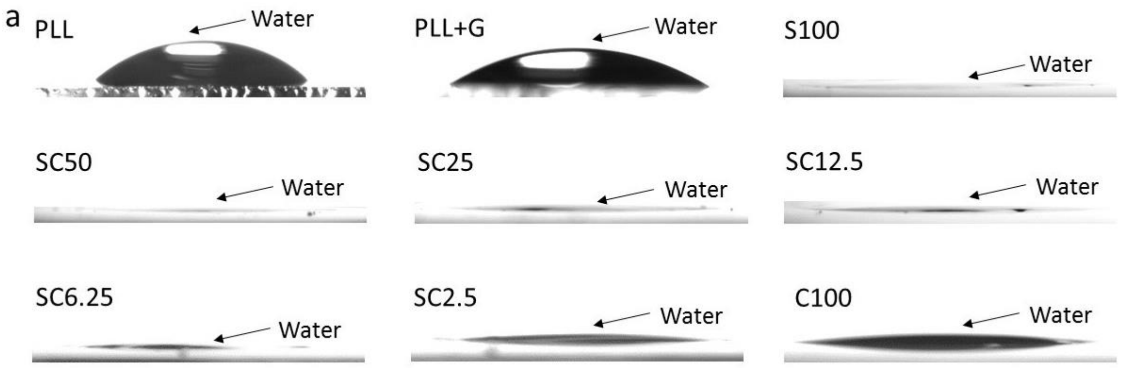

b

Surface	PLL	PLL+G	S100	SC50	SC25	SC12.5	SC6.25	SC2.5	C100
θ	45.7 ± 3.3°	34.2 ± 3.0°	~0°	~0°	~0°	~0°	3.6 ± 1.2°	4.0 ± 1.9°	9.7 ± 1.9°

Supplementary Figure 30. Static water contact angle (WCA) analysis of dried SiNP/CNT-DNA nanocomposite surfaces. **a**, Images of water drops on the various surfaces and (**b**) quantified WCA values of the corresponding surfaces. All data are represented by mean ± S.D. of triplicate samples. Note that, the PLL surface after coating with gelatin displayed increased hydrophilicity (45.7° for PLL *vs* 34.2° for PLL+G). S100, SC50, SC25, SC12.5, SC6.25, and SC2.5 have superhydrophilic surfaces ($\theta < 5°$, roughness factor $r > 1$)[16], whereas an increased CNT ratio in the nanocomposite materials leads to the gradual increase in surface hydrophobicity. However, even C100 is substantially less hydrophobic than PLL and PLL+G.

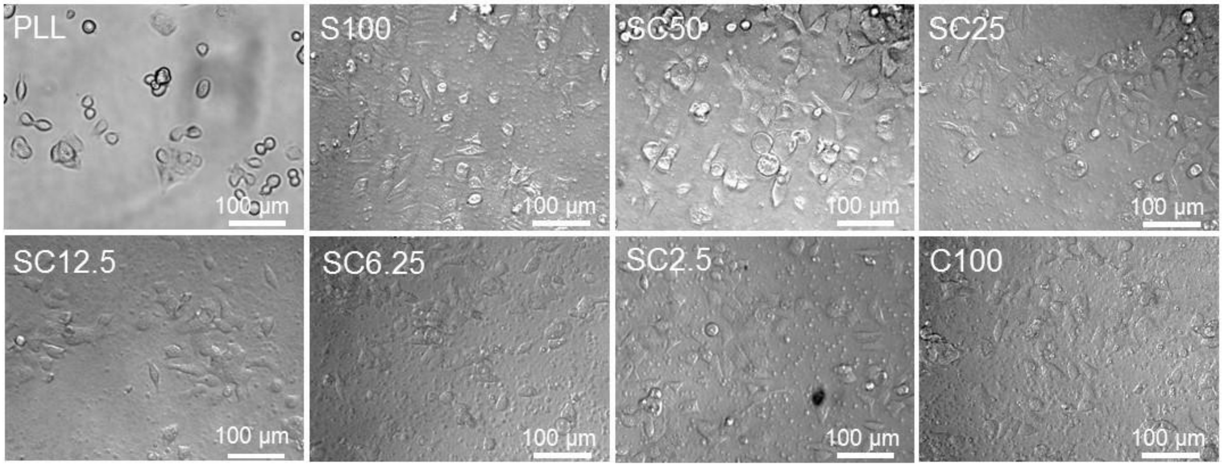

Supplementary Figure 31. Bright field imaging analysis of MCF7 cells adhered on dried SiNP/CNT-DNA nanocomposite materials. Transmitted light inverted microscopy (EVOS™ FL Imaging System) analysis of MCF7 cells adhered for 24 h on the various surfaces of dried hydrogels or, for control, on PLL surface. Note that cells on S100 and SCx surfaces reveal a more pronounced elongated fusiform and flattened morphology than cells adhered on PLL surface.

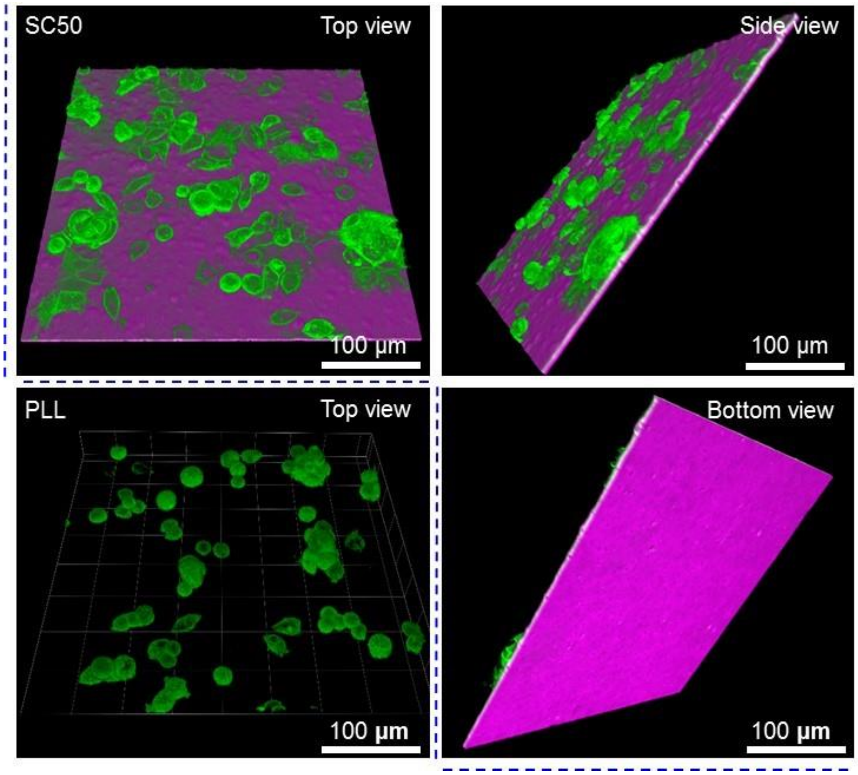

Supplementary Figure 32. Fluorescence microscopy analysis of $MCF7_{eGFP}$ cells adhered on SC50. Representative fluorescence microscopy images of $MCF7_{eGFP}$ cells cultivated on dried SC50 film prepared from Cy5-labeled SiNP as well as cells on PLL surface. The bottom view image shows that the cells have not transmigrated through the dried gel matrix. The shown images are 3D reconstructions from z-stack analyses (Cy5 magenta, GFP green).

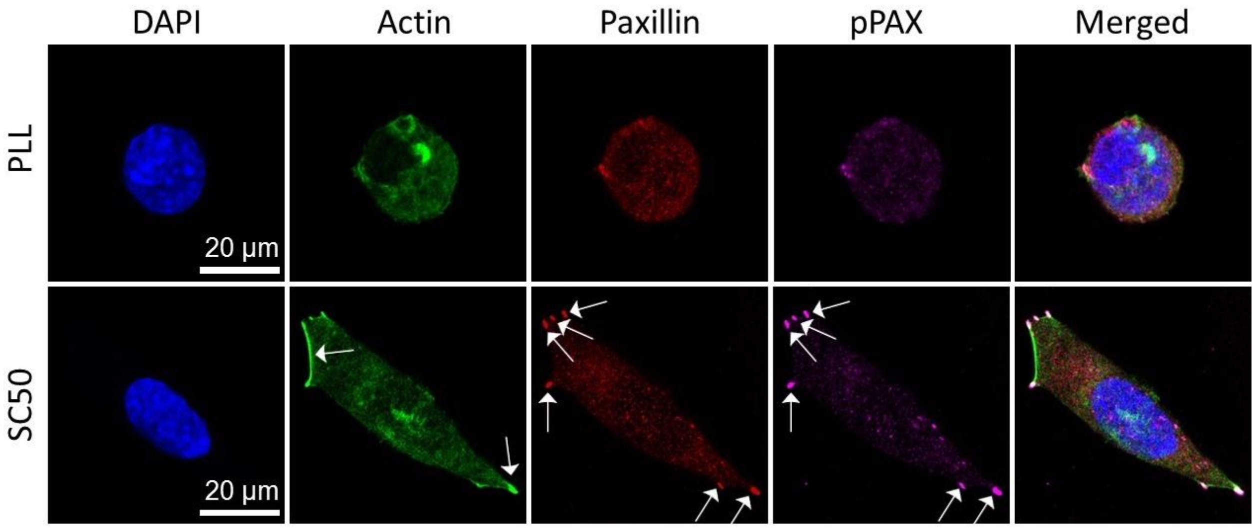

Supplementary Figure 33. Phalloidin staining of actin cytoskeleton and immunostaining of Paxillin and pPAX of MCF7 cells grown on dried SC50. After cultivation on PLL or dried SC50 for 24 h, MCF7 cells were stained with Alexa Fluor®488 phalloidin and immunostained with primary monoclonal antibodies (mAb) IgG mouse anti-Paxillin and polyclonal (pAb) IgG rabbit anti-Phospho-Paxillin (pPAX) pTyr31 and secondary antibodies (goat anti-mouse IgG Cy3 and goat anti-rabbit IgG Alexa Fluor®647). The white arrows point at the actin cytoskeleton, paxillin clusters, or pPAX clusters. Paxillin clusters in the actin cytoskeleton are a marker for integrin positive cell-matrix adhesion sites[17], and pPAX clusters are a marker for activated integrin signaling sites[18]. Note that these markers of focal adhesions are not well formed in cells grown on PLL, whereas their clear appearance in MCF7 cells on SC50 surfaces indicates the successful formation of focal adhesion sites.

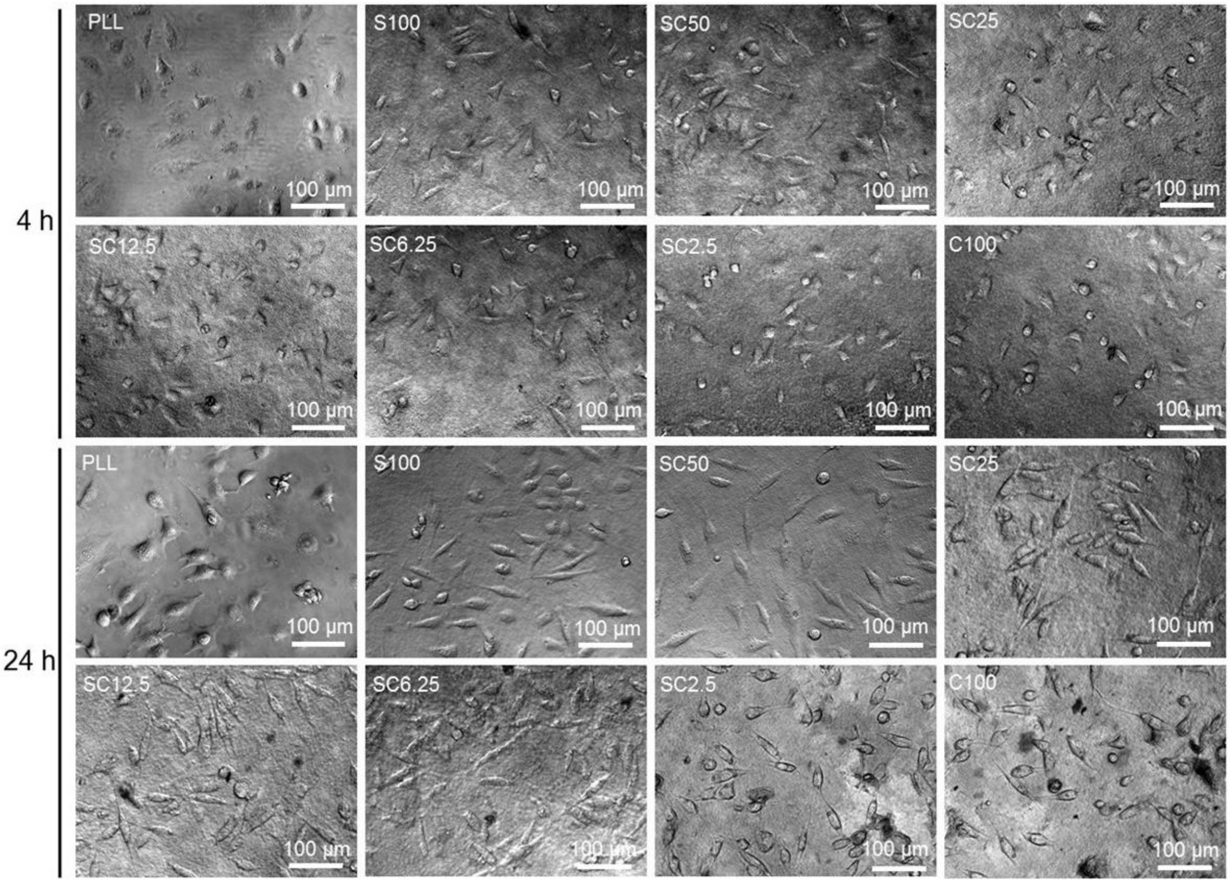

Supplementary Figure 34. Bright field imaging analysis of REF52 cells adhered on dried SiNP/CNT-DNA nanocomposite materials. Transmitted light inverted microscopy (EVOS™ FL Imaging System) images of REF52 cells adhered onto the various dried hydrogels and PLL surfaces for 4 h and 24 h. Note that REF52 cells, unlike MCF7 cells (Supplementary Figure 31), do not exhibit a substantially more pronounced, elongated fusiform and flattened morphology on the S100 and SCx surfaces than cells adhering to the PLL surface. We attribute this observation to the well known fact that REF52 cells are strongly adhesive and are capable of adhering and spreading on the surfaces of a broad range of materials[19, 20]. On the SCx surfaces, the REF52 cells only reveal a slightly more elongated fusiform and flattened morphology as compared to cells adhered on the PLL substrate.

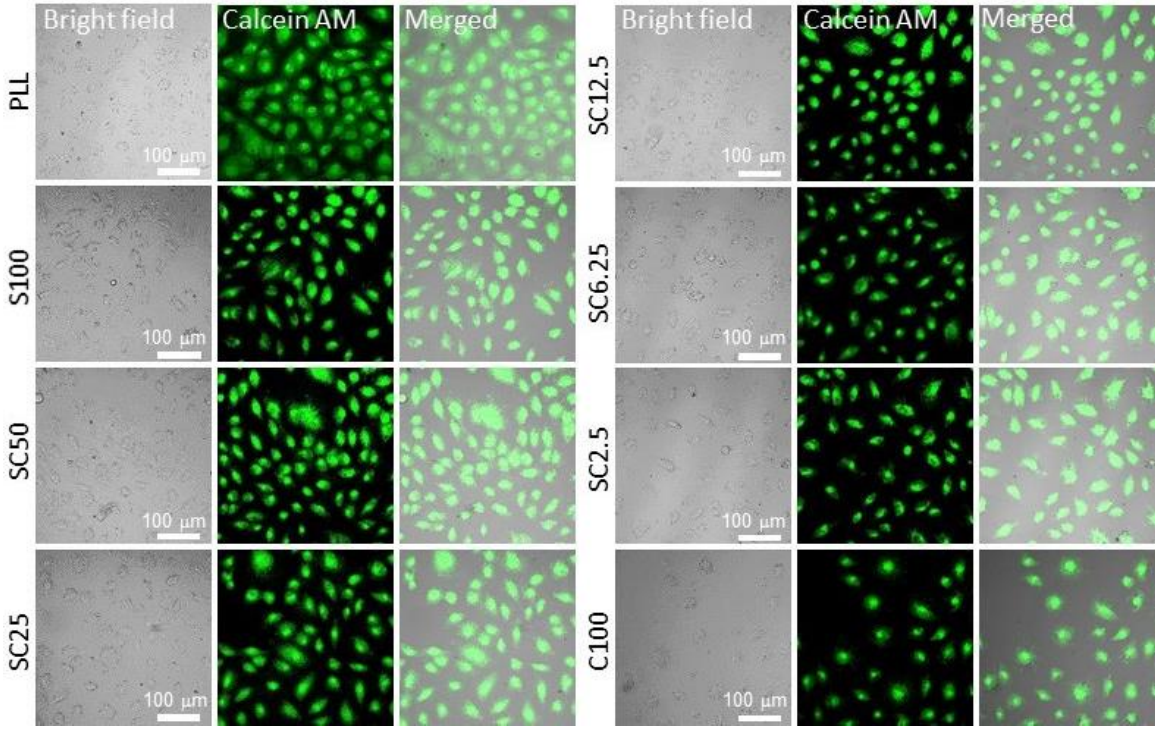

Supplementary Figure 35. Live cell staining of REF52 cells grown on dried SiNP/CNT-DNA nanocomposite materials. After cultivation of cells on the dried materials for 24 h, REF52 cells were stained with Calcein AM and then analyzed by confocal microscopy (LSM 880). Note that no dead cells were found on the nanocomposite materials.

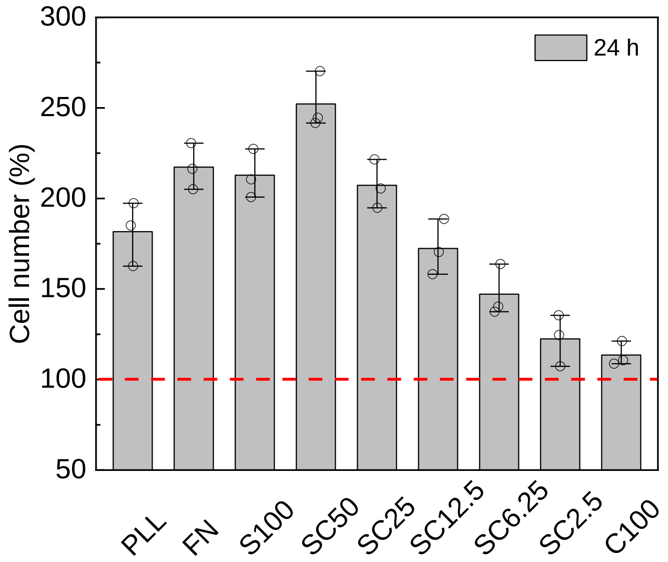

Supplementary Figure 36. Proliferation rate of REF52 cells grown on dried SiNP/CNT-DNA nanocomposite materials. Quantification of REF52 cell proliferation was achieved by using the CCK-8 assay. The blue bars indicate relative cell numbers after 24 h normalized to initially seeded cells (4 h, red dashed line). PLL and FN data were obtained from polylysine- and fibronectin-coated glass surfaces, respectively, used as controls.

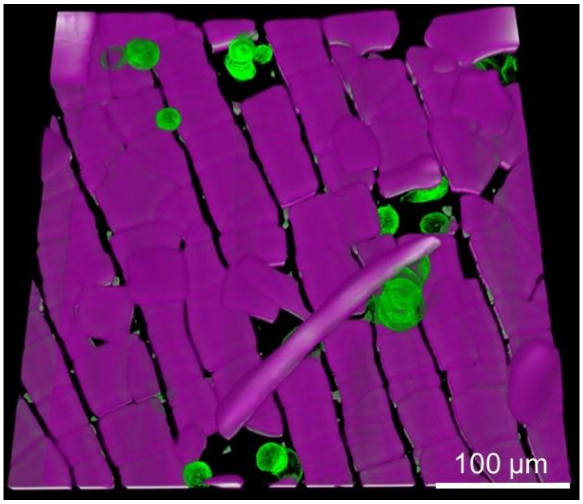

Supplementary Figure 37. Fluorescence microscopy analysis of $MCF7_{eGFP}$ cells cultivated on dried SiNP-P. Fluorescence microscopy analysis of $MCF7_{eGFP}$ cells cultivated for 24 h on the dried Cy5-labeled SiNP-P (Cy5 magenta, GFP green). These are control experiments with SiNP-P that were not subjected to RCA polymerisation. Note that the particles dried into thin layers of cracked particle blocks to which $MCF7_{eGFP}$ cells predominantly adhere at the blocks' edges in a round morphology.

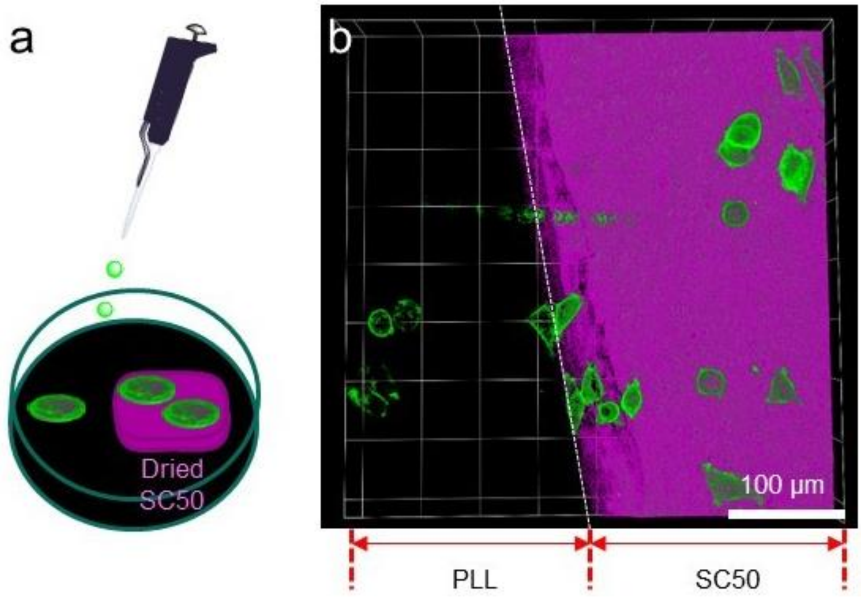

Supplementary Figure 38. Competitive adhesion of $MCF7_{eGFP}$ cells to either PLL or dried SC50. A PLL surface containing a patch of dried SC50 (magenta) was immersed in 1 mL medium containing 1.2×10^4 $MCF7_{eGFP}$ cells (green). **a**, Schematic drawing of the experiment. **b**, Fluorescence microscopy image of the border region between PLL and SC50 surfaces.

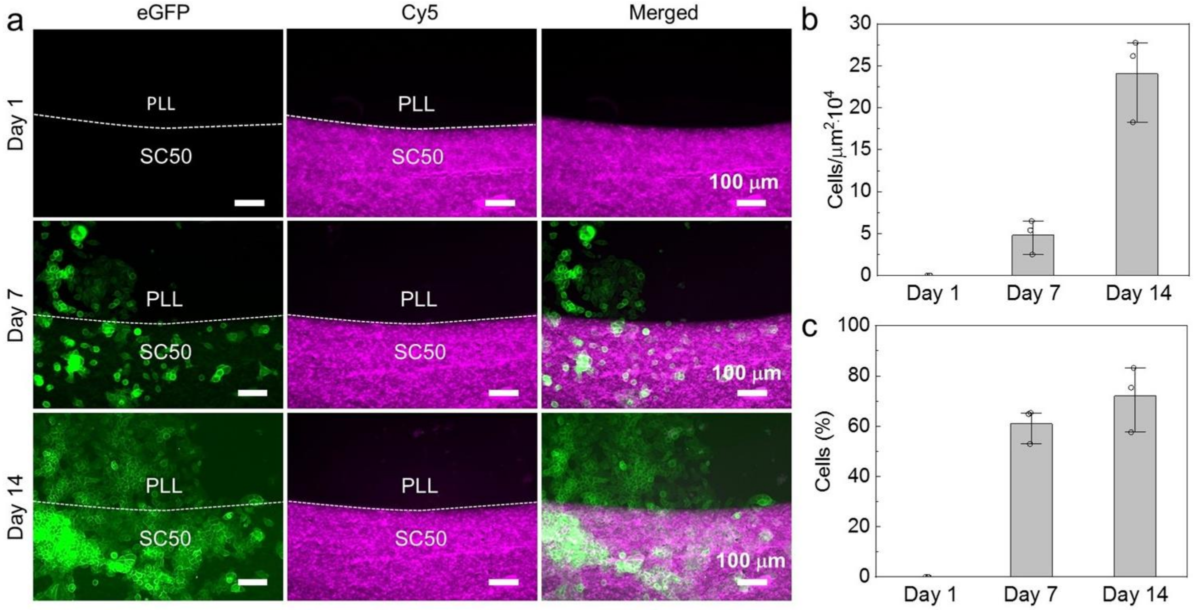

Supplementary Figure 39. Directional lateral migration of $MCF7_{eGFP}$ cells. **a**, Directional lateral migration of $MCF7_{eGFP}$ cells that were seeded in a petri dish on PLL surface and allowed to migrate to a patch of Cy5-labeled dried SC50 (magenta). The fluorescence microscopy images of the border region between PLL and SC50 surfaces illustrate the gradual migration of cells from PLL to SC50. **b**, **c**, Quantification of cells migrated to SC50. All data are represented by mean ± S.D. of triplicate samples.

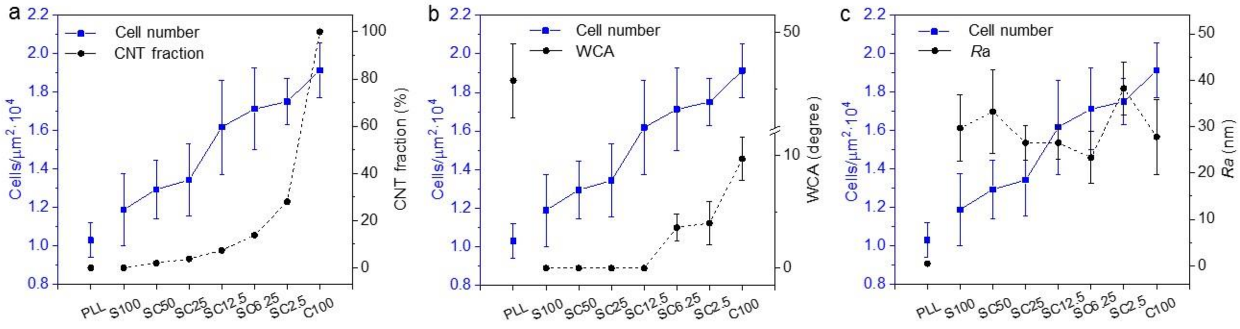

Supplementary Figure 40. Relationship between CNT fraction, water contact angle, surface roughness and cell adhesion. Relationship of (**a**) CNT fraction, (**b**) water contact angle (WCA, corresponding to materials hydrophobicity), and (**c**) surface roughness (*Ra*) with the number of adhered cells remaining after thorough washing (see blue bars in Figure 4e, main manuscript).

Discussion

The numbers of adhered MCF7 cells that remain attached to dried SCx materials after washing is proportional to adhesion strength of the cells (see blue bars in **Figure 4e**, main manuscript). This cell number was plotted against the specific material properties of the dried SCx materials with respect to fraction increase of CNT (Supplementary Figure 40a), hydrophobicity (Supplementary Figure 40b) and nano-roughness (Supplementary Figure 40c). The data shows a continuous increase in cell adhesion for the first two aspects, whereas nano-roughness seems to have a marginal effect within the S100-C100 series. Hence, this data suggests that the increasing hydrophobicity in the series of SCx materials has a major effect on cell adhesion.

However, this trend is not followed in the case of PLL, where a much higher hydrophobicity does not lead to improved cell adhesion (Supplementary Figure 40b). PLL also has a much lower nano-roughness than the SCx materials (Supplementary Figure 40c). Therefore, the different cell behaviour observed in our case on S100 and PLL can not only be attributed to these two aspects, but further physicochemical properties of the composites of SiNP, CNT and DNA have to be taken into account. In fact, it is well established that cell behaviour on/in different materials is affected by a variety of factors, such as stiffness, elasticity, wettability, roughness and chemical composition[21-23].

Of note, our data indicates that that proliferation activity on the dried SCx materials correlates with adhesion strength (grey and blue bars, respectively, in Figure 4e, main manuscript). This is in general agreement with previous work indicating that highest motility and proliferation occur at an intermediate adhesion strength, whereas strong adhesion impairs these cellular processes[24, 25].

Also note that the cell growth on the fully hydrated, fresh SCx materials (Figure 4f, main manuscript) differs from that on dried SCx materials (Figure 4d,e), as the transmigration ability of the cells must also be taken into account.

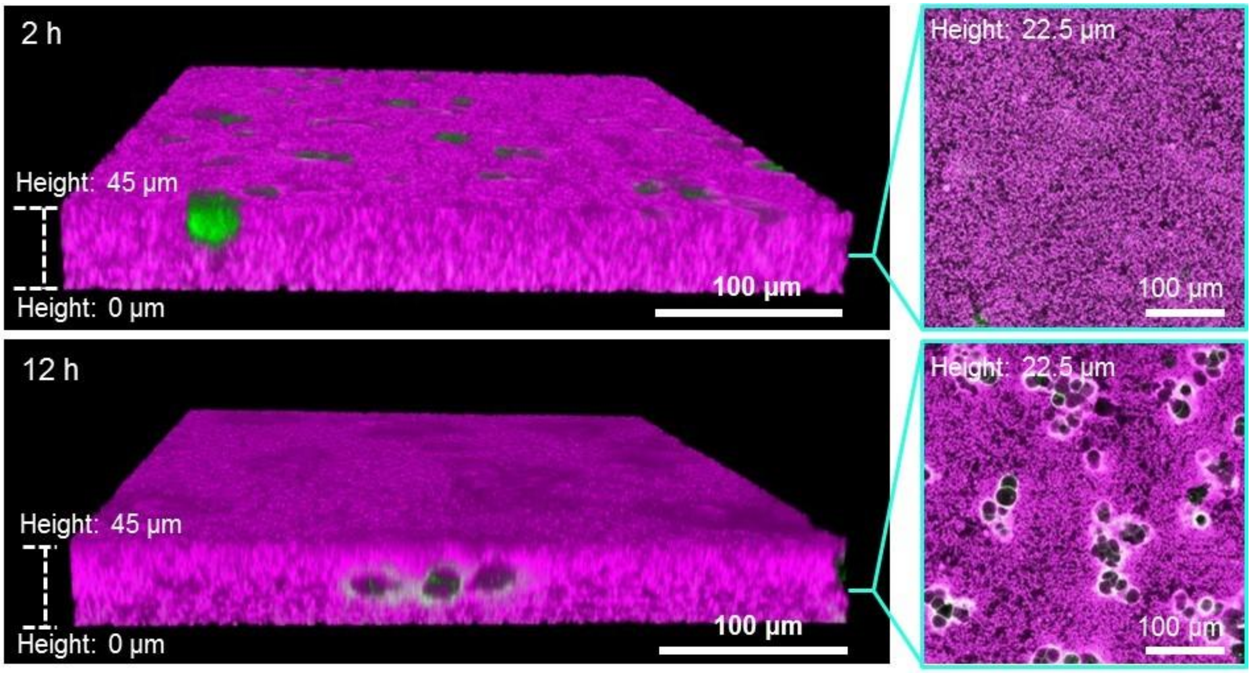

Supplementary Figure 41. Vertical transmigration of $MCF7_{eGFP}$ into fresh SC50. Fluorescence images of $MCF7_{eGFP}$ cells (green) cultivated on SC50 (magenta) for 2 h and 12 h, respectively. For statistical analysis, see Supplementary Figure 42. Note that cells migrated inside the SC50 at the speed of about 2 μm h^{-1}, whereas cells cannot migrate into SC25 (Supplementary Figure 43).

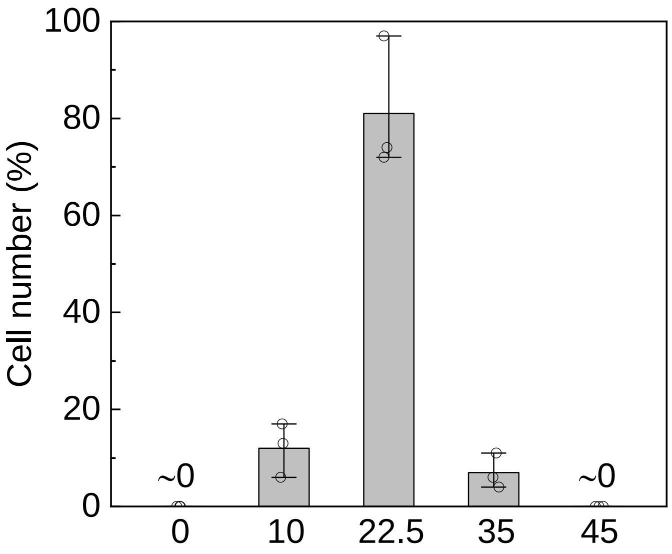

Supplementary Figure 42. Distribution of $MCF7_{eGFP}$ cells transmigrated into fresh SC50. Cell numbers were obtained from fluorescence images of $MCF7_{eGFP}$ cells that were cultivated on SC50 (~45 μm thick) for 12 h on fresh SC50. Note that, more than 80% cells migrated to the central layer of SC50, indicating the homogenous cell distribution in 3D SC50. All data are represented by mean ± S.D. of triplicate samples.

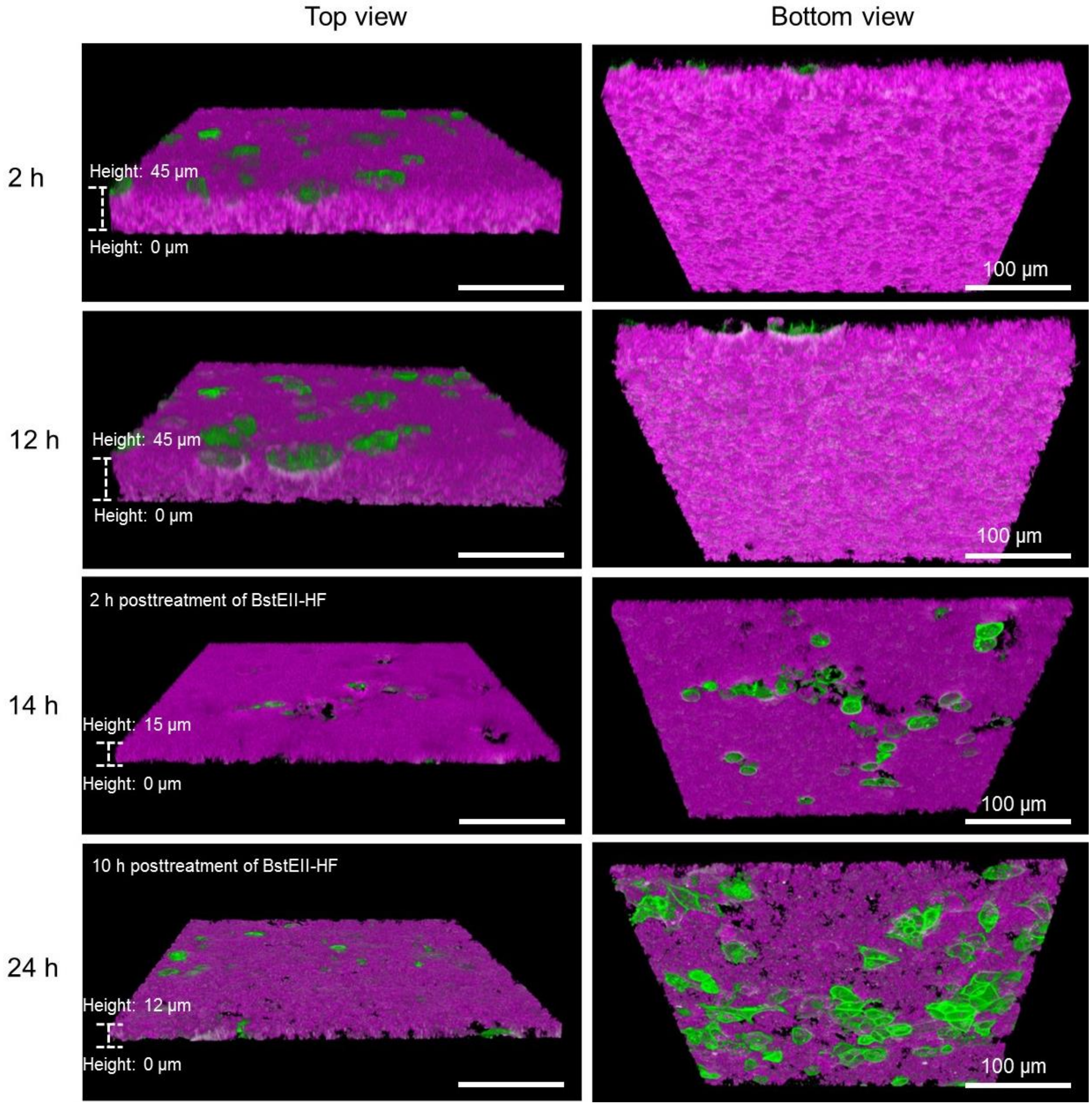

Supplementary Figure 43. Trapping and release of $MCF7_{eGFP}$ cells using fresh SC25. Fluorescence images of $MCF7_{eGFP}$ cells (green) cultivated on SC25 (magenta) for 2 h and 12 h reveal that the cells do not transmigrate but rather dig themselves into the upper layer of SC25. After treatment with restriction endonuclease BstEII-HF for 2 h and an additional 10 h growth period (cultivation times of 14 h and 24 h, respectively), the hydrogel had collapsed in thickness from 45 μm to 15 μm and the cells were able to penetrate the matrix because the DNA backbone was cleaved by the enzyme. Note that after 24 h the released cells have further spread and proliferated on top of the underlying PLL surface. For quantitative analysis, see Supplementary Figure 44.

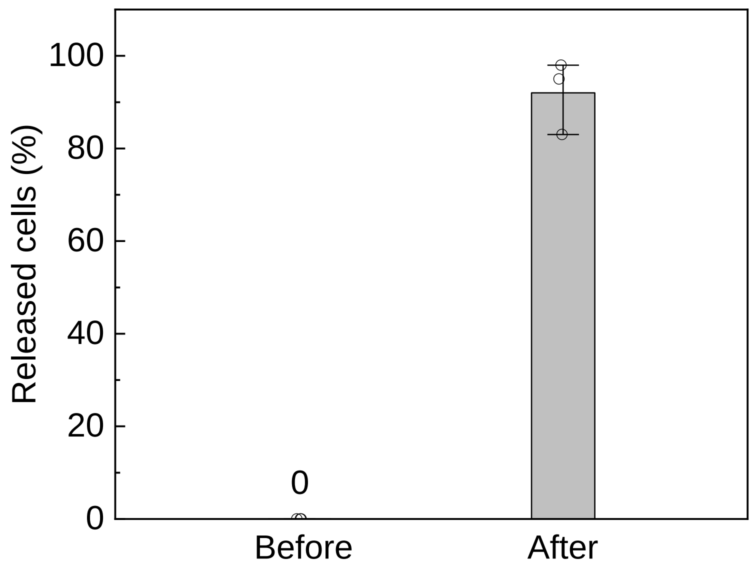

Supplementary Figure 44. Quantification of $MCF7_{eGFP}$ cells located on the glass bottom underneath the SC25 layer, determined before and after endonuclease digestion. The data was determined by statistical analysis of confocal microscopy images, like the ones shown in Supplementary Figure 43 at time points of 12h (before) and 24h (after endonuclease treatment). Note that more than 90% of cells are released due to degradation by endonuclease.

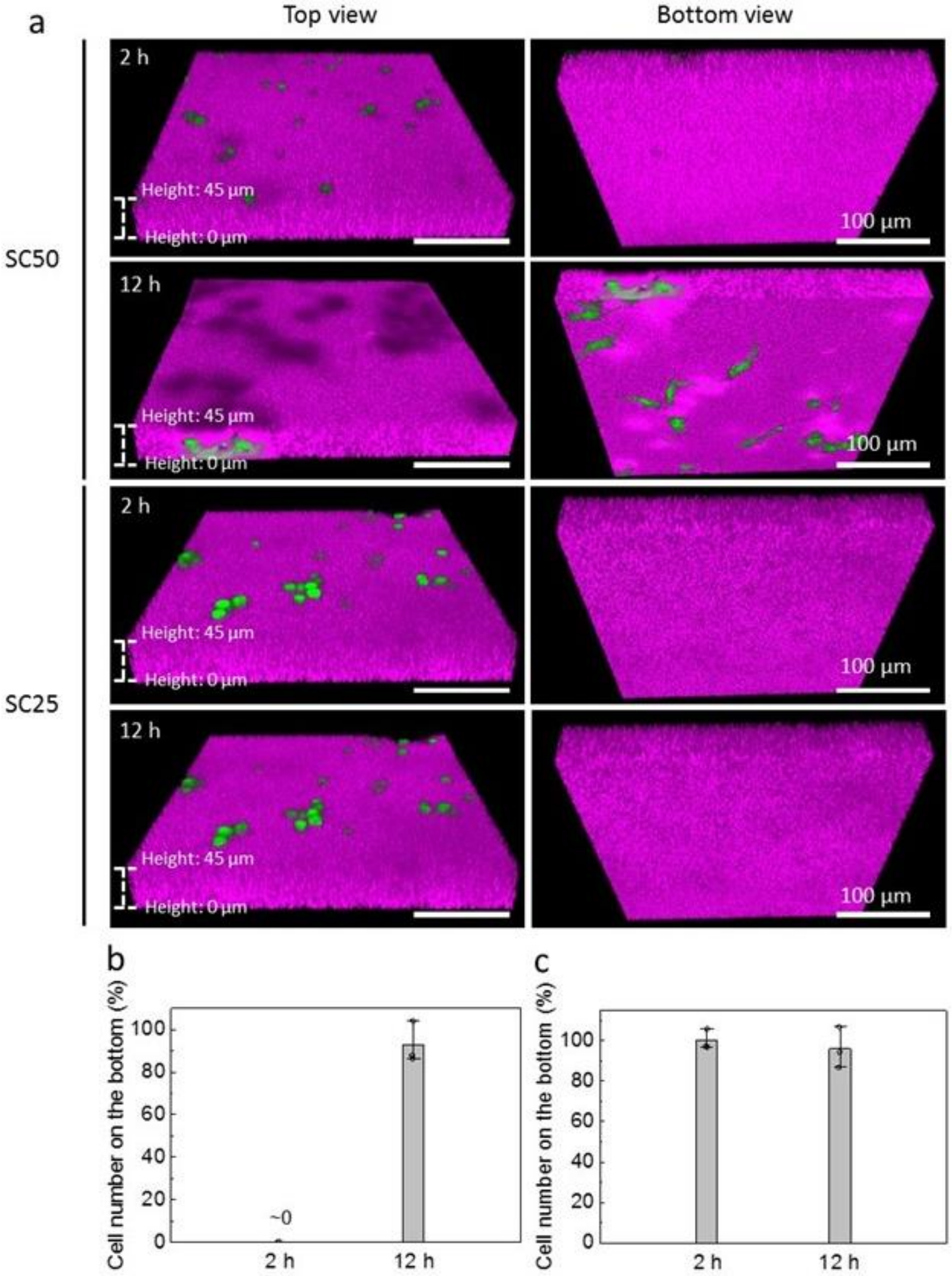

Supplementary Figure 45. REF52 cells transmigrate into fresh SC50 but cannot migrate through SC25. **a,** Fluorescence images of REF52 cells (green) cultivated on SC50 and SC25 (magenta) for 2 h and 12 h, respectively. Quantification of REF52 cells on different locations of (**b**) SC50 and (**c**) SC25 after 12 h cultivation, respectively. Note that cells migrated inside the SC50 with an apparent velocity of about 4.5 μm h^{-1}, whereas cells cannot migrate into SC25.

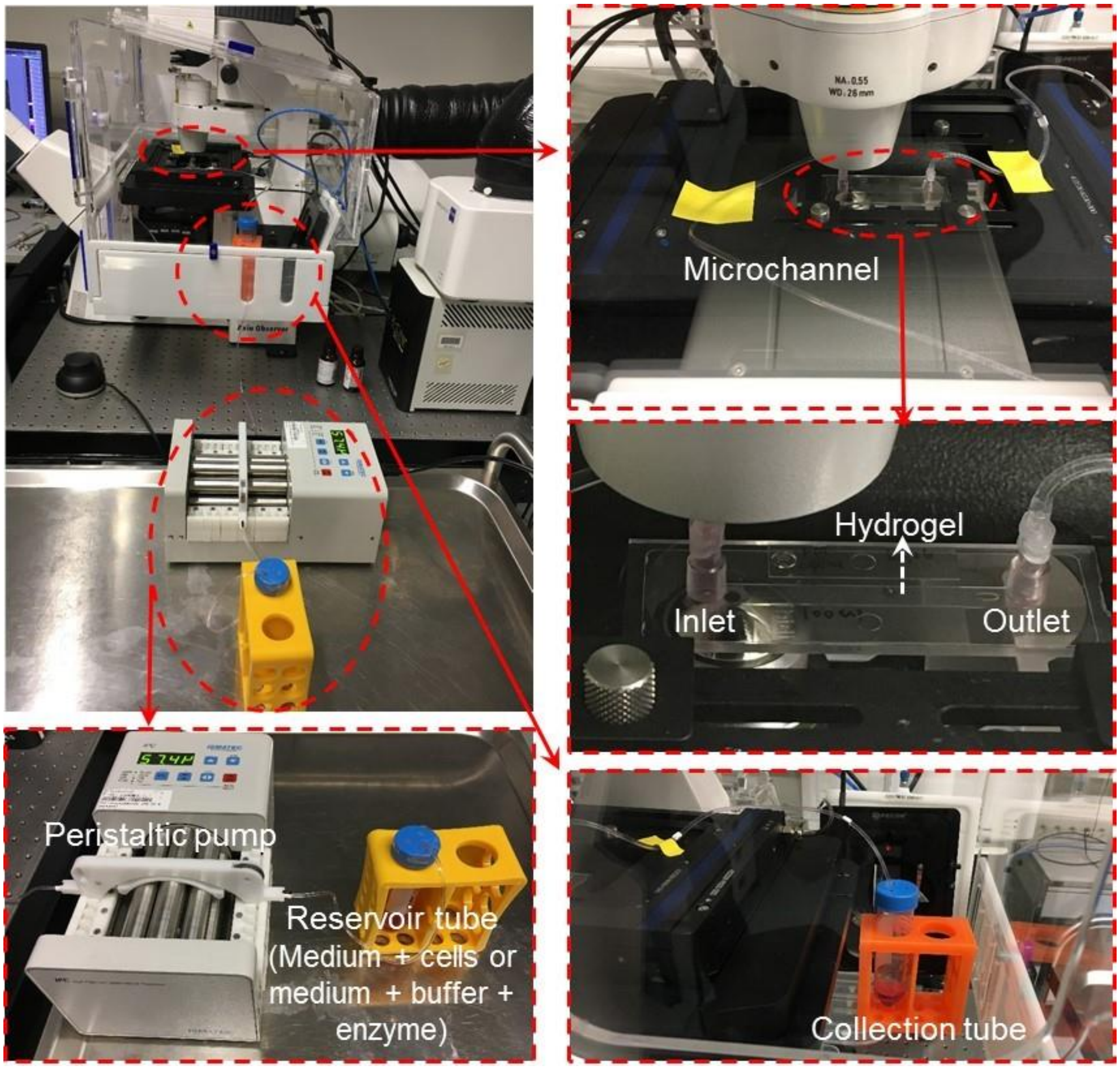

Supplementary Figure 46. Microfluidic setup for cell experiments. A microchannel chip coated with SC25 was used to trap $MCF7_{eGFP}$ in a microfluidic system. Medium was pumped from a reservoir tube through the microfluidic channel to the collection tube using a peristaltic pump.

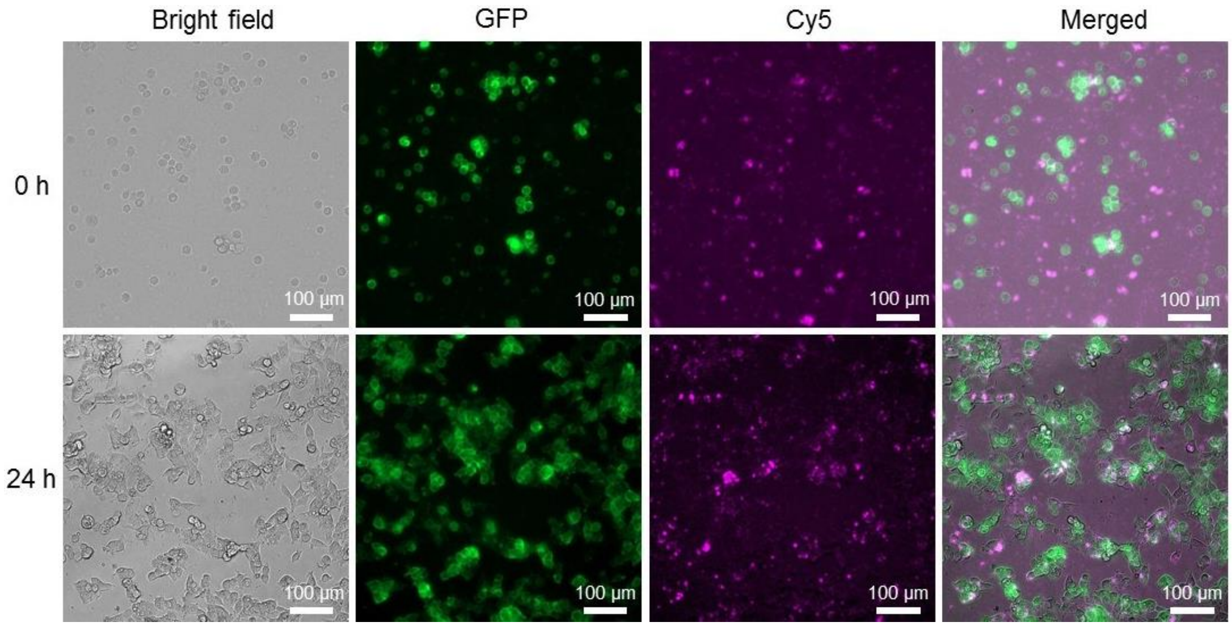

Supplementary Figure 47. Cultivation of $MCF7_{eGFP}$ cells released from SC25-coated microfluidic channel. $MCF7_{eGFP}$ cells collected from the outflow of the microfluidic channel (0 h) were cultured for an additional 24 h in a FN-coated coverslips. Note that the released $MCF7_{eGFP}$ cells adhere, spread, and proliferate, thereby suggesting that the cells were not harmed by growth in the flow channel and subsequent endonuclease treatment.

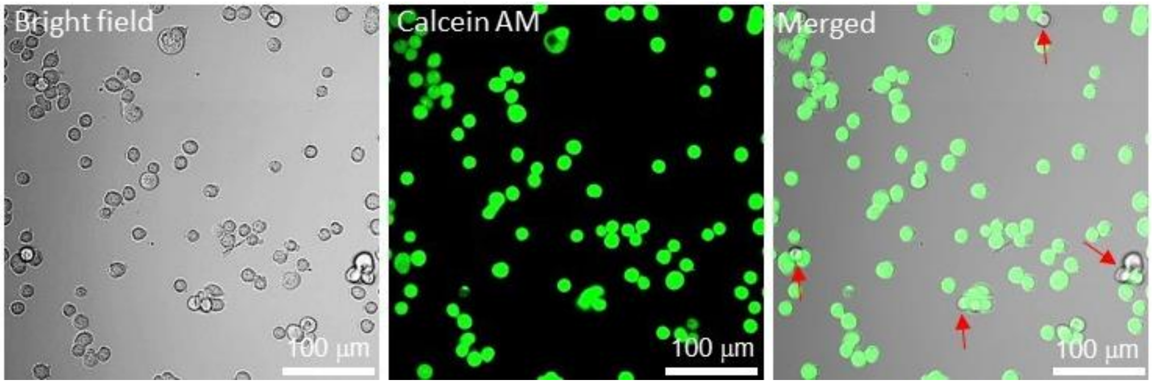

Supplementary Figure 48. Live cell staining of MCF7 cells released from an SC25-coated microfluidic channel. MCF7 cells collected from the outflow of the microfluidic channel after enzymatic restriction were cultured overnight on PLL-coated coverslips and subsequently stained with Calcein AM. Statistical analysis revealed that about 93% of the cells were stained by Calcein AM, thus indicating that enzymatic digestion in a microfluidic flow processes is gentle enough to not damage the cells.

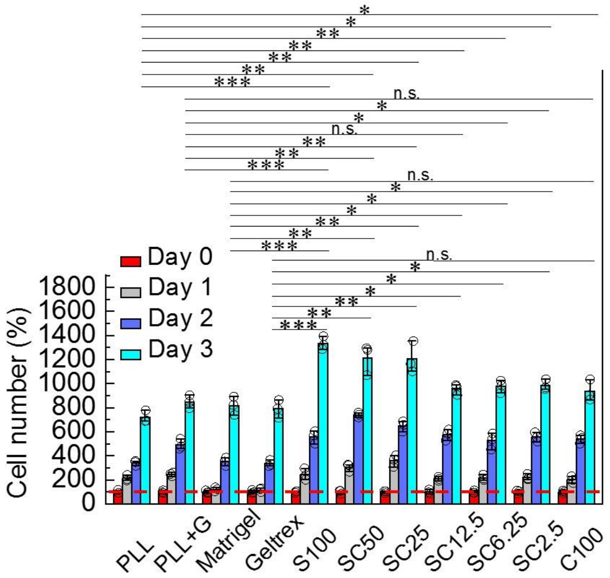

Supplementary Figure 49. Quantification of time-dependent mESC growth on various substrata. Full significance analysis of the quantification of time-dependent mESC growth on various substrata, normalized to data obtained from mESCs on PLL (red dashed line). This data supplements the significance tests shown in Figure 6a. Note that, except of SC2.5, the SiNP/CNT-DNA nanocomposites enable the expansion of mESCs with a higher efficiency than PLL, PLL+G, Matrigel, or Geltrex surfaces.

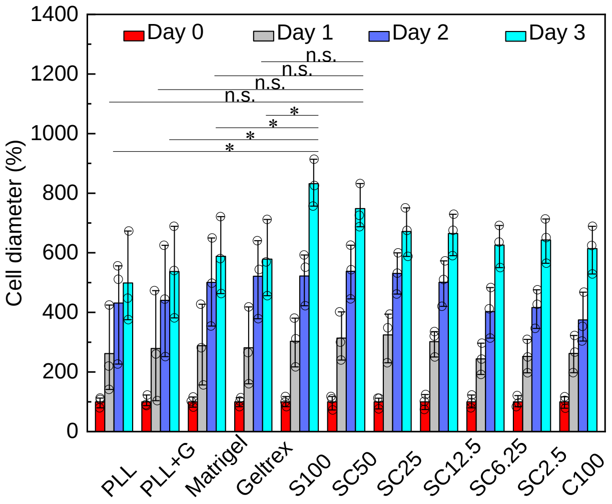

Supplementary Figure 50. Size distribution of mESCs on the various composite and control materials. mESC growth rates were quantified by measuring colony diameters for 3 days. Note that this data coincides very well with the data obtained by CCK-8 assays (Supplementary Figure 49 and main manuscript, Figure 6a).

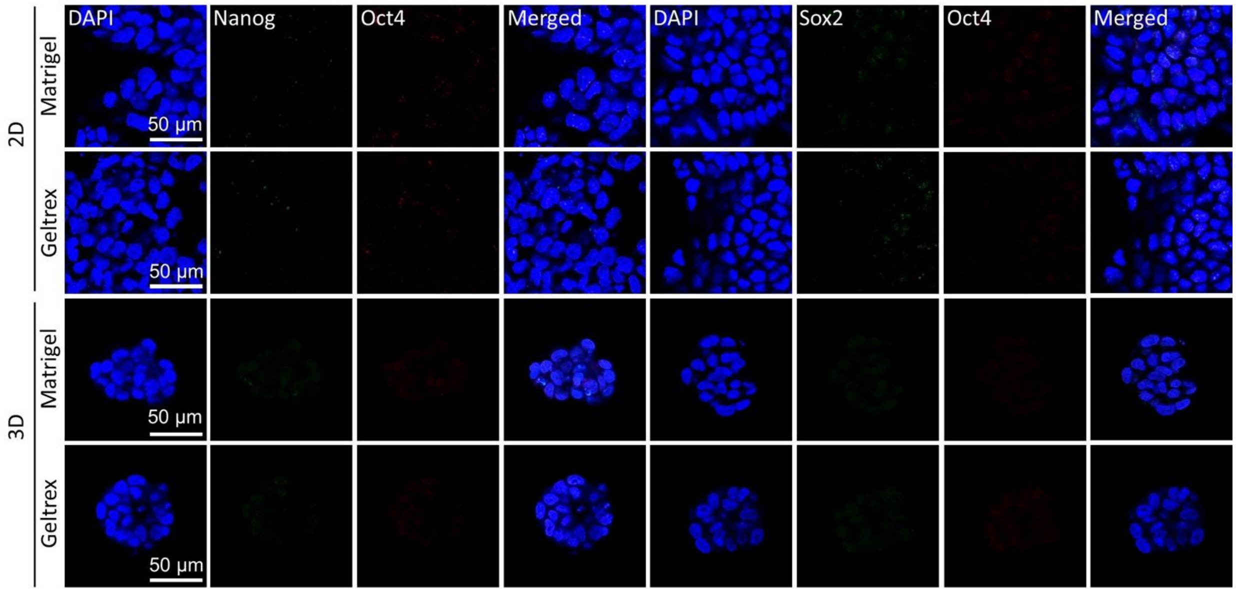

Supplementary Figure 51. Immunostaining of Nanog, Sox2 and Oct4 of mESCs grown on Matrigel and Geltrex. After cultivation on Matrigel or Geltrex for 4 days in the absence of LIF, mESCs were analyzed by immunostaining with primary polyclonal antibodies (pAb) IgG rabbit anti-Nanog/Sox2 and mAb IgG mouse anti-Oct4 and secondary antibodies (goat anti-rabbit IgG Alexa Fluor®488 and goat anti-mouse IgG Cy3). Note that, in absence of LIF, neither Matrigel nor Geltrex were able to preserve the stemness of mESCs in either 2D or 3D cell culture.

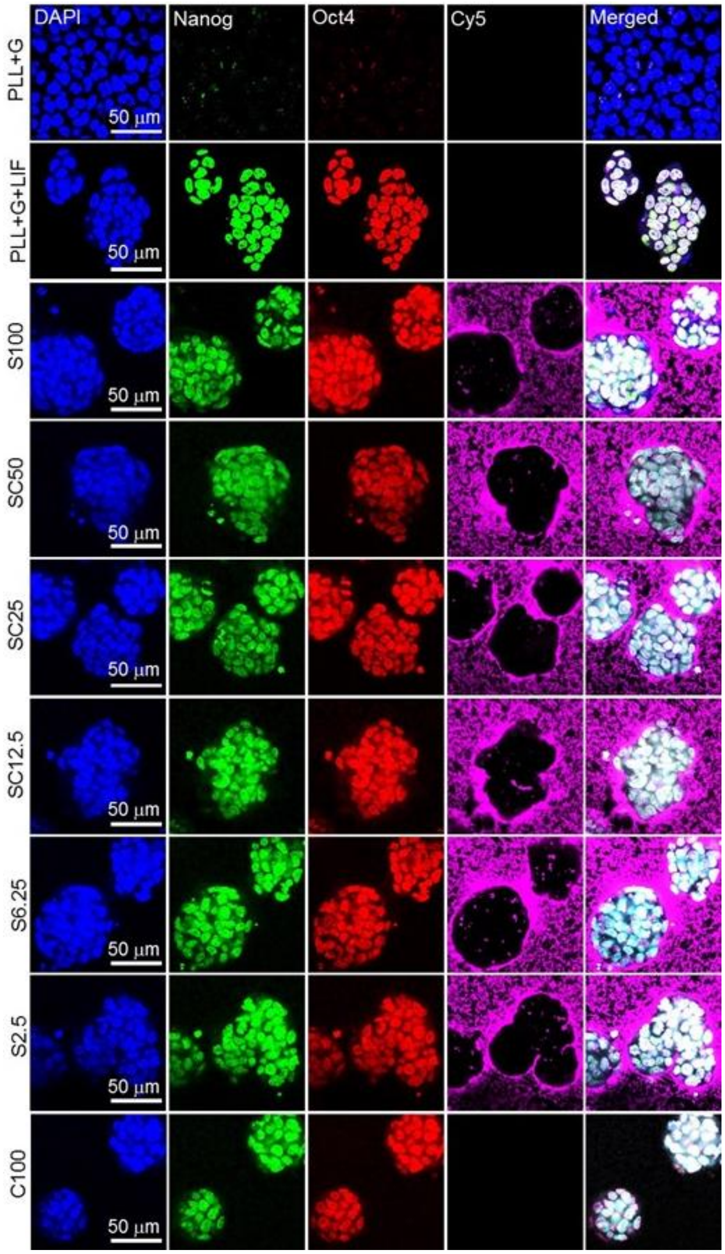

Supplementary Figure 52. Immunostaining of Nanog and Oct4 of mESCs grown on SiNP/CNT-DNA nanocomposite materials. After cultivation on fresh nanocomposite materials (Cy5, Magenta) for 4 days in the absence of LIF, mESCs were immunostained with primary polyclonal antibodies (pAb) rabbit anti-Nanog and mAb IgG mouse anti-Oct4) and secondary antibodies (goat anti-rabbit IgG Alexa Fluor®488 and goat anti-mouse IgG Cy3). mESCs grown on gelatine-coated PLL with and without LIF were used as controls. Note that mESCs differentiated and changed from spheroid morphologies to confluent monolayers after 4 days cultivation on gelatine-coated PLL without LIF (see first row of images in Supplementary Figures 52 and 53), while mESCs grown on the nanocomposite materials formed pluripotent 3D colonies.

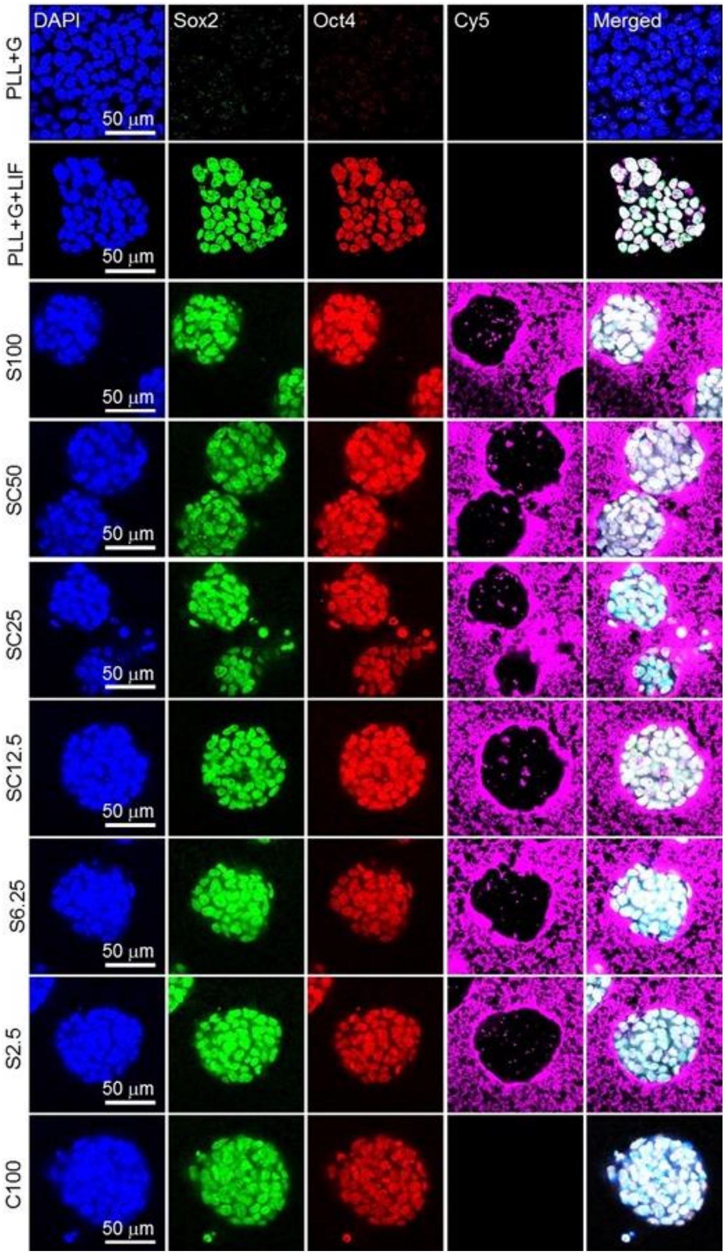

Supplementary Figure 53. Immunostaining of Sox2 and Oct4 of mESCs grown on SiNP/CNT-DNA nanocomposite materials. After cultivation on fresh nanocomposite materials (Cy5, Magenta) for 4 days in the absence of LIF, mESCs were immunostained with primary antibodies (pAb rabbit anti-Sox2 and mAb IgG mouse anti-Oct4) and secondary antibodies (goat anti-rabbit IgG Alexa Fluor®488 and goat anti-mouse IgG Cy3). mESCs grown on PLL+G in the presence or absence of LIF were used as controls.

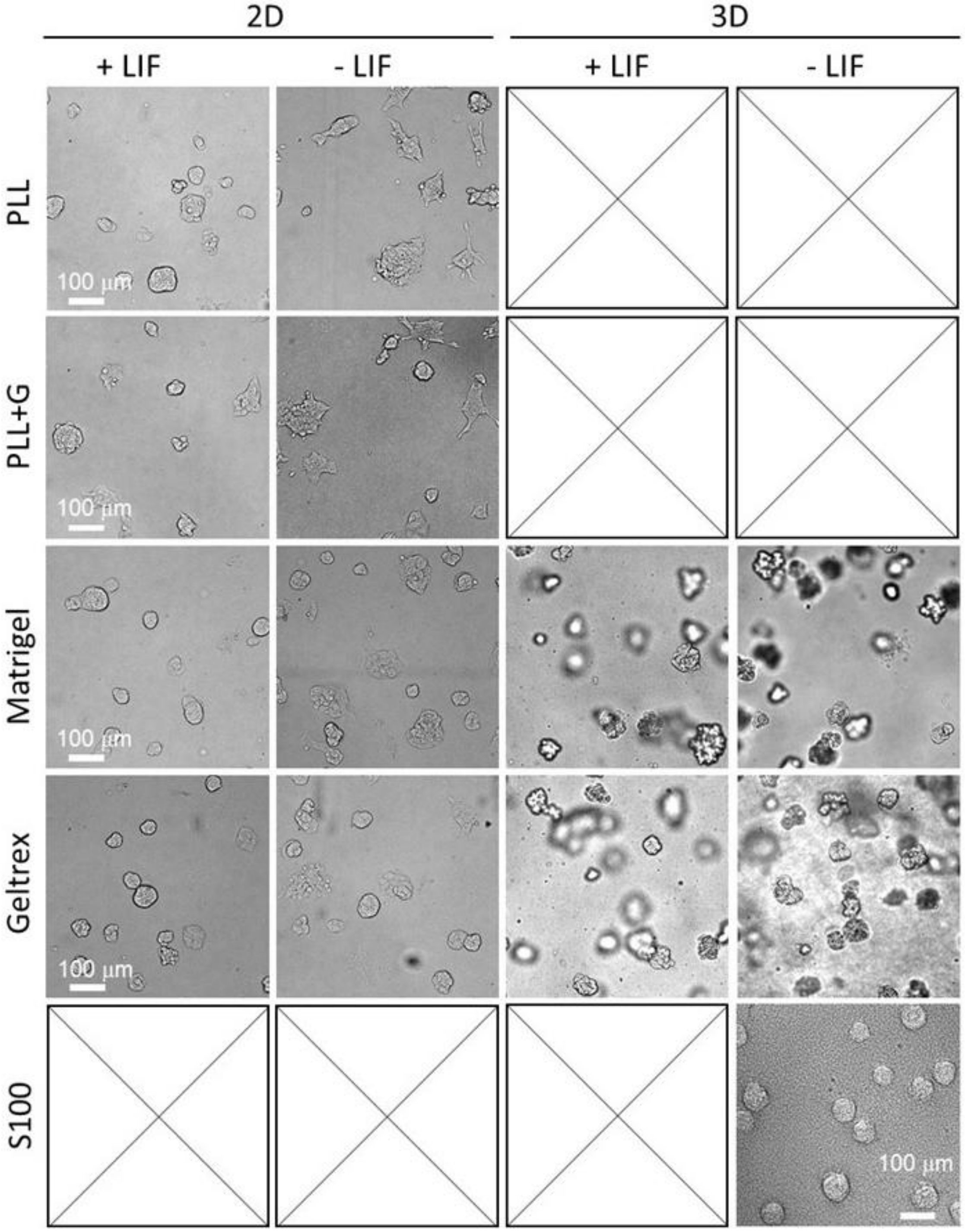

Supplementary Figure 54. Bright field imaging analysis of mESCs grown on PLL, PLL+G, Matrigel, Geltrex and S100. mESCs were grown on the various substrata for 3 days. Note that the spheroids grown in S100 have the desired uniform, concentric, compact and raised shape to a much greater extent than those grown on other substrates.

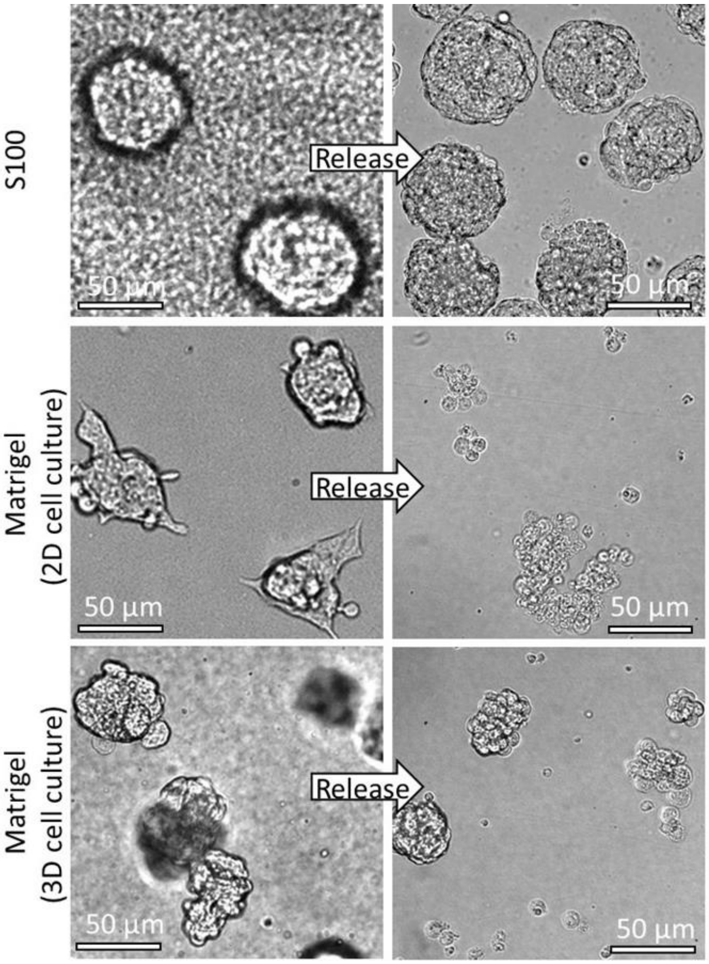

Supplementary Figure 55. Bright field imaging analysis of mESC colonies before and after release from culture materials. After growth of mESC for 4 days in the absence of LIF, spheroids were released from S100 *via* endonuclease digestion or from Matrigel by using the commercial ‘cell recovery solution’ (Corning), according to manufacturer’s instructions. Note that cell recovery from Matrigel requires > 4 h of 4 °C incubation as well as harsh pipetting and scraping, while endonuclease release was fully completed after 2 h of 37 °C incubation without any mechanical manipulation. Hence, release from S100 occurred without apparent damage of the spheroids.

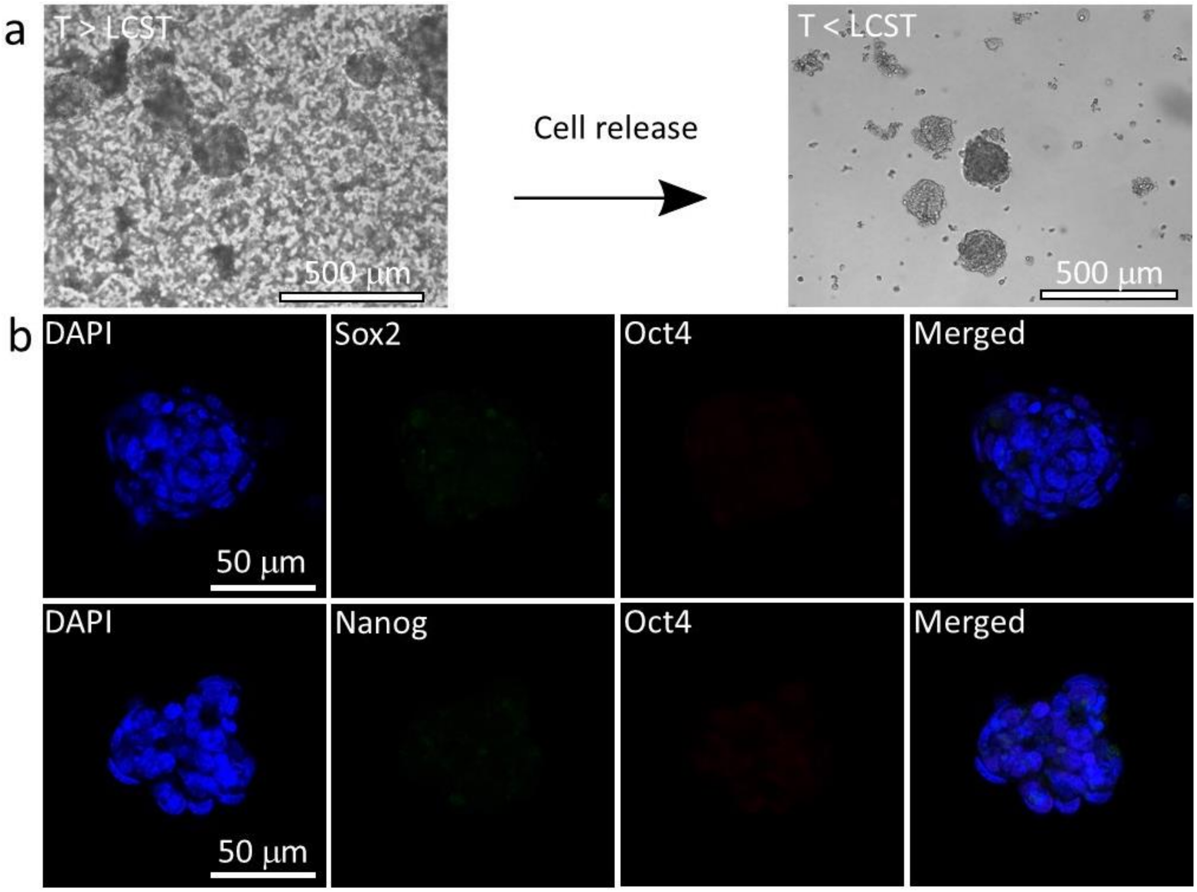

Supplementary Figure 56. Release of mESCs from pNIPAM surfaces. **a**, Release of the mESCs after growth on pNIPAM prepared according to the previous work[26] for 4 days at T = 37 °C, which is above the lower critical solution temperature (LCST). Cell release was achieved by cooling to room temperature. Note that the released colonies display a broad size distribution **b**, mESCs after cultivation on pNIPAM for 4 days in the absence of LIF were immunostained with primary pAb IgG rabbit anti-Nanog/Sox2 and mAb IgG mouse anti-Oct4 and secondary antibodies (goat anti-rabbit IgG Alexa Fluor®488 and goat anti-mouse IgG Cy3). Note that pluripotency is lost after 4 days cultivation on pNIPAM.

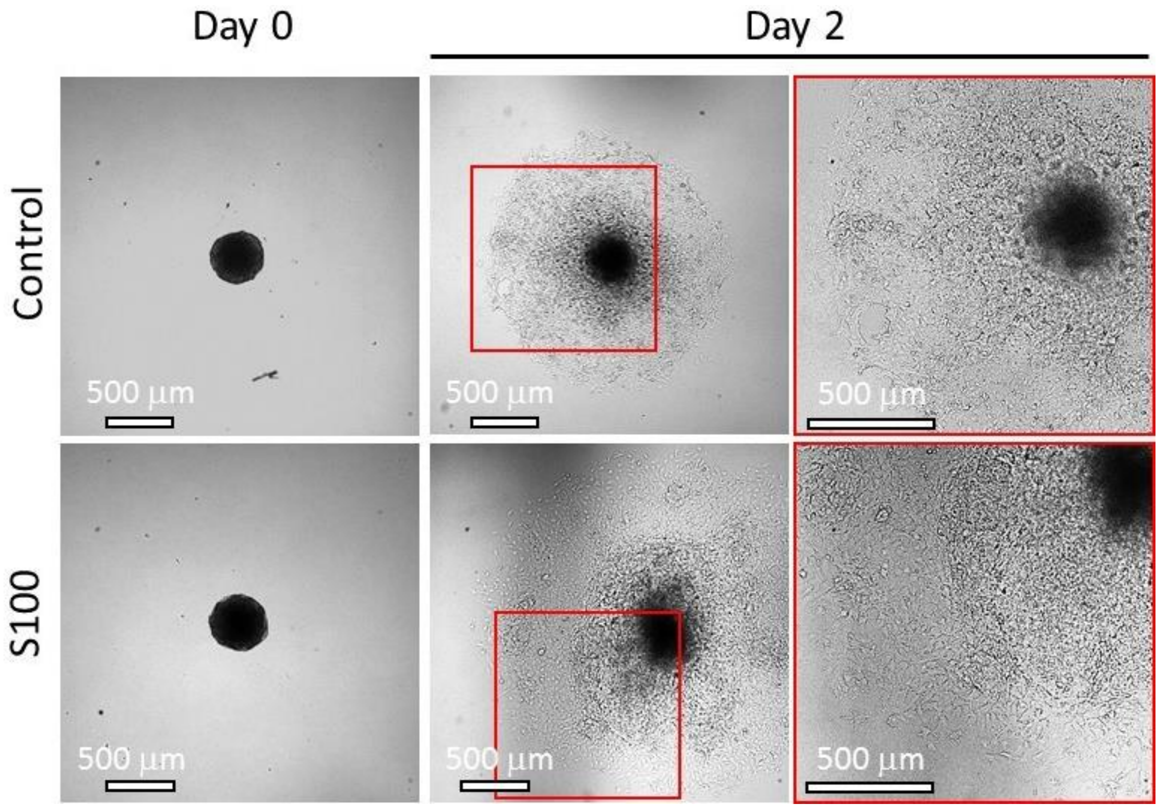

Supplementary Figure 57. Bright field images of mESC spheroid (embryoid body) extracted from S100. The mESC spheroids were grown in S100 for 4 days, enzymatically released and transferred into a U shaped plate for further growth for another 3 days. The spheroids were then replated on PLL-coated coverslips. As a positive control, similarly sized spheroids (embryoid bodies, EBs) were prepared by routine culture of mESCs using the hanging drop method[27]. Note that, after adhesion of the free-floating EBs (Day 0) to the PLL substrate, cellular migration out of the differentiating EBs periphery occurred (Day 2).

Supplementary Table 6. List of DNA sequences.

Name	Sequence (5'-3')	Modification
P1	TCTAACTGCTGCGCCGCCGGGAAAATACTGTACGGTTAGA	-
aP1	[AmC12]TCTAACTGCTGCGCCGCCGGGAAAATACTGTACGG TTAGA	5'Amine C12
P2	TTTTTTTTTTTTTTTTTTTTTTTTTTTTTTTTTTTTTTTTTCTAA CTGCTGCGCCGCCGGGAAAATACTGTACGGTTAGA	-
M1	[AmC12]CTACTAGACATCTGTATCTCTCGCGGCGCGGAAGC TATAG	5'Amine C12
M2	TTTTTTTTTTTTTTTTTTTTTTTTTTTTTTTTTTTTTTTTTCTACT AGACATCTGTATCTCTCGCGGCGCGGAAGCTATAG	-
M3	ACGTGACTCATCGATGCGTCGTAGCCTCCTC	-
M4	GAGGAGGCTACGACGCATCGATGAGTCACGT	-
Cy3-cP1	[Cyanine3]TCTAACCGTACAGTATTTTCCCGGCGGCGCAGCA GTTAGA	5' Cyanine3
T **(**BstEII restriction site**)**	[Phos]TTCCCGGCGGCGCAGCAGTTAGATGCTGCTGCAGCGA TACGCGTATCGCTATGGGTAACCGTACGGTTACCCGCAGCA GCATCTAACCGTACAGTATT	5' Phosphorylation

References

1. Leidner, A. et al. Biopebbles: DNA‐Functionalized Core–Shell Silica Nanospheres for Cellular Uptake and Cell Guidance Studies. *Adv. Funct. Mater.* **28**, 1707572 (2018).
2. Dresselhaus, M.S., Jorio, A., Hofmann, M., Dresselhaus, G. & Saito, R. Perspectives on carbon nanotubes and graphene Raman spectroscopy. *Nano Lett.* **10**, 751-758 (2010).
3. Costa, S., Borowiak-Palen, E., Kruszynska, M., Bachmatiuk, A. & Kalenczuk, R. Characterization of carbon nanotubes by Raman spectroscopy. *Mater. Sci.-Pol.* **26**, 433-441 (2008).
4. Souza Filho, A. et al. Stokes and anti-Stokes Raman spectra of small-diameter isolated carbon nanotubes. *Phys. Rev. B* **69**, 115428 (2004).
5. Fantini, C. et al. Optical transition energies for carbon nanotubes from resonant Raman spectroscopy: Environment and temperature effects. *Phys. Rev. Lett.* **93**, 147406 (2004).
6. Prescott, B., Steinmetz, W. & Thomas, G. Characterization of DNA structures by laser Raman spectroscopy. *Biopolymers* **23**, 235-256 (1984).
7. Liu, Y. et al. Fluorescent assay of DNA hybridization with label-free molecular switch: reducing background-signal and improving specificity by using carbon nanotubes. *Chem. Commun.*, 665-667 (2009).
8. Rouwkema, J., Koopman, B.F.J.M., Blitterswijk, C.A.V., Dhert, W.J.A. & Malda, J. Supply of Nutrients to Cells in Engineered Tissues. *Biotechnol Genetic Engin Rev* **26**, 163-178 (2009).
9. Zorlutuna, P., Jeong, J.H., Kong, H. & Bashir, R. Stereolithography-Based Hydrogel Microenvironments to Examine Cellular Interactions. *Adv Funct Mater* **21**, 3642-3651 (2011).

10. Soumpasis, D. Theoretical analysis of fluorescence photobleaching recovery experiments. *Biophys. J.* **41**, 95-97 (1983).
11. Brandl, F. et al. Hydrogel-based drug delivery systems: comparison of drug diffusivity and release kinetics. *J Control Release* **142**, 221-228 (2010).
12. Rosa, M., Dias, R., da Graça Miguel, M. & Lindman, B. DNA−Cationic Surfactant Interactions Are Different for Double- and Single-Stranded DNA. *Biomacromolecules* **6**, 2164-2171 (2005).
13. Studdert, D.S., Patroni, M. & Davis, R.C. Circular dichroism of DNA: temperature and salt dependence. *Biopolymers* **11**, 761-779 (1972).
14. Zhu, G. et al. Noncanonical self-assembly of multifunctional DNA nanoflowers for biomedical applications. *J. Am. Chem. Soc.* **135**, 16438-16445 (2013).
15. Angelin, A. et al. Multiscale Origami Structures as Interface for Cells. *Angew Chem Int Ed Engl* **54**, 15813-15817 (2015).
16. Drelich, J., Chibowski, E., Meng, D.D. & Terpilowski, K. Hydrophilic and superhydrophilic surfaces and materials. *Soft Matter* **7**, 9804-9828 (2011).
17. Turner, C.E. Paxillin and focal adhesion signalling. *Nat Cell Biol* **2**, E231 (2000).
18. Robertson, J. et al. Defining the phospho-adhesome through the phosphoproteomic analysis of integrin signalling. *Nat Commun* **6**, 6265 (2015).
19. Arnold, M. et al. Cell interactions with hierarchically structured nano-patterned adhesive surfaces. *Soft Matter* **5**, 72-77 (2009).
20. Pallarola, D. et al. Interface immobilization chemistry of cRGD‐based peptides regulates integrin mediated cell adhesion. *Adv. Funct. Mater.* **24**, 943-956 (2014).
21. Arima, Y. & Iwata, H. Effect of wettability and surface functional groups on protein adsorption and cell adhesion using well-defined mixed self-assembled monolayers. *Biomaterials* **28**, 3074-3082 (2007).
22. Seliktar, D. Designing cell-compatible hydrogels for biomedical applications. *Science* **336**, 1124-1128 (2012).
23. Ross, A.M., Jiang, Z., Bastmeyer, M. & Lahann, J. Physical aspects of cell culture substrates: topography, roughness, and elasticity. *Small* **8**, 336-355 (2012).
24. DiMilla, P.A., Stone, J.A., Quinn, J.A., Albelda, S.M. & Lauffenburger, D.A. Maximal migration of human smooth muscle cells on fibronectin and type IV collagen occurs at an intermediate attachment strength. *J. Cell Biol.* **122**, 729-737 (1993).
25. Podesta, F., Roth, T., Ferrara, F., Cagliero, E. & Lorenzi, M. Cytoskeletal changes induced by excess extracellular matrix impair endothelial cell replication. *Diabetologia* **40**, 879-886 (1997).
26. Hippler, M. et al. Controlling the shape of 3D microstructures by temperature and light. *Nat Commun* **10**, 232 (2019).
27. Wang, X. & Yang, P. In vitro differentiation of mouse embryonic stem (mES) cells using the hanging drop method. *JoVE*, e825 (2008).
