# Supplementary material for: Carbon-nanotube reinforcement of DNA-silica nanocomposites yields programmable and cell-instructive biocoatings
Source: Nat Commun. 2019 Dec 4;10:5522. doi: 10.1038/s41467-019-13381-1 (PMC6892801; doi:10.1038/s41467-019-13381-1)
Supplement: Supplementary file 2 — Description of Additional Supplementary Files [file 41467_2019_13381_MOESM2_ESM.pdf]

Description of Additional Supplementary Files

Supplementary Movie 1: Life cell imaging of MCF7eGFP cells seeded on PLL surface. Life cell imaging was observed from 0 h to 24 h. Frame interval time: 36 min; frame rate: 5fps.

Supplementary Movie 2: Life cell imaging of MCF7eGFP cells seeded on dried SC50 surface. Life cell imaging was observed from 0 h to 24 h. Frame interval time: 36 min; frame rate: 5fps.

Supplementary Movie 3: Life cell imaging of MCF7eGFP cells adhered on fresh SC25 inside a microfluidic channel before and after treatment with restriction enzyme BstEII-HF. This movie starts at the image t = 0, shown in Figure 5c. Frame interval time: 10 min; frame rate: 5fps.
